# Adaptive mechanisms in pancreatic islets counteract mitochondrial dysfunction in Barth syndrome

**DOI:** 10.1007/s00125-025-06575-4

**Published:** 2025-10-24

**Authors:** Christopher Carlein, Markus D. A. Hoffmann, Caroline Bickelmann, Andressa G. Amaral, Ahmadali Lotfinia, Laurie-Anne de Selliers, Johanne Audouze-Chaud, Selina Wrublewsky, Marcel A. Lauterbach, Karina von der Malsburg, Martin van der Laan, Monika Bozem, Markus Hoth, Patrick Gilon, Magalie A. Ravier, Bruce Morgan, Emmanuel Ampofo, Takahiro Higuchi, Edoardo Bertero, Jan Dudek, Christoph Maack, Leticia Prates Roma

**Affiliations:** 1 Department of Biophysics, Center for Integrative Physiology and Molecular Medicine (CIPMM), Center for Human and Molecular Medicine (ZHMB), Center for Gender-specific Biology and Medicine (CGBM), Faculty of Medicine, Saarland University, Homburg, Germany; 2 Institute for Clinical and Experimental Surgery, Saarland University, Homburg, Germany; 3 Biomedical Sciences Institute, University of Sao Paulo, Sao Paulo, Brazil; 4 Department of Molecular Imaging, Center for Integrative Physiology and Molecular Medicine, Saarland University, Homburg, Germany; 5 Department of Medical Biochemistry and Molecular Biology, Center for Molecular Signaling, PZMS, Faculty of Medicine, Saarland University, Homburg, Germany; 6 Department of Biophysics, Center for Integrative Physiology and Molecular Medicine (CIPMM), Faculty of Medicine, Saarland University, Homburg, Germany; 7 Université Catholique de Louvain, Institut de Recherche Expérimentale et Clinique, Pôle d’Endocrinologie, Diabète et Nutrition, Brussels, Belgium; 8 IGF, Univ. Montpellier, CNRS, Inserm, Montpellier, France; 9 Institute of Biochemistry, Center for Human and Molecular Biology (ZHMB), Saarland University, Saarbrücken, Germany; 10 Department of Nuclear Medicine, University Hospital Würzburg, Würzburg, Germany; 11 Department of Translational Research, Comprehensive Heart Failure Center, University Clinic, Würzburg, Germany; 12 IRCCS Ospedale Policlinico San Martino, Genova, Italy; 13 Medical Clinic I, University Clinic, Würzburg, Germany

**Keywords:** Barth syndrome, Cardiolipin, Mitochondria, *O*-GlcNAc, Pancreatic islets, Tafazzin

## Abstract

**Aims/hypothesis:**

Barth syndrome is a mitochondrial disorder caused by *Tafazzin* (*TAZ*) mutations, which impair cardiolipin remodelling and contribute to systemic metabolic alterations. While islet dysfunction has been implicated in Barth syndrome, its underlying mechanisms remain unknown. We aimed to determine how Tafazzin (Taz) deficiency affects mouse pancreatic islet metabolism and hormone secretion, and whether systemic signals, such as circulating factors, modulate these effects in vivo. In vivo and in vitro models were used to separate direct islet effects from systemic influences of Taz deficiency.

**Methods:**

We used a mouse model of global *Taz* knockdown (*Taz*-KD) and combined in vivo and in vitro approaches to assess pancreatic islet metabolism, morphology and hormone secretion. Islet function was evaluated under basal and glucotoxic conditions. Transcriptomic profiling was performed to identify gene expression changes in isolated islets from *Taz*-KD mice and following in vitro *Taz*-KD. Additionally, we examined the role of the circulating factor fibroblast growth factor 21 (FGF-21) in modulating islet function.

**Results:**

Despite impaired cardiolipin remodelling, pancreatic islets from *Taz*-KD mice maintained insulin secretion, supported by compensatory mechanisms such as increased glucose uptake, expanded mitochondrial volume and increased metabolic parameters. In addition, alpha cell mass and glucagon secretion were significantly increased in *Taz*-KD islets. These islet-specific adaptations occurred alongside improved whole-body glucose tolerance, elevated circulating FGF-21 levels and enhanced glucose uptake in brown adipose tissue. In contrast, in vitro *Taz*-KD led to impaired islet function and reduced insulin secretion. Transcriptomic analysis revealed distinct gene expression patterns between in vivo and in vitro *Taz*-KD models. While in vivo upregulation of genes related to *N*-acetylglucosamine biosynthesis and *O*-GlcNAcylation were related to compensatory mechanisms, in vitro *Taz*-KD affected, among others, the MAPK pathway, contributing to islet dysfunction. Notably, islet incubation with FGF-21 was able to restore insulin secretion after in vitro *Taz*-KD.

**Conclusions/interpretation:**

Our findings demonstrate that while *Taz* and cardiolipin remodelling are essential for beta cell physiology, systemic and islet-specific compensatory mechanisms preserve insulin secretion in vivo in *Taz*-KD mice, alongside increased glucagon secretion. These adaptations probably contribute to the altered metabolic phenotype observed in Barth syndrome and highlight a potential role for hormones and circulating factors such as FGF-21 in maintaining islet function and glucose homeostasis.

**Graphical Abstract:**

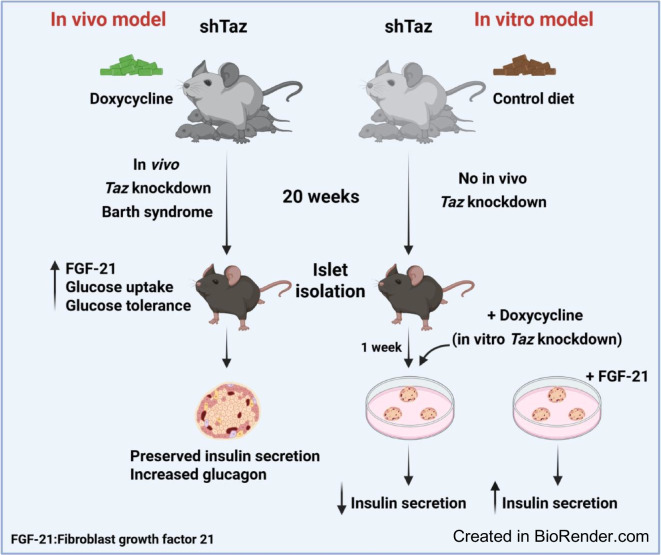

**Supplementary Information:**

The online version contains peer-reviewed but unedited supplementary material available at 10.1007/s00125-025-06575-4.


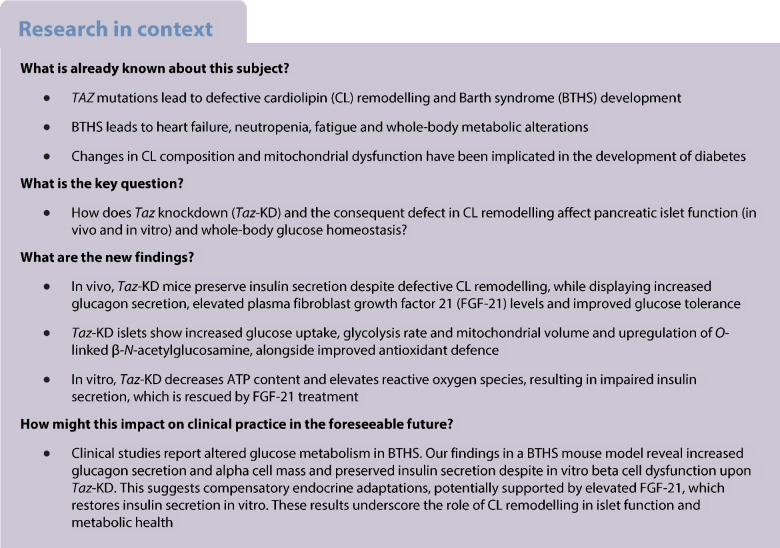


## Introduction

Barth syndrome (BTHS) is a life-threatening, X-linked multisystem disorder characterised by pleiotropic phenotypes including heart failure, growth delay, skeletal myopathy and neutropenia [1]. Individuals with BTHS also show changes in whole-body fatty acid, glucose and amino acid metabolism [2]. BTHS is caused by mutations in the *Tafazzin* (*TAZ*) gene, which encodes Tafazzin (TAZ), a mitochondrial phospholipid:lysophospholipid transacylase essential for cardiolipin (CL) remodelling [1]. The direct consequence of *TAZ* mutation is the decrease in mature CL and the accumulation of monolysocardiolipin (MLCL), a precursor of CL, which has been shown to have a lower affinity for the respiratory chain complexes III and IV [3, 4]. Changes in CL content or composition, i.e. the identity of the associated fatty acids, correlate with defective formation of mitochondrial supercomplexes, aberrant cristae formation and shape, decreased respiration, reduced ATP production and increased reactive oxygen species (ROS) production [4–7]. Other cellular functions, including mitophagy and apoptosis, were also shown to be affected by changes in CL [8]. However, the consequences of defective CL remodelling are tissue-specific and many are still unknown or unclear, with contradictory reports found in the published literature [9–11]. For example, increased mitochondrial ROS production has been suggested to have a causal relationship with cellular dysfunction in BTHS, while others have reported no changes and no significant impact in the disease [9, 12–14]. We recently showed that mitochondrial calcium uptake is strongly decreased in cardiomyocytes from *Taz* knockdown (*Taz*-KD) mice due to reduced levels of the mitochondrial calcium uniporter (MCU). Consequently, Krebs cycle activation and mitochondrial respiration during β-adrenergic stimulation is impaired, leading to a lack of inotropic reserve in BTHS cardiomyopathy [7, 9]. We did not observe any in vivo changes in cardiomyocyte H_2_O_2_ levels, probably due to increased antioxidant defence [9], which may be driven by eukaryotic initiation factor 2 alpha subunit (eIF2α)/activating transcription factor 4 (ATF4)-mediated upregulation of one-carbon metabolism and a subsequent increase in glutathione production [15].

Metabolic disorders are associated with absolute changes in fatty acid levels as well as changes in the relative abundance of fatty acids with different chain lengths and saturation. Changes are also seen in the fatty acid profile and abundance of glycerophospholipids, including CL. For instance, in a streptozocin (STZ)-induced diabetic mouse model, CL content in the myocardium was markedly reduced and showed significant remodelling, characterised by a shift from 18:2-enriched CL species to those enriched in 22:6 fatty acids. Similar alterations were observed in the myocardium of obese *ob/ob* mice [16, 17]. These observations suggest a vital role of CL content and fatty acid composition in maintaining metabolic functions and a possible role in the development of metabolic diseases [17]. Consistent with this hypothesis, CL was shown to be important for whole-body energy homeostasis by regulating non-shivering thermogenesis and CL levels were shown to positively correlate with insulin sensitivity [18]. Interestingly, *Taz-*KD mice are resistant to diet-induced obesity and are protected against hepatic steatosis [19], again supporting a role of Taz and CL in whole-body metabolism. Individuals with BTHS display recurrent hypoglycaemia and disrupted fatty acid and amino acid metabolism [20, 21]. Furthermore, studies on both *Taz*-KD mice and samples from individuals with BTHS showed increased glucose utilisation in a number of tissues, including lymphocytes and cardiomyocytes, compared with healthy control groups [15, 22].

Pancreatic islets are key players in maintaining whole-body energetic balance and glucose homeostasis, harbouring the cells that secrete insulin (beta cells), glucagon (alpha cells) and somatostatin (delta cells). Recently, in vivo *Taz* deficiency was reported to lead to decreased islet insulin secretion and oxygen consumption, an effect that was significant in low-glucose concentrations [23]. However, this observation does not appear to be consistent with the occurrence of frequent hypoglycaemic episodes in humans and raises questions about the role of pancreatic hormones in BTHS metabolic phenotypes.

In this study, we systematically investigated the impact of *Taz*-KD on the secretory pathway of pancreatic islets. Using an in vivo *Taz*-KD mouse model, we aimed to explore the physiological and metabolic adaptations that arise in response to *Taz* deficiency, with a particular focus on pancreatic islet function. Additionally, we examined potential compensatory mechanisms that may influence cellular metabolism, glucose handling and hormonal secretion. To further dissect the role of *Taz* in pancreatic islets, we also employed an in vitro model of islet-specific *Taz*-KD to assess its effects on cellular energy balance and insulin secretion. Through these approaches, we sought to gain deeper insights into the interplay between mitochondrial function, glucose metabolism and pancreatic endocrine regulation in the context of BTHS.

## Methods

### Animal models and genotyping

All animal experiments were approved by the local authorities (animal experiment approval 08/2018 and 19/2019) and in accordance with the Society of Laboratory Animal Science (GV-SOLAS) guidelines, following the ‘Replacement, Reduction, Refinement’ (3R) principles. Male and female mice were used.

#### shTaz

The *Taz*-KD mouse model was obtained from The Jackson Laboratory (B6.Cg-Gt(ROSA)26Sor^tm37(H1/tet0−RNAi:Taz)Arte^/ZkhuJ, stock number: 014648). Doxycycline (doxy) in a concentration of 625 mg of doxy/kg was added to the standard rodent chow (A153D70623, Ssniff, Germany) leading to induction of short hairpin RNA (shRNA)-mediated knockdown of *Taz*, as described previously [24].

#### shTaz × mito-roGFP2-Orp1

The mitochondria-redox-sensitive green fluorescent protein 2–oxidant receptor peroxidase 1 (mito-roGFP2-Orp1) mouse strain (first described in [25], a kind gift of T. Dick [Redox Regulation, DKFZ, Heidelberg]), which globally expresses an H_2_O_2_ sensor (ROSA26/CAG-stop^fl^-mito-roGFP2-Orp1 × CMV-Cre) targeted to the mitochondrial matrix, was crossbred with the *Taz*-KD and corresponding wild-type (WT) mice.

Mouse genotypes were confirmed using protocols and primers described in the electronic supplementary material (ESM). See the ESM for further details.

### Pancreatic islet isolation and culture

Pancreatic islets were isolated from mice by collagenase digestion via pancreatic duct perfusion, followed by digestion at 37°C and washing in Krebs–Henseleit buffer (KHB). Islets were hand-picked under a stereo microscope and cultured in RPMI 1640 medium supplemented with 10% FBS and 1% penicillin/streptomycin (P/S) at 37°C and 5% CO_2_. Islets were cultured for 1–3 days for the in vivo model or 48 h to 1 week for the in vitro model. Islets (smaller than 150–200 μm) were carefully selected for longer periods of culture to avoid central necrosis as shown before [26, 27]. Groups of size-matched WT and *Taz*-KD islets were prepared for experiments. See the ESM for further details.

### RNA isolation and quantitative real-time PCR

Total RNA was extracted from groups of approximately 150 pancreatic islets using TRIzol reagent and stored at −80°C. Isolated RNA was either used for RNA-seq or reverse-transcribed to cDNA. Quantitative real-time PCR was performed using TaqMan assays for *Taz* and *Gapdh* on a CFX96 Touch thermocycler. Primer details are provided in ESM Table 1. See the ESM for further details.

### Sample preparation for lipidomics and enzymatic assays

Groups of 600 islets per lipidomics sample and varying amounts for enzymatic assays were collected by pooling all pancreatic islets from 2–3 animals with the same genotype and sex. After culture, islets were homogenised by dispersion in PBS with additional sonication and centrifugation. See the ESM for further details.

### Lipidomics

Lipid extraction and analysis were performed by Lipotype Lipidomics (Dresden, Germany) using established protocols. Briefly, lipids were extracted with chloroform/methanol in the presence of internal standards covering major lipid classes. Extracts were dried and re-suspended in ammonium formate-containing solvent. Lipid profiling was conducted by high-resolution direct infusion MS (QExactive with TriVersa NanoMate) in both ion modes, combining MS and MS/MS. Data were processed with LipidXplorer, and only species with signal-to-noise >5 and intensities ≥5× above blanks were included. See the ESM for further details.

### GTT and plasmatic FGF-21 levels

Mice were fasted for 6 h and then injected intraperitoneally with glucose (2.2 mg/g body weight). Blood glucose levels were measured at 0, 7, 15, 30, 60 and 120 min using a glucometer. In selected experiments, blood samples were collected at each time point for plasma isolation. Analyses of plasma glucagon and insulin levels were performed with the corresponding mouse plasma insulin (homogeneous time-resolved fluorescence [HTRF] insulin mouse serum kit, ref.: 62IN3PEF, Cisbio/Perkin Elmer) and glucagon (mouse glucagon ELISA kit, ref.: 81518, Crystal Chem) kits. Plasma fibroblast growth factor 21 (FGF-21) was measured using the mouse FGF-21 ELISA kit by Abcam (Mouse FGF-21 ELISA Kit ab212160).

### [^18^F]FDG positron emission tomography

For the analysis of in vivo glucose metabolism in brown adipose tissue (BAT), animals were fasted for 12 h prior to imaging. Radiotracer administration was performed via i.p. injection of 7–15 MBq [^18^F]fluorodeoxyglucose ([^18^F]FDG). Anaesthesia was initiated 5 min prior to imaging using 2% isoflurane. Positron emission tomography (PET) imaging was performed using the Inveon PET System (Siemens Medical Solutions). Static 30 min PET imaging, focused on the upper chest region, was acquired from 60 to 90 min post injection. Computed tomography (CT) scans were obtained using the U-SPECT system (U-SPECT5/CT E-Class; MILabs) for anatomical reference. Following PET and CT imaging, postmortem tissue samples of BAT were collected and counted using a Wizard Gamma Counter (PerkinElmer, Waltham, MA).

### Immunohistochemistry

Pancreatic islet cell composition was assessed by immunohistochemistry (IHC) in both in vivo and in vitro *Taz*-KD models using cryosections and paraffin-embedded samples. For cryosections, pancreases were fixed in 4% paraformaldehyde (PFA), cryoprotected in 30% sucrose, embedded in optimal cutting temperature (OCT) compound and sectioned at 5 µm. Sections were stained for insulin, glucagon or somatostatin, with DAPI for nuclear counterstaining. For paraffin sections, fixed whole pancreases or clotted islets were processed and stained for alpha, beta and delta cell markers, Ki67 and cleaved caspase-3. Imaging was performed using the Axio Observer 7 (Zeiss, Germany), and quantification was done with ImageJ (National Institutes of Health, USA, version 2.3.0). See the ESM for further details.

### Static insulin and glucagon secretion

Static hormone secretion assays were performed on isolated pancreatic islets after overnight culture. Islets were pre-incubated in low-glucose KHB (2.8 mmol/l) for 45 min, followed by incubation in varying glucose concentrations (2.8, 5.6, 10, 20 mmol/l) for 1 h at 37°C. Supernatants were collected and stored at −20°C for insulin analysis. To assess glucagon secretion, islets previously exposed to 20 mmol/l glucose were transferred to 0.5 mmol/l glucose KHB for 1 h, and supernatants were collected. Islet insulin content was extracted using an ethanol/HCl solution. Analysis of supernatant insulin and glucagon levels was performed using HTRF insulin ultra-sensitive (ref.: 62IN2PEG, revvity) and HTRF glucagon (ref.: 62CGLPEG, revvity) kits.

### Dynamic insulin secretion

Dynamic insulin secretion was assessed using a custom-built perifusion chamber. Islets were pre-incubated in low-glucose KHB (2 mmol/l) and perifused at 1 ml/min with warmed buffer using a peristaltic pump. Effluent was collected every minute during baseline (2 mmol/l glucose) and after stimulation with 20 mmol/l glucose. Following 30 min, 30 mmol/l KCl was applied, and samples were collected every minute. Collected fractions were stored at −20°C. The insulin secretion levels were assessed using the HTRF insulin ultra-sensitive kit and normalised to DNA content using the Pico488 double-stranded DNA quantification kit (NBX-76675, Lumiprobe).

### Dispersion of pancreatic islets

Isolated pancreatic islets were dispersed by incubation with 0.05% trypsin–EDTA at 37°C for 2 min. Following enzymatic digestion, cells were washed, centrifuged and re-suspended in culture medium based on cell number. Dispersed cells were seeded onto coverslips and allowed to attach for 3–4 h before adding additional medium.

### Glucose uptake measurement

Glucose uptake into pancreatic islets was assessed using the Glucose Uptake-Glo assay kit (ref.: J1342, Promega). Groups of 5–20 islets were washed in glucose-free Flex medium (SILAC RPMI 1640 Flex Media, ref.: A2494201, Gibco) and imaged for size normalisation using a stereo microscope (Stereo microscope 305, Zeiss) with a camera (Axiocam 105 colour). Afterwards, pancreatic islets were incubated for 1 h (37°C and 5% CO_2_) in glucose-free Flex medium with 20 mmol/l 2-deoxy-d-glucose (2DG) and the standard protocol provided by the company was subsequently followed. See the ESM for further details.

### Western blot

Western blot analysis was performed on lysates from 300 pooled pancreatic islets per sample. After lysis and protein quantification, equal amounts of protein were separated by SDS-PAGE, transferred to PVDF membranes and probed with specific primary and HRP-conjugated secondary antibodies. Detection was carried out using enhanced chemiluminescence, and band intensities were quantified with Image Lab software (Bio-Rad, version 3.0.1), normalised to β-actin. *Taz*-KD samples were analysed in a paired design against matched WT controls, with WT values set to 100%. Variability due to differing culture durations is displayed in the ESM figures. The antibody list is described in ESM Table 2. See the ESM for further details.

### Hexokinases I–IV (glucokinase assay)

The fluorometric glucokinase activity assay kit (ab273303) was used to study hexokinase I–IV activity. If not stated otherwise, the protocol provided by the manufacturer was followed. Groups of 150 freshly isolated pancreatic islets were dispersed and homogenised. Background intensity was measured for each sample. Fluorescence intensity (excitation wavelength [λ_ex_]=540/20 nm, beam splitter [BS]=560 nm, emission wavelength [λ_em_]=590/20 nm) was measured using a Clariostar plate reader (BMG Labtech) and the obtained results were normalised by BCA protein assay. See the ESM for further details.

### G6PDH enzymatic assay

Activity of glucose 6-phosphate dehydrogenase (G6PDH) was assessed using a fluorometric kit from Abcam (ab176722). Groups of 50 pancreatic islets were dispersed and homogenised. The protocol was performed according to the guidelines of the manufacturer. Fluorescence intensity (λ_ex_=535/20 nm, BS=561 nm, λ_em_=587/20 nm) was measured using a Clariostar (BMG Labtech) plate reader and normalised to protein levels

### Measurement of the mitochondrial oxygen consumption rate and extracellular acidification rate

Mitochondrial respiration and glycolytic activity of whole pancreatic islets were assessed using a Seahorse XFe96 Analyzer. Sensor cartridges were prepared the day prior, and plates were coated with poly-l-lysine. Groups of 15 islets were seeded per well in Seahorse XF RPMI medium supplemented with 2.8 mmol/l glucose, glutamine and FBS. After equilibration, oxygen consumption rate (OCR) and extracellular acidification rate (ECAR) were measured using the Seahorse XF Cell Mito Stress Test, including sequential injections of glucose (2.8, 10 or 20 mmol/l), oligomycin, FCCP and antimycin A/rotenone.

Nutrient dependencies and capacities were evaluated using the Seahorse XF Mito Fuel Flex Test. Islets were sequentially treated with etomoxir, UK5099 and bis-2-(5-phenylacetamido-1,2,4-thiadiazol-2-yl)ethyl sulfide (BPTES) to block fatty acid, glucose and glutamine metabolism, respectively. Measurements were performed in replicates, and data were analysed using Wave (Agilent Technologies, version 2.6.3) and Prism software (Dotmatics, version 9.4). See the ESM for further details.

### ATP assay

ATP levels in pancreatic islets were measured using the CellTiter-Glo luminescent assay. Groups of five, ten or 20 islets were collected, imaged for size normalisation and processed according to the manufacturer’s protocol. Luminescence was recorded with a Clariostar plate reader and ATP concentrations were calculated using a standard curve (10 nmol/l to 10 µmol/l ATP).

### Mitochondrial membrane potential measurement

Mitochondrial membrane potential in whole pancreatic islets was assessed using tetramethylrhodamine methyl ester (TMRM) dye in quenching mode. Isolated islets were incubated with 200 nmol/l TMRM in low-glucose KHB (2 mmol/l glucose) for 45 min at 37°C. After washing, groups of 25 islets were transferred to a 96-well plate, and fluorescence was recorded using a plate reader. Following baseline measurements, islets were stimulated with 20 mmol/l glucose or 100 µmol/l tolbutamide. Carbonyl cyanide *m*-chlorophenyl hydrazone (CCCP) (25 µmol/l) was added at the end of each experiment as a depolarising control.

### Calcium measurements

Cytosolic, mitochondrial and endoplasmic reticulum (ER) calcium dynamics were assessed in pancreatic islets and dispersed islet cells using fluorescence-based imaging.

Cytosolic calcium levels were measured in whole and dispersed islets using the ratiometric dye Fura-2 acetoxymethyl ester (Fura-2AM). Islets were loaded with 5 µmol/l Fura-2AM, incubated for 2 h and then starved in low-glucose KHB. Calcium responses were recorded using an Axio Observer 7 microscope under basal (2 mmol/l glucose) and stimulated (20 mmol/l glucose) conditions, with 30 mmol/l KCl or tolbutamide (1–100 µmol/l).

Mitochondrial calcium levels were measured using the genetically encoded Mito-Pericam sensor. Dispersed islet cells were transduced with Mito-Pericam adenovirus and imaged 2–3 days later. Baseline measurements were performed at 2 mmol/l glucose, followed by stimulation with 20 mmol/l glucose and 30 mmol/l KCl.

ER calcium levels were assessed using the D4ER biosensor, specifically expressed in beta cells via a rat insulin promoter. Dispersed islet cells were transduced with D4ER adenovirus and imaged 2–3 days later. ER calcium dynamics were recorded under basal and high-glucose conditions, followed by thapsigargin treatment (3 µmol/l) to deplete ER calcium stores.

See the ESM for further details on calcium measurements.

### Redox histology

MiOxTaz mice (Mito-roGFP2-Orp1 × shTaz) were killed by ketamine/rompun injection. Redox histology was performed after cardiac perfusion and pancreas inflation with *N*-ethylmaleimide (NEM) to preserve thiol redox state [25, 28]. Pancreases were fixed in 4% PFA, cryoprotected in 30% sucrose, embedded in OCT and sectioned at 5 µm. Sections were stained for insulin and imaged for redox-sensitive green fluorescent protein 2–oxidant receptor peroxidase 1 (roGFP2-Orp1) fluorescence (emission 500–550 nm; excitation 405/470 nm), with islets identified by mCherry. Image analysis was performed in ImageJ using an automated script. Redox status was quantified as the 405/488 excitation ratio, normalised to WT. See the ESM for further details.

### H_2_O_2_ measurements

#### roGFP2-Orp1

After culture, islets were collected and washed in KHB containing 10 mmol/l glucose and 0.1% BSA for 10 min, and 20–25 islets per well were transferred to a U‐shaped 96‐well plate (TPP, ref.: 92097) in a total volume of 160 μl. roGFP2-Orp1 fluorescence (λ_ex1_=400/10 nm, λ_ex2_=482/16 nm, λ_em_=530/40 nm) was measured at 37°C and 5% CO_2_ for 19 h, using a Clariostar microplate reader (BMG Labtech).

#### HyPer7

Mitochondrial HyPer7 H_2_O_2_ sensor was used to test mitochondrial H_2_O_2_ levels in pancreatic islets. Adenoviral transduction was performed by adding 0.5 µl of adenovirus. At 2 days after transduction, cells were measured (λ_ex1_=405/20 nm, λ_ex2_=470/40 nm, BS=505 nm, λ_em_=550/100 nm) using an inverted epifluorescence microscope, Axio Observer 7, and a ×10 objective. Different glucose conditions (2 mmol/l and 20 mmol/l) were applied, followed by the addition of 25 μmol/l H_2_O_2_.

### NAD(P)H autofluorescence

The NADH/NADPH [NAD(P)H] levels of WT and *Taz*-KD pancreatic islets were measured in Clariostar plate reader experiments (λ_ex_=340/10 nm, BS=410 nm, λ_em_=450/10 nm) in parallel to other parameters (mitochondrial membrane potential and H_2_O_2_). In the H_2_O_2_ experiment, baseline NAD(P)H autofluorescence at 10 mmol/l glucose was monitored over time at 37°C, 18% O_2_ and 5% CO_2_. In the TMRM experiments, NAD(P)H autofluorescence was monitored in 2 mmol/l and 20 mmol/l glucose conditions and later normalised to CCCP levels.

### Confocal and stimulated emission depletion microscopy

Mitochondrial morphology in dispersed pancreatic islet cells was analysed using confocal and stimulated emission depletion (STED) microscopy. After isolation, islets were dispersed, seeded on coverslips and stained with 30 nmol/l MitoTracker Deep Red. Imaging was performed in 10 mmol/l glucose KHB using an Abberior Expert Line STED microscope with a ×100 silicon immersion objective.

Initial confocal scans provided an overview of mitochondrial labelling, followed by high-resolution confocal z-stacks (voxel size: 80×80×300 nm^3^) and STED imaging of selected regions (voxel size: 20×20×300 nm^3^). STED acquisition was optimised to minimise photobleaching using 775 nm depletion laser settings. Re-excitation signals were recorded and subtracted during post-processing.

Image deconvolution was performed using a Wiener filter and MATLAB routines. Further image processing, including noise reduction, background subtraction and sharpening, was conducted in ImageJ. Mitochondrial morphology parameters (surface area, volume, number, sphericity and bounding box dimensions) were quantified using Imaris software (Oxford Instruments, version 9.6.0). Mitochondria were categorised into three size classes based on surface area. Additionally, nearest neighbour analysis was performed to assess mitochondrial network connectivity. See the ESM for further details.

### MitoTracker for mitochondrial volume

Mitochondrial volume in dispersed pancreatic islet cells was assessed using MitoTracker Deep Red staining. Dispersed islet cells were incubated with 50 nmol/l MitoTracker in RPMI medium (0.1% FCS) for 30 min at 37°C, followed by washing and nuclear staining with 5 µg/ml Hoechst 33342. After washing, imaging was performed in KHB buffer. Mitochondrial volume was quantified by normalising MitoTracker fluorescence to Hoechst signal intensity.

### Citrate synthase assay

The colorimetric Citrate Synthase Assay Kit (ab239712) was used to determine the mitochondrial mass. A Clariostar plate reader was used to measure the absorbance (λ_ex_=412 nm). The citrate synthase activity was normalised by protein content.

### In vitro doxy model

To induce the *Taz*-KD in vitro after pancreatic islet isolation, doxy (doxycycline-hyclate D9891-5G, Merck/Sigma Aldrich) was dissolved in ddH_2_O and a final concentration of 1 µg/ml was added to the culturing medium (RPMI 1640, 21875034, Gibco, 10% FBS and 1% P/S). We found 1 µg/ml doxy to be optimal based on pancreatic islet function (cytosolic calcium levels) and *Taz* mRNA reduction. The culture medium was renewed every day. In vitro experiments were performed after 48 h and 7 days of doxy treatment. FGF-21 (Thermo Fisher catalogue no. 100-42-25UG, 50 nmol/l) was added to the cell culture when specified.

### RNA-seq

Total RNA was isolated from pancreatic islets, diluted in RNase-free water and quality-controlled using agarose gel electrophoresis, Bioanalyzer 2100 and Qubit 2.0 fluorometry. RNA samples were submitted to Novogene for sequencing.

mRNA was enriched using poly(A)-tail selection, fragmented and reverse-transcribed into cDNA. Library preparation included end-repair, adaptor ligation and PCR amplification with indexed primers. Final libraries were validated for concentration and fragment size and sequenced on the Illumina Novaseq X platform using paired-end 150 bp reads.

Gene expression levels were quantified as fragments per kb of transcript per million mapped reads (FPKM) values. Gene ontology (GO) enrichment analysis was performed to identify biological processes associated with differentially expressed genes (DEGs). The *Z* scores were calculated from FPKM data to compare gene expression across sample groups (WT and *Taz*-KD, in vivo and in vitro). A delta *Z* score (Δ*Z* score) was computed to quantify expression differences between in vivo and in vitro conditions. See the ESM for further details.

### Statistical analysis

GraphPad Prism software version 9.4 was used for statistical analysis. The presented values are shown in mean ± SEM. Details on the statistical analysis are in the figure legends. Mice were assigned to experimental groups based on their genotype (WT or transgenic), which was predetermined; therefore, no randomisation was performed. For FGF-21 treatment, islets were randomly allocated to treated or control groups within each genotype. For IHC experiments, group identities were anonymised using numbered labels.

## Results

### *Taz*-KD alters CL level and profile, and impacts levels of other phospholipids in pancreatic islets

**Fig. 1:**
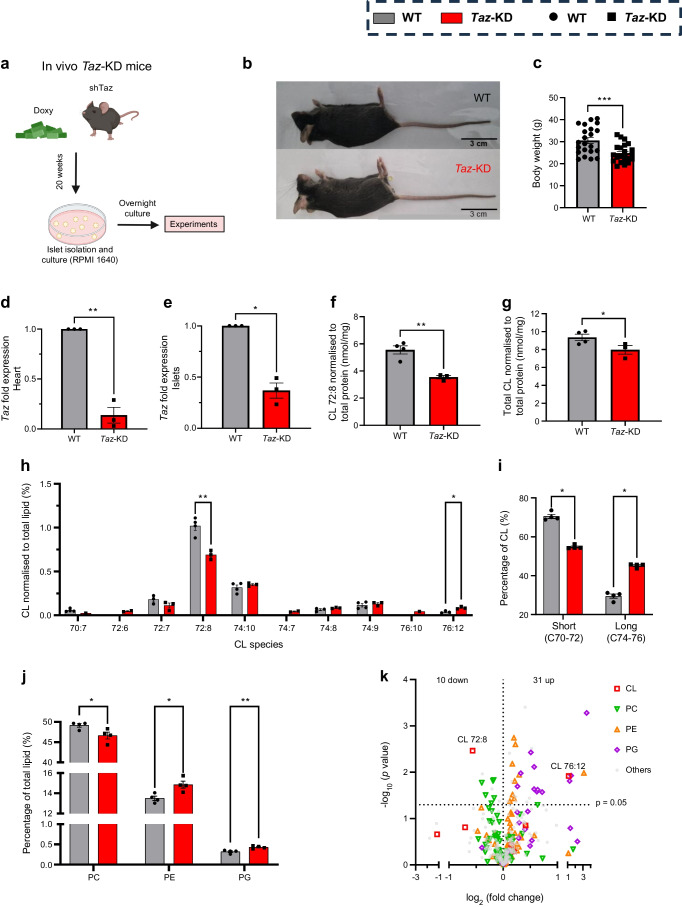
CL reduction and phospholipid alterations in *Taz*-KD pancreatic islets. (**a**) Schematic illustration of shTaz mouse model with 20 weeks of doxy feeding. Pancreatic islets are isolated and cultured in RPMI 1640 with 10% FBS and 1% P/S, and experiments are performed after overnight culture. Created with BioRender.com. (**b**) Representative image of a 20 wo *Taz*-KD mouse compared with a WT littermate. (**c**) Body weight of *Taz*-KD and WT at 20 wo, *N*=22. *Taz* gene expression in heart (**d**) and pancreatic islet (**e**) tissue. GAPDH was used as control, *N*=3. CL levels of the main species CL 72:8 (**f**) and total CL amount (**g**) of *Taz*-KD and WT pancreatic islets at 20 wo normalised to protein concentration. (**h**) CL species profile of *Taz*-KD and WT pancreatic islets at 20 wo normalised to total lipid amount, *N*=4 (WT), *N*=3 (*Taz*-KD); some replicates are below the limit of detection. (**i**) Quantification of the acyl chain length of all CL species (short: C70–C72; and long: C74–76), *N*=4. (**j**) Lipid concentration of the phospholipids PC, PE and PG of *Taz*-KD and WT pancreatic islets at 20 wo normalised to total lipid amount, *N*=4. The whole lipid class profile is presented in ESM Fig. 1e. (**k**) Volcano plot of all detected lipid species of *Taz*-KD and WT pancreatic islets at 20 wo. Statistical analysis showed that ten lipid species are significantly (*p*<0.05) downregulated and 31 are upregulated in *Taz*-KD. The lipid classes CL, PC, PE and PG are highlighted. All significantly altered lipid species are listed in ESM Fig. 1f. Data represent mean ± SEM (indicated by error bars); *N* numbers indicate number of animals; statistical significance was determined by unpaired Student’s *t* test: *p<0.05, **p<0.01, ***p<0.001

Male and female mice expressing an shRNA were used to attenuate *Taz* expression. *Taz*-KD mice and WT littermates were fed with doxy-containing chow to either induce *Taz*-KD or serve as a control (Fig. 1a, ESM Fig. 1a). *Taz*-KD mice were smaller and had decreased body weight at 20 weeks (Fig. 1b, c) and 50 weeks, but not at 10 weeks old (wo) (ESM Fig. 1b), compared with WT littermates. Efficient *Taz*-KD was confirmed in the heart and isolated pancreatic islets of 20 wo *Taz*-KD mice, with a more pronounced reduction in the heart (~90%) than in pancreatic islets (~64%) (Fig. 1d, e, ESM Fig. 1c, d).

To evaluate the effect of *Taz*-KD on the lipid composition of pancreatic islets, we performed lipidomic analysis on islets isolated from 20 wo *Taz*-KD and WT mice. As expected, levels of tetralinoleoyl cardiolipin (CL 72:8), the predominant CL species, as well as total CL content were significantly reduced in *Taz*-KD islets when normalised to total protein (Fig. 1f, g). In addition, the relative abundance of different CL species (normalised to total lipid amount) was altered in pancreatic islets from *Taz*-KD mice compared with WT littermates (Fig. 1h). We found a decrease in CL species with shorter acyl-chain lengths and an increase in CL species with longer acyl-chain lengths (Fig. 1i), indicating defective remodelling. Furthermore, the full lipid class profile of *Taz*-KD mice revealed a reduction of phosphatidylcholines (PCs) and an increase in phosphatidylethanolamines (PEs) and phosphatidylglycerols (PGs), which are precursors and/or involved in CL biosynthesis and remodelling [29] (Fig. 1j, ESM Fig. 1e). Overall, lipidomic analysis revealed a significant decrease in ten lipid species and an increase in 31 species in *Taz*-KD pancreatic islets, as illustrated in the volcano plot highlighting the most affected lipid classes (Fig. 1k, ESM Fig. 1f). The complete lipidomics data are found in an online depository (Mendeley 10.17632/gm7z58b95v.1).

In summary, knockdown of the transacylase Taz affects the lipidome of pancreatic islets, particularly altering the CL profile, reducing CL content and changing PC, PE and PG content.

### *Taz*-KD increases glucose uptake and diverts flux to glycolysis and hexosamine biosynthesis without affecting ATP levels

**Fig. 2:**
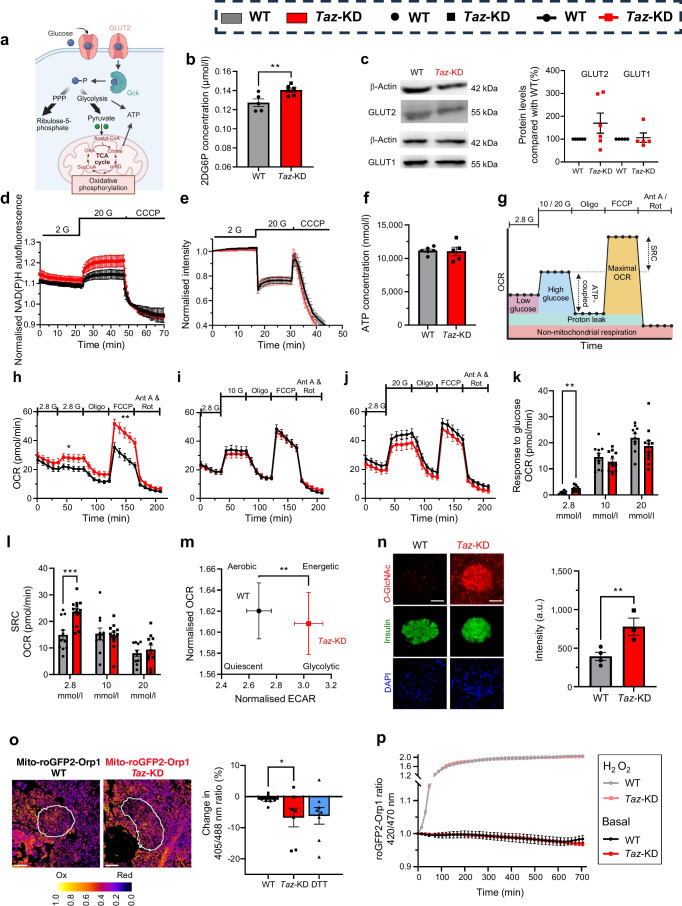
Increased glucose uptake and amplified metabolic parameters in *Taz*-KD pancreatic islets with no effects on ATP levels. (**a**) Schematic figure of glucose metabolism in pancreatic islet cells. Created with BioRender.com. (**b**) Glucose uptake of 20 wo WT and *Taz*-KD pancreatic islets, indicated by the levels of 2DG6P. *N*=5 (WT), *N*=6 (*Taz*-KD). (**c**) Representative western blot (left) and quantification (right) of GLUT2 normalised to β-actin in pancreatic islets of 20 wo *Taz*-KD mice, *N*=6. (**d**) NAD(P)H autofluorescence measurement of 20 wo WT and *Taz*-KD pancreatic islets in 2 mmol/l and 20 mmol/l glucose, normalised to CCCP, *N*=4 (WT), *N*=6 (*Taz*-KD). (**e**) Kinetic measurement of mitochondrial membrane potential of 20 wo WT and *Taz*-KD pancreatic islets using TMRM in quenching mode (200 nmol/l) in the presence of 2 and 20 mmol/l glucose. To uncouple the mitochondria, 25 µmol/l CCCP was added as a control at the end of the experiment, *N*=4. (**f**) ATP concentration of 20 wo WT and *Taz*-KD pancreatic islets using the CellTiter-Glo assay, *N*=6 (WT), *N*=5 (*Taz*-KD). (**g**) Schematic protocol of OCR and computable parameters during mitochondrial stress test using Seahorse. Created with BioRender.com. (**h**–**j**) OCR kinetics of 20 wo WT and *Taz*-KD pancreatic islets in response to (**h**) 2.8 mmol/l, (**i**) 10 mmol/l and (**j**) 20 mmol/l glucose stimulation followed by the addition of inhibitors of the respiratory chain complexes (Oligo, Ant A and Rot) and uncoupler (FCCP), *n*=11 (WT), *n*=13 (*Taz*-KD), *n* number of experiments include *N*=5 (WT) and *N*=4 (*Taz*-KD). (**k**, **l**) Quantification of response to glucose (**k**) and SRC (**l**) separated by glucose concentrations (2.8, 10 and 20 mmol/l), *n*=11 (WT), *n*=13 (*Taz*-KD), *n* number of experiments include *N*=5 (WT) and *N*=4 (*Taz*-KD). (**m**) Relationship between the normalised OCR and ECAR presented as an energy map in 20 mmol/l glucose. (**n**) Representative IHC images (left) and intensity quantification (right) of 20 wo WT and *Taz*-KD pancreatic islets stained against *O*-GlcNAc protein modification (red), insulin (green) and DAPI (blue), *N*=4; (WT), *N*=3 (*Taz*-KD) . Scale bar, 50 µm. (**o**) Representative ratiometric image (ImageJ Lookup table: ‘Fire’) of mito-roGFP2-Orp1/WT (left) and mito-roGFP2-Orp1/*Taz*-KD (right) pancreatic islets at 20 wo. Scale bar, 100 µm. Normalised percentage change in ratio of the redox state of the mito-roGFP2-Orp1 sensor in pancreatic islets of 20 wo mito-roGFP2-Orp1/WT and mito-roGFP2-Orp1/*Taz*-KD mice. DTT was used as a reductive control (blue). *N*=7 (WT), *N*=7 (*Taz*-KD), *N*=8 (DTT). Ox=oxidised, Red=reduced. (**p**) Ex vivo redox change in ratio of the redox state of the mito-roGFP2-Orp1 sensor in pancreatic islets of 20 wo mito-roGFP2-Orp1/WT and mito-roGFP2-Orp1/*Taz*-KD mice, *N*=4 (WT), *N*=4 (*Taz*-KD). Data represent mean ± SEM (indicated by error bars); *N* numbers indicate number of animals; statistical significance was determined by unpaired Student’s *t* test: *p<0.05, **p<0.01, ***p<0.001. Ant A, antimycin A; a.u., arbitrary units; 2DG6P, 2-deoxy-d-glucose 6-phosphate; G, glucose; α-KG, α-ketoglutarate; OAA, oxaloacetate; Oligo, oligomycin; Rot, rotenone; SucCoA, succinyl-CoA

To determine whether altered CL and lipid profiles affect *Taz*-KD islet function, we assessed key steps of glucose metabolism linked to insulin secretion (Fig. 2a). Pancreatic islets from 20 wo *Taz*-KD mice exhibited increased glucose uptake compared with WT (Fig. 2b), without changes in GLUT2 and GLUT1 protein levels (Fig. 2c, ESM Fig. 2a). Basal (ESM Fig. 2b) and glucose-stimulated NAD(P)H levels (Fig. 2d) were also elevated in *Taz*-KD islets, independent of hexokinases I–IV and G6PDH activity (ESM Fig. 2c, d). Despite increased glucose uptake and NAD(P)H levels, mitochondrial membrane potential and ATP production upon glucose stimulation were similar between *Taz*-KD and WT islets (Fig. 2e, f).

To evaluate mitochondrial function, we measured OCR and ECAR in isolated WT and *Taz*-KD pancreatic islets from 20 wo mice using a Seahorse XFe96 Analyzer (Fig. 2g). Under low glucose (2.8 mmol/l), *Taz*-KD islets showed increased basal OCR, greater glucose response and elevated spare respiratory capacity (SRC), proton leak and maximum respiration compared with WT (Fig. 2h–l, ESM Fig. 2e, f). These differences were not observed at 10 or 20 mmol/l glucose. ATP-coupled OCR remained unchanged (ESM Fig. 2g). Basal and non-mitochondrial respiration were similar between groups (ESM Fig. 2h, i).

ECAR measurements showed no significant differences under basal or glucose-stimulated conditions (fold-change) (ESM Fig. 2j). However, during the mitochondrial stress test (ESM Fig. 2k–m), ECAR was slightly elevated in *Taz*-KD islets following stimulation with 20 mmol/l glucose when normalised to baseline (ESM Fig. 2m). As previously reported in beta cells, oligomycin treatment reduced ECAR, probably due to low lactate dehydrogenase expression and minimal lactate production [30, 31]. To better resolve subtle shifts in metabolic phenotype, we generated an ‘energy map’ by plotting ECAR against OCR. This analysis revealed a distinct shift in *Taz*-KD islets towards a more glycolytic phenotype under high-glucose conditions (Fig. 2m), characterised by increased ECAR and unchanged or slightly reduced OCR.

To further explore downstream effects of increased glucose flux, we examined alternative metabolic routes. We observed enhanced protein *O*-GlcNAcylation in *Taz*-KD islets, as detected by IHC and western blot analysis (Fig. 2n, ESM Fig. 2n).

Fuel flexibility analysis (Mito Fuel Flex Test) showed no major differences in metabolic substrate dependency between WT and *Taz-*KD islets, aside from a slight increase in OCR following BPTES addition in *Taz*-KD islets (ESM Fig. 2o–q). ECAR measurements under these conditions were also unchanged (ESM Fig. 2r–s).

In summary, *Taz*-KD increases glucose uptake in pancreatic islets without altering ATP levels. Despite minimal changes in standard metabolic readouts, *Taz*-KD islets adopt a more glycolytic phenotype under high-glucose conditions and divert excess glucose into the hexosamine pathway, leading to increased protein *O*-GlcNAcylation.

### *Taz*-KD does not lead to in vivo H_2_O_2_ production in pancreatic islets

While increased ROS levels have been reported in tissues from individuals with BTHS and cell models [12], previous studies, including ours, showed unchanged ROS levels in *Taz*-KD mouse hearts due to enhanced antioxidant defences [9, 32]. Given that pancreatic islets have low antioxidant capacity [33] and ROS have been proposed as metabolic coupling factors for insulin secretion [34, 35], we investigated islet ROS levels in *Taz*-KD mice. To this end, we crossbred *Taz*-KD and WT mice with a transgenic line expressing the mitochondrial H_2_O_2_ sensor mito-roGFP2-Orp1 (ESM Fig. 2t). We confirmed sensor expression and functionality, showing dose-dependent oxidation by exogenous H_2_O_2_ (25–100 µmol/l) and full reduction with dithiothreitol (DTT) (ESM Fig. 2u). Using redox histology [25, 28], we analysed in vivo mitochondrial H_2_O_2_ levels in pancreatic islets from 20 and 50 wo *Taz*-KD mice and found a significant reduction in the mito-roGFP2-Orp1 sensor (Fig. 2o, ESM Fig. 2v), demonstrating a decreased mitochondrial redox state in pancreatic islets of *Taz*-KD mice. Ex vivo measurements of mito-roGFP2-Orp1 showed no differences between genotypes in either basal fluorescence or maximal response to exogenous H_2_O_2_ (Fig. 2p). Analysis of different redox proteins showed that while peroxiredoxin 3 (Prx3) protein levels were marginally increased in *Taz*-KD mice, catalase levels were decreased (ESM Fig. 2w, x). Glutathione peroxidase 4 (GPX4), NADPH oxidase 4 (NOX4) and nuclear factor erythroid 2-related factor 2 (NRF2) protein levels were unchanged among the genotypes (ESM Fig. 2w, x). Finally, we evaluated key proteins involved in the integrated stress response (ISR) and found no differences in ATF4, total and phosphorylated eIF2α, or growth differentiation factor 15 (GDF-15) protein levels in islets from 20 wo *Taz*-KD mice (ESM Fig. 2y, z).

In summary, *Taz*-KD pancreatic islets did not exhibit excessive ROS production but rather displayed a reduced mitochondrial redox state with an increased abundance of Prx3.

### *Taz*-KD enhances cytosolic calcium without early secretory defects but increases age-related glucotoxic vulnerability

**Fig. 3:**
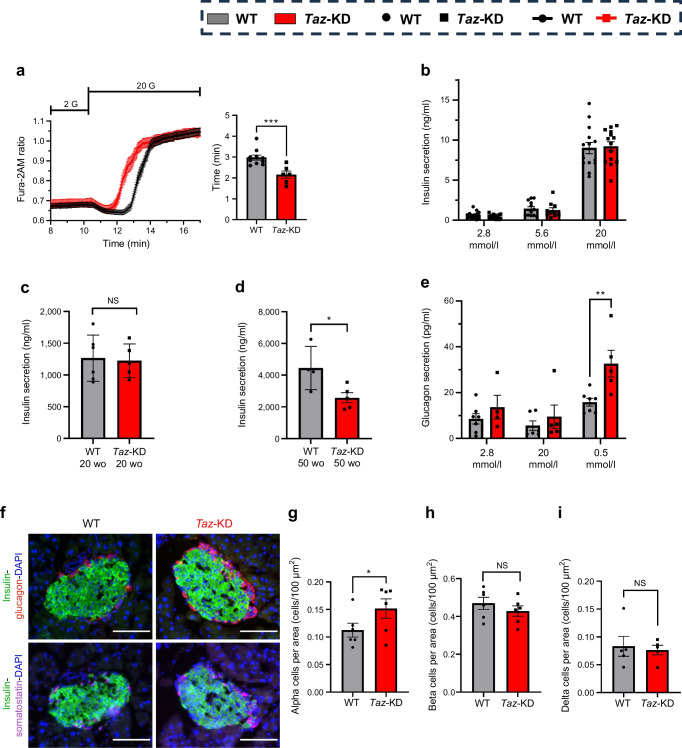
Faster cytosolic calcium mobilisation and increased glucagon secretion in *Taz*-KD pancreatic islets. (**a**) Left, cytosolic calcium levels of 20 wo WT and *Taz*-KD pancreatic islets in 2 mmol/l and 20 mmol/l glucose concentration, monitored by Fura-2AM ratio (340/380 nm). Right, quantification of the inflection point, reflecting the timing of cytosolic calcium mobilisation upon glucose stimulation (20 mmol/l), *N*=10 (WT), *N*=6 (*Taz*-KD). (**b**) Quantification of static ex vivo GSIS of 20 wo WT and *Taz*-KD pancreatic islets at 2.8, 5.6 and 20 mmol/l glucose concentrations, *N*=16 (WT), *N*=17 (*Taz*-KD). (**c**, **d**) Insulin secreted in the medium from (**c**) 20 or (**d**) 50 wo WT and *Taz*-KD islets upon 20 mmol/l glucose. 20 wo *N*=6 (WT), *N*=5 (*Taz*-KD), 50 wo *N*=4 (WT), *N*=5 (*Taz*-KD). (**e**) Glucagon secretion of 20 wo WT and *Taz*-KD pancreatic islets. *N*=7 (2.8 mmol/l, WT), *N*=6 (20 mmol/l, WT), *N*=7 (0.5 mmol/l, WT), *N*=4 (2.8 mmol/l, *Taz*-KD), *N*=5 (20 and 0.5 mmol/l, *Taz*-KD). (**f**) Representative images of 20 wo WT (left) and *Taz*-KD (right) pancreas cryoslices, with IHC showing pancreatic islets stained against insulin (green) and glucagon (red) (top panel) or insulin (green) and somatostatin (magenta) (bottom panel) together with DAPI (blue). The glucagon–insulin and somatostatin–insulin double stainings for each genotype represent the same pancreatic islet at a different cutting depth. Scale bar, 100 µm. (**g**–**i**) Quantitative ImageJ analysis of alpha (**g**), beta (**h**) and delta (**i**) cell number of IHC on cryoslices and counting DAPI spots of WT and *Taz*-KD pancreatic islets at 20 wo normalised to pancreatic islet area, *N*=5. Data represent mean ± SEM (indicated by error bars); *N* and *n* numbers indicate number of animals and experiments, respectively; statistical significance was determined by unpaired Student’s *t* test or two-way ANOVA for glucagon secretion: *p<0.05, **p<0.01, ***p<0.001. G, glucose

To investigate the downstream effects of increased glucose uptake in *Taz*-KD islets, we assessed key parameters of beta cell function. We monitored cytosolic calcium dynamics using the ratiometric indicator Fura-2AM. Interestingly, although we observed similar cytosolic calcium levels in *Taz*-KD and WT pancreatic islets under both low- and high-glucose conditions, *Taz*-KD pancreatic islets displayed a faster calcium influx upon glucose stimulation (Fig. 3a, ESM Fig. 3a). The calcium handling inside other organelles plays a crucial role in regulating the cytosolic calcium concentration upon stimulation with glucose [36–38]. Therefore, we dispersed WT and *Taz*-KD pancreatic islets and used adenoviral vectors to express ER- and mitochondrial matrix-targeted calcium sensors to investigate calcium handling in these cellular compartments (ESM Fig. 3b). The response of mitochondrial (ESM Fig. 3c) and ER calcium (ESM Fig. 3d) to glucose stimulation was similar in *Taz*-KD and WT pancreatic islet cells. Finally, we investigated the cytosolic calcium response in dispersed pancreatic islet cells of WT and *Taz*-KD. Interestingly, the accelerated cytosolic calcium influx/mobilisation observed in intact *Taz*-KD pancreatic islets was lost following islet dispersion, with no difference detected between dispersed *Taz*-KD and WT islet cells (ESM Fig. 3e). This finding highlights the critical role of beta–beta and alpha–beta cell crosstalk in mediating the observed phenotype [39–41].

We then tested secretory function upon different glucose concentrations in isolated pancreatic islets from 20 wo WT and *Taz*-KD mice. Analysis of static glucose-stimulated insulin secretion (GSIS) revealed no difference in insulin secretion (Fig. 3b) and insulin content at a range of different glucose concentrations (ESM Fig. 3f). Moreover, we performed a dynamic insulin secretion analysis to investigate the different phases of insulin secretion. We observed no difference in first- and second-phase insulin secretion dynamics between WT and *Taz*-KD pancreatic islets (ESM Fig. 3g, h), either following glucose stimulation or after islet depolarisation.

Finally, islets from 20 and 50 wo WT and *Taz*-KD mice were cultured for 19 h under glucotoxic conditions (20 mmol/l glucose). In islets from 20 wo mice, insulin secretion was comparable between genotypes (Fig. 3c). However, in 50 wo mice, *Taz*-KD islets displayed reduced accumulated insulin in the culture medium compared with WT controls (Fig. 3d).

In summary, *Taz*-KD in islets accelerates glucose-stimulated calcium influx while maintaining insulin secretion at younger ages. However, under prolonged high-glucose exposure insulin secretion is impaired in aged (50 wo) *Taz*-KD islets, suggesting an increased vulnerability to glucotoxic stress over time.

### *Taz*-KD increases pancreatic islet glucagon secretion and alpha cell number

We next asked whether *Taz*-KD might lead to changes in glucagon secretion. Interestingly, we observed increased glucagon secretion in *Taz*-KD pancreatic islets (Fig. 3e) and no significant differences in islet glucagon content (ESM Fig. 3i). To investigate the origins of altered glucagon secretion in *Taz*-KD mice, we analysed pancreatic islet cell composition (Fig. 3f–i, ESM Fig. 3j). *Taz*-KD islets showed an increased number of glucagon-positive alpha cells (Fig. 3f, g), with no changes in insulin-positive beta cells (Fig. 3f, h) or somatostatin-positive delta cells (Fig. 3f, i). This difference in alpha cells was not observed at 10 weeks (ESM Fig. 3k). In addition, pancreatic and duodenal homeobox 1 (PDX1) expression was unchanged between WT and *Taz*-KD islets at 20 weeks (ESM Fig. 3l). Proliferation (Ki67, ESM Fig. 3m) and apoptosis (cleaved caspase-3, ESM Fig. 3n) markers in alpha and beta cells were also similar in both groups.

In summary, *Taz*-KD pancreatic islets displayed increased alpha cell number with higher glucagon secretion, which was not associated with changes in proliferation (Ki67) or apoptosis (cleaved caspase-3) rate.

### Increased mitochondrial volume in pancreatic islets of *Taz*-KD

**Fig. 4:**
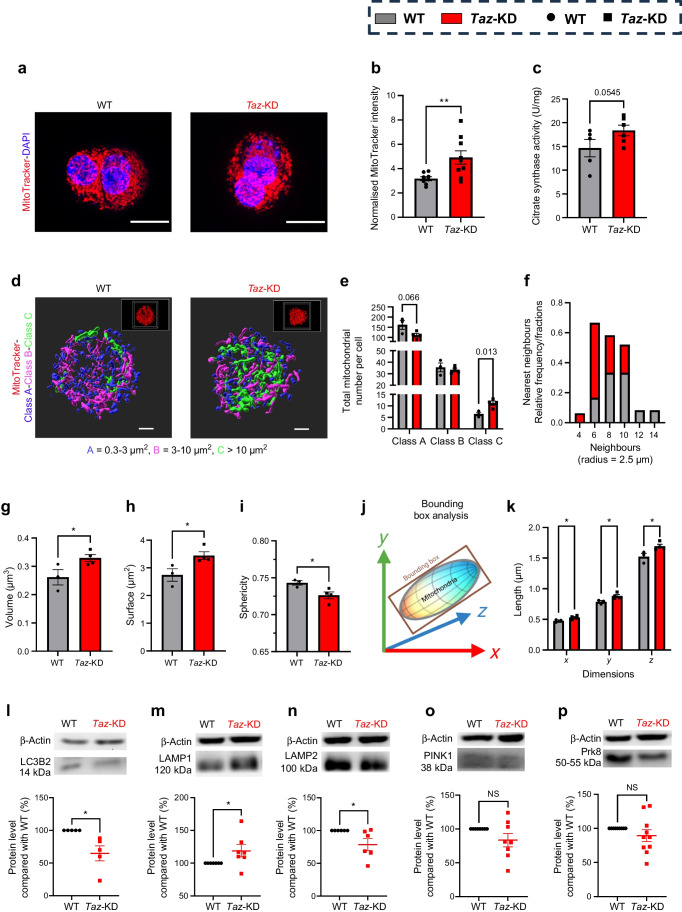
Increased single mitochondrial dimensions lead to overall increase in mitochondrial volume but decrease in number. (**a**, **b**) Representative images of dispersed pancreatic islet cell cluster of 20 wo WT and *Taz*-KD mice and mitochondrial volume quantification (**b**) using confocal microscopy with MitoTracker Deep Red and DAPI. Scale bar, 10 µm. *N*=8 (WT), *N*=9 (*Taz*-KD). (**c**) Citrate synthase activity assay of 20 wo WT and *Taz*-KD pancreatic islets to determine mitochondrial mass normalised to protein content, *N*=5 (WT), *N*=6 (*Taz*-KD). (**d**) Representative 3D rendering images of the mitochondrial network from single dispersed pancreatic islet cells of 20 wo WT and *Taz*-KD mice, classified according to their surface area into three different classes: Class A: 0.3–3 µm^2^ (blue); Class B: 3–10 µm^2^ (magenta); Class C >10 µm^2^ (green). The respective confocal microscopy image is shown in the upper right. Scale bar, 2 µm. (**e**,** f**) Mitochondrial density analysis (**e**), including the mitochondrial number per pancreatic islet cell in separate classes (Class A, Class B and Class C), and (**f**) a frequency distribution histogram using a nearest neighbour analysis testing for neighbouring mitochondria in a radius of 2.5 µm from WT and *Taz*-KD dispersed pancreatic islet cells. The frequency distribution histogram displays the fractions of WT and *Taz*-KD mitochondria that have a certain number of neighbours. *N*=3 (WT), *N*=4 (*Taz*-KD). (**g**–**i**) Single mitochondrion analysis of volume (**g**), surface area (**h**) and sphericity (**i**) from 20 wo WT and *Taz*-KD dispersed pancreatic islet cells, *N*=3 (WT), *N*=4 (*Taz*-KD). (**j**) Schematic figure of object-oriented bounding box analysis in 3D (*x*, *y* and *z*). (**k**) Quantification of bounding box in single mitochondria from 20 wo WT and *Taz*-KD dispersed pancreatic islet cells. (**l**–**p**) Representative western blot and quantification of LC3B2 (**l**), LAMP1 (**m**), LAMP2 (**n**), PINK1 (**o**) and Prk8 (**p**) normalised to β-actin in pancreatic islets of 20 wo *Taz*-KD mice, *N*=5 (LC3B2), *N*=7 (LAMP1), *N*=6 (LAMP2), *N*=8 (PINK1), *N*=10 (Prk8). Data represent mean ± SEM (indicated by error bars); *N* numbers indicate number of animals; statistical significance was determined by unpaired or paired (western blot) Student’s *t* test: *p<0.05, **p<0.01

*Taz*-KD has been associated with impaired mitophagy in various tissues [8, 42, 43]. To assess mitochondrial dynamics in *Taz*-KD pancreatic islets, we analysed mitochondrial volume and morphology. Confocal microscopy revealed increased MitoTracker intensity in *Taz*-KD islet cells, indicating increased mitochondrial volume (Fig. 4a, b), supported by elevated citrate synthase activity (Fig. 4c).

STED microscopy was used to study the mitochondrial network morphology of pancreatic islet cells in greater detail. STED microscopy resolves mitochondrial ultrastructure with a greater detail and sharpness, allowing for a more accurate determination of mitochondrial volume. As expected, STED microscopy reported a 38.75 ± 3.86% decrease in total mitochondrial volume and a 30.69 ± 11.24% increase in mitochondrial number compared with that suggested by confocal microscopy (ESM Fig. 4a–c). After imaging and 3D rendering, we classified mitochondria into three different categories based on surface area: small (Class A): 0.3–3 µm^2^; medium (Class B): 3–10 µm^2^; and large (Class C): >10 µm^2^. We observed an increase in large (Class C) and a decrease in small (Class A) mitochondria in *Taz*-KD pancreatic islet cells (Fig. 4d, e). A nearest neighbour analysis showed a decreased frequency of mitochondrial neighbours in *Taz*-KD islet cells (Fig. 4f), which is consistent with the decrease in overall mitochondrial number due to a more connected mitochondrial network in *Taz*-KD (ESM Fig. 4d). Individual mitochondria of *Taz*-KD displayed increased volume and surface area but decreased sphericity (Fig. 4g–i) due to the shift towards bigger mitochondria, as the single mitochondrion morphology in separated classes remained unchanged (ESM Fig. 4e). A bounding box analysis confirmed the enlargement of *Taz*-KD mitochondria, which is not directed, but instead similar on each axis (*x*, *y* and *z*) (Fig. 4j, k, ESM Fig. 4f).

Western blot analysis showed decreased microtubule-associated protein 1 light chain 3 beta (LC3B2 [membrane-bound] and LC3B1 [cytosolic]) protein levels in *Taz*-KD islets, with an unchanged LC3B2/LC3B1 ratio (Fig. 4l, ESM Fig. 4g, h). Lysosomal-associated membrane protein 1 (LAMP1) was increased and lysosomal-associated membrane protein 2 (LAMP2) decreased in *Taz*-KD (Fig. 4m, n, ESM Fig. 4j). Autophagy-related 7 (ATG7) (ESM Fig. 4i), PTEN-induced putative kinase 1 (PINK1) and parkin (Prk8) levels were unchanged (Fig. 4o, p, ESM Fig. 4j). No differences were observed in mitofusin 1/2 protein expression (ESM Fig. 4k, l).

Together, these data indicate that *Taz*-KD islets display altered mitochondrial morphology and increased volume, accompanied by changes in some autophagy-related proteins.

### In vivo *Taz*-KD leads to improved whole-body glucose tolerance, increased glucose uptake with preserved plasma insulin and glucagon secretion, and elevated levels of plasma FGF-21

**Fig. 5:**
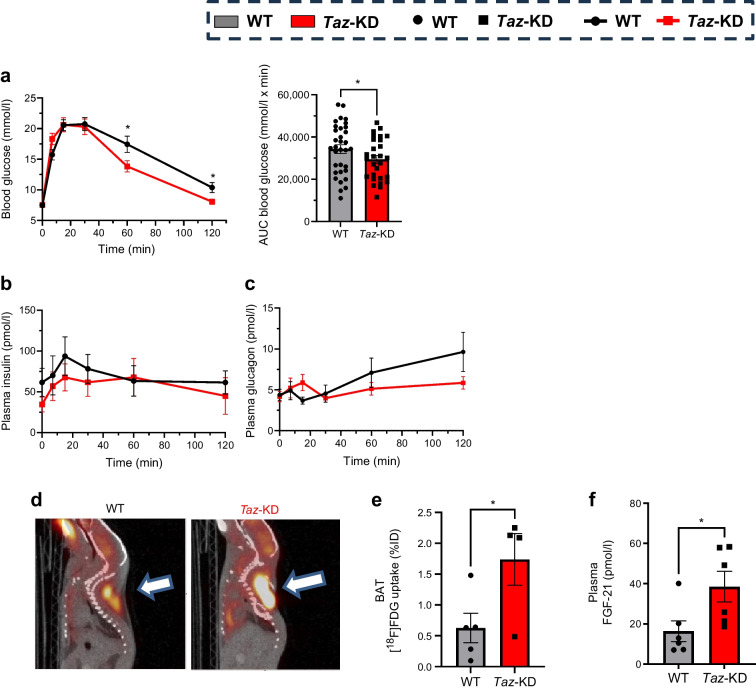
Improved glucose tolerance in GTT results from increased peripheral glucose uptake. (**a**) Blood glucose levels (left) and quantified AUC (right) of *Taz*-KD and WT mice at 20 wo during i.p. GTT, *N*=24 (WT), *N*=22 (*Taz*-KD). (**b**, **c**) Plasma insulin (**b**) and plasma glucagon (**c**) normalised to body weight of 20 wo WT and *Taz*-KD mice during i.p. GTT, *N*=7 (WT) and *N*=8 (*Taz*-KD). (**d**) Representative PET/CT images of BAT [^18^F]FDG uptake in WT and *Taz*-KD mice at 20 wo, presented as percentage of injection dose (%ID). (**e**) Quantification of BAT [^18^F]FDG uptake normalised to lung uptake in WT and *Taz*-KD mice, *N*=5 (WT), *N*=4 (*Taz*-KD). (**f**) Plasma levels of FGF-21 in WT and *Taz*-KD mice at 20 wo, *N*=6. Data represent mean ± SEM (indicated by error bars); *N* numbers indicate number of animals; statistical significance was determined by unpaired Student’s *t* test: *p<0.05

To assess the impact of *Taz*-KD on whole-body glucose homeostasis, i.p. GTTs were performed. *Taz*-KD mice showed significantly lower blood glucose levels at 60 and 120 min compared with WT at both 20 and 50 weeks (Fig. 5a, ESM Fig. 5a). Plasma insulin and glucagon levels were overall comparable between *Taz*-KD and WT mice during the GTT (Fig. 5b, c, ESM Fig. 5b, c), indicating preserved hormone secretion. However, plasma glucagon levels in *Taz*-KD mice displayed an altered profile, peaking at 15 min post injection, whereas WT mice showed a decline (Fig. 5c, ESM Fig. 5c). These data suggest improved glucose tolerance in *Taz*-KD mice, probably due to accelerated glucose clearance [19].

To further investigate glucose clearance, glucose uptake in BAT was assessed by [^18^F]FDG PET/CT imaging. *Taz*-KD mice exhibited significantly higher glucose uptake in BAT at 20 weeks (Fig. 5d, e, ESM Fig. 5d). Since FGF-21 is a key regulator of glucose uptake and energy metabolism and was found to be elevated in individuals with BTHS [44], we next measured its circulating levels. Plasma FGF-21 levels were elevated in *Taz*-KD mice at 12 and 20 weeks, but not at 50 weeks (Fig. 5f, ESM Fig. 5e). FGF-21 gene expression was increased in heart and skeletal muscle, with no changes in liver and pancreatic islets (ESM Fig. 5f).

In summary, *Taz*-KD mice displayed enhanced glucose tolerance, increased BAT glucose uptake, preserved insulin and glucagon secretion, and elevated plasma FGF-21 levels. These adaptations occurred despite decreased islet CL levels and lipid profile alterations, indicating compensatory changes in islet cell composition, mitochondrial morphology and metabolism that preserved islet function.

### In vitro *Taz*-KD leads to loss of pancreatic islet function

**Fig. 6:**
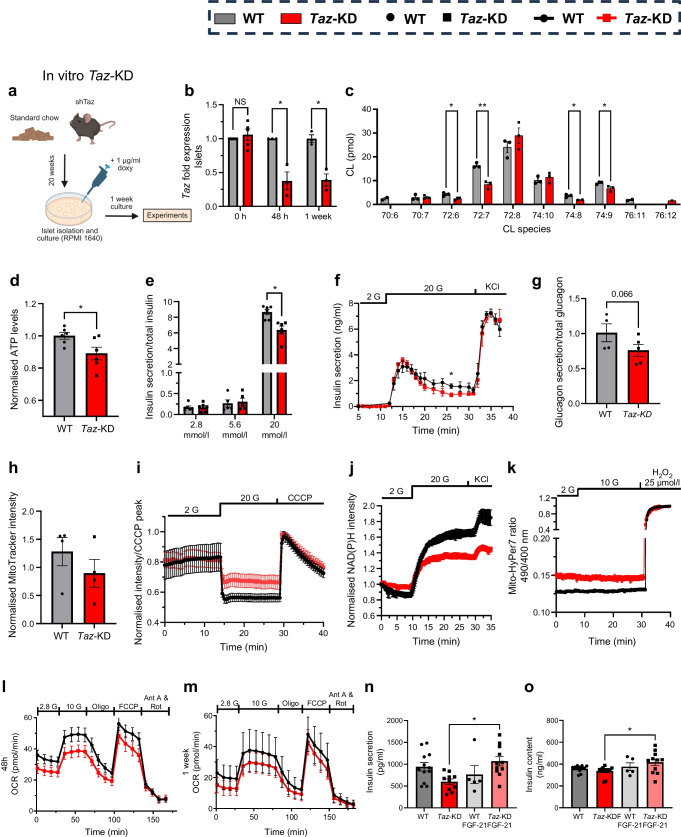
In vitro, *Taz*-KD leads to impaired beta cell function. (**a**) Schematic illustration of *Taz*-KD in vitro model: WT (control littermates from shTaz crossing with C57Bl6N) and shTaz mice are fed with control chow. After islet isolation, islets are treated with 1 µg/ml doxy in culture. Created with BioRender.com. (**b**) Fold expression levels of *Taz* gene in WT and *Taz*-KD pancreatic islets of in vitro model after 0 h, 48 h and 1 week in doxy culture, *N*=5 (0 h), *N*=3 (48 h, 1 week). (**c**) CL species profile of *Taz*-KD and WT pancreatic islets after 1 week of doxy incubation, *N*=3; some replicates are below the limit of detection. (**d**) ATP levels of WT and *Taz*-KD pancreatic islets after 1 week of doxy incubation using the CellTiter-Glo assay and normalised to islet size, *N*=6. (**e**) Quantification of static GSIS of WT and *Taz*-KD pancreatic islets after 1 week of doxy incubation in 2.8, 5.6 and 20 mmol/l glucose, *N*=6. (**f**) Dynamic GSIS of WT and *Taz*-KD pancreatic islets after 1 week of doxy incubation, following the insulin levels at 2 mmol/l glucose, 20 mmol/l glucose and 30 mmol/l KCl conditions, *N*=3. (**g**) Glucagon secretion of WT and *Taz*-KD pancreatic islets after 1 week of doxy incubation in 0.5 mmol/l glucose, *N*=4 (WT), *N*=5 (*Taz*-KD). (**h**) Quantification of mitochondrial volume of WT and *Taz*-KD pancreatic islets after 1 week of doxy incubation using MitoTracker Deep Red and DAPI, *N*=4. (**i**) Mitochondrial membrane potential via TMRM, (**j**) NAD(P)H levels via autofluorescence and (**k**) H_2_O_2_ levels (normalised to 25 μmol/l H_2_O_2_) via Mito-HyPer7 sensor of WT and *Taz*-KD pancreatic islets after 1 week of doxy incubation, *N*=3. OCR kinetics after 48 h (**l**) and 1 week (**m**) in *Taz*-KD pancreatic islets in response to 10 mmol/l glucose stimulation followed by the addition of inhibitors of the respiratory chain complexes (Oligo, Ant A and Rot) and uncoupler (FCCP), *N*=4 (48 h), *N*=3 (1 week). (**n**) Insulin secretion in 20 mmol/l glucose in WT and *Taz*-KD pancreatic islets after 1 week of doxy incubation with or without 50 nmol/l FGF-21, *n*=5–12 wells with five islets each, *N*=4 animals (**o**) Insulin content was measured at the end of the insulin secretion assay. *n*=5–12 wells with five islets each, *N*=4 animals. Data represent mean ± SEM (indicated by error bars); *N* numbers indicate number of animals, *n* indicates number of experiments. Statistical significance was determined by unpaired Student's *t* test or (**e**, **n**, **o**) one-way ANOVA followed by Tukey’s multiple comparison: *p<0.05, **p<0.01. Ant A, antimycin A; G, glucose; Oligo, oligomycin; Rot, rotenone

To determine whether the effects observed in vivo were adaptive, we used an in vitro approach to assess the direct impact of *Taz*-KD in pancreatic islets. Islets from *Taz*-shRNA and WT mice (fed a control diet) were cultured with doxy to induce *Taz*-KD (Fig. 6a). *Taz* expression was reduced by ~62% (after 48 h and 1 week) (Fig. 6b, ESM Fig. 6a), similar to in vivo levels. In contrast to in vivo findings, no changes in alpha, beta or delta cell composition were observed after 1 week of doxy treatment (ESM Fig. 6b, c). Lipidomic analysis confirmed reduced CL species (72:6, 72:7, 74:8, 74:9) in *Taz*-KD islets (Fig. 6c, ESM Fig. 6d).

Functionally, in vitro *Taz*-KD reduced ATP levels (Fig. 6d) without affecting calcium influx (ESM Fig. 6e). GSIS (20 mmol/l) was impaired after 1 week, primarily during the second phase of secretion (Fig. 6e, f), while glucagon secretion remained unchanged (Fig. 6g). Mitochondrial mass (Fig. 6h) and* O*-linked β-*N*-acetylglucosamine (*O*-GlcNAc) levels (ESM Fig. 6f) were similar between groups.

Metabolic analysis showed decreased mitochondrial membrane potential (Fig. 6i, ESM Fig. 6g) and NAD(P)H levels (Fig. 6j, ESM Fig. 6h) upon glucose administration after 1 week of *Taz*-KD in vitro. Mitochondrial H_2_O_2_ production was increased after 48 h and 1 week of in vitro *Taz*-KD (Fig. 6k, ESM Fig. 6i, j). Basal OCR was decreased after 48 h, with no significant changes after 1 week of *Taz*-KD (Fig. 6l, m, ESM Fig. 6k, l).

To test for potential protective effects of circulating factors, islets were co-cultured with doxy and FGF-21 for 1 week. FGF-21 treatment restored GSIS in *Taz*-KD islets (Fig. 6n) and slightly increased islet insulin content (Fig. 6o), without affecting glucose uptake (ESM Fig. 6n). Insulin secretion remained elevated after normalisation to insulin content (ESM Fig. 6m), suggesting FGF-21 enhances beta cell secretory capacity.

In conclusion, in vitro *Taz*-KD impairs mitochondrial metabolism and insulin secretion, while FGF-21 treatment rescues beta cell function.

### In vivo and in vitro transcriptomics show differently regulated pathways

**Fig. 7:**
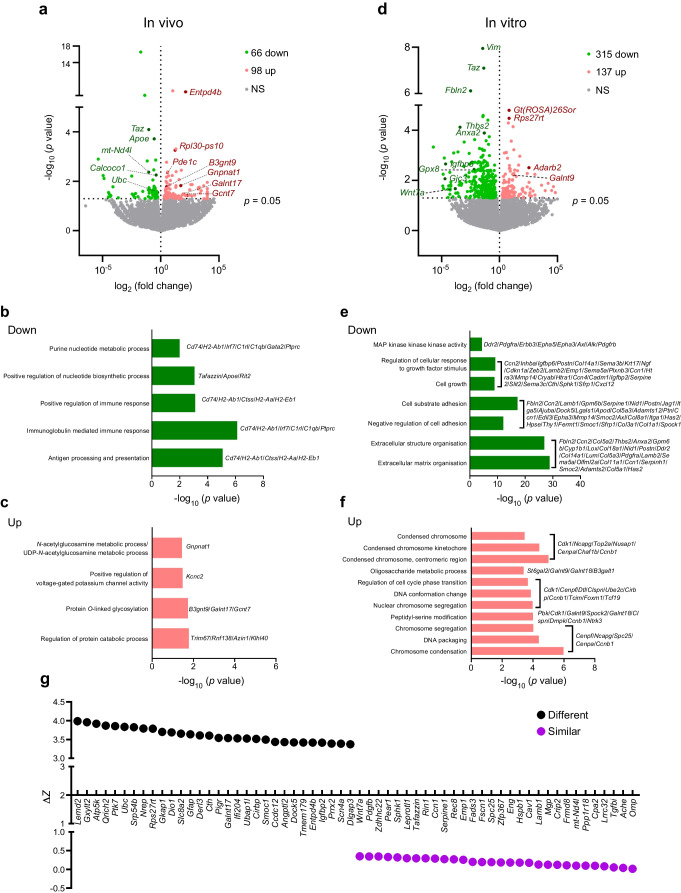
Transcriptomics reveals differently regulated pathways of in vivo and in vitro *Taz*-KD. (**a**) Bulk mRNA-seq analysis of *Taz*-KD and WT pancreatic islets using the in vivo model. Volcano plot illustrates the upregulation of 98 genes and downregulation of 66 genes (*p*<0.05) with a selection of highlighted genes. Identified relevant GO pathways with genes that are downregulated (**b**) or upregulated (**c**) in *Taz*-KD. *N*=3. (**d**) Bulk mRNA-seq analysis of *Taz*-KD and WT pancreatic islets using the in vitro model with 1 week of doxy incubation. Volcano plot illustrates the upregulation of 137 genes and downregulation of 315 genes (*p*<0.05) with a selection of highlighted genes. Identified relevant GO pathways with genes that are downregulated (**e**) or upregulated (**f**) in *Taz*-KD. *N*=3. (**g**) Comparison of DEGs from in vivo and in vitro *Taz*-KD. The Δ*Z* score represents the deviation of gene expression of *Taz*-KD in vivo and *Taz*-KD in vitro. Δ*Z* score=0 reflects the same gene expression in in vivo and in vitro *Taz*-KD. High Δ*Z* score reflects a high variation in gene expression in in vivo and in vitro *Taz*-KD. *N* numbers indicate number of animals

To explore mechanisms underlying adaptive (in vivo) vs deleterious (in vitro) responses to *Taz*-KD, we performed bulk mRNA-seq of pancreatic islets*.* In vivo, 66 genes were downregulated and 98 upregulated in *Taz*-KD islets compared with WT (Fig. 7a). Downregulated genes included *ApoE*, *Taz* and *mt-ND4l* (purine nucleotide metabolic and biosynthetic processes) and immune regulators *Cd74*, *H2-Ab1* and *H2-Aa* (Fig. 7b). Upregulated genes were associated with *N*-acetyl-glucosamine metabolism (*Gnpnat1*) and *O*-linked glycosylation (*B3gnt9*, *Galnt17*, *Gcnt7*) (Fig. 7c).

In vitro, 315 genes were downregulated and 137 upregulated after 1 week of doxy-induced *Taz*-KD (Fig. 7d). Downregulated genes included *Pdgfra*, *Fbln2* and *Col1a1* (MAPK activity, cell adhesion, extracellular matrix [ECM] organisation) (Fig. 7e), while upregulated genes were related to chromosome segregation and cell cycle regulation (Fig. 7f). Ten DEGs overlapped between both models, including downregulation of *Taz*, *Wnt7a* and *Leprotl1*, while degradation-related genes *Ubc* and *Derl3* showed opposing regulation (ESM Fig. 7a).

Comparison of *Z* scores highlighted genes most similarly or differentially regulated between models (Fig. 7g). Differentially regulated genes included those involved in* O*-linked glycosylation (*Galnt17*, *Gxylt2*, *Entpd4b*), catabolic pathways (*Ubc*, *Derl3*, *Qrich2*), ribosomal proteins (*Rps27rt*, *Cirbp*), ion transport (*Slc8a2*, *Smoc1*, *Scn4a*) and antioxidant/one-carbon metabolism (*Cth*). Similarly regulated genes involved cell fate (*Wnt7a*, *Emp1*, *Spc25*), signalling (*Leprotl1*, *Serpine1*, *Pear1*), protein binding (*Spc25*, *Rec8*, *Crip2*, *Frmd8*), growth factors (*Pdgfb*, *Tgfbi*) and ECM (*Lamb1*, *Ccn1*).

In summary, in vivo *Taz*-KD led to downregulation of genes related to catabolic processes, autophagy and protein modification, suggesting adaptive mechanisms. In contrast, in vitro knockdown induced MAPK signalling, ECM downregulation and cell cycle activation, probably contributing to islet dysfunction.

## Discussion

BTHS is caused by mutations in the *TAZ* gene that impair CL remodelling and classically manifests with cardiomyopathy. However, individuals with BTHS also experience endocrine and metabolic disturbances such as recurrent hypoglycaemia and delayed growth [45]. Despite CL importance in cellular metabolism, its impact on pancreatic islets has been largely overlooked [46, 47]. To date, only a single study has examined pancreatic islet dysfunction in the context of Taz deficiency [23]. Our work addresses this gap of knowledge, revealing how pancreatic islet function is maintained in a mouse model of BTHS with global *Taz*-KD and the consequences for whole-body glucose metabolism.

Although our findings complement previous work, notable differences exist between our study and that of Cole et al [23]. Discrepancies between the studies may originate from variations in diet composition, which affect doxy consumption and overall metabolic status. Furthermore, Cole et al employed C57BL/6J mice, which lack nicotinamide nucleotide transhydrogenase (NNT) among other differences [48, 49], whereas our study used C57BL/6N mice, known for more robust insulin secretion, redox homeostasis and mitochondrial function [50].

*Taz* gene expression in pancreatic islets in our model is reduced by approximately 64%, while *Taz*-KD in the heart reaches ~90% with a clear heart failure phenotype, as we have shown previously [9]. Although the effect in islets is milder, we believe it mimics the clinical scenario observed in human carriers of *Taz* loss-of-function mutations, who show mild or no endocrine symptoms, while still recapitulating the cardiac dysfunction characteristic of BTHS. Notably, certain human missense mutations, especially those outside the HX4D domain, also result in milder BTHS manifestations [51]. In addition, similar degrees of knockdown in vitro are sufficient to trigger defects in insulin secretion, suggesting that the long-term in vivo environment permits adaptive processes that are not apparent in short-term in vitro studies.

The in vivo environment probably includes the influence of circulating factors. Consistent with findings in humans [44], our data demonstrate that *Taz*-KD in mice leads to elevated plasma FGF-21 levels. FGF-21 is a key metabolic regulator involved in lipid oxidation, glucose homeostasis and mitochondrial function [52, 53]. Alongside increased FGF-21, we observed improved glucose tolerance, which, as shown in this study, is not driven by increased plasma insulin levels but instead probably reflects enhanced insulin sensitivity in peripheral tissues such as BAT (this study), the heart, skeletal muscle and lymphocytes [10, 15].

The mechanisms underlying metabolic substrate utilisation and adaptive responses to meet energy demands in BTHS remain poorly understood. As we did not observe clear activation of the ISR in islets of 20 wo *Taz*-KD mice, despite evidence of ISR activation in the heart [15], we propose that mitochondrial inefficiency may trigger transient or tissue-specific stress responses. These early adaptations probably contribute to the systemic metabolic remodelling observed in *Taz*-KD mice, including elevated FGF-21 levels and increased glucose uptake across multiple tissues. The precise molecular mediators linking mitochondrial dysfunction to these systemic responses remain unclear but may include upregulation of glucose transporters in target tissues, an increased insulin-to-glucagon ratio and other circulating factors such as GDF-15, which has also been shown to be elevated in individuals with BTHS [44]. Interestingly, we show that FGF-21 is able to rescue insulin secretion after *Taz*-KD in vitro. In diabetic model mice, FGF-21 was shown to improve insulin secretion by activating extracellular signal-regulated kinase 1/2 and Akt signalling pathways [54]. Furthermore, FGF-21 has been shown to mitigate oxidative stress in cardiomyocytes [53, 55, 56] and could also play a role in the protective effect observed in vitro, as we observed that H_2_O_2_ levels are increased after 48 h and 1 week of *Taz*-KD*.*

Notably, hypermetabolism and increased FGF-21 are frequently associated with mitochondrial disorders [57, 58] and recent studies have shown that FGF-21 can modulate mitochondrial bioenergetics and dynamics by inhibiting mitochondrial fission and oxidative stress [53, 59]. Furthermore, an increase in mitochondrial volume has been recognised as an effective strategy to sustain respiration, reduce ROS accumulation and promote cell survival under conditions of stress and proliferation [58, 60, 61].

*Taz*-KD resulted in altered islet cell composition, characterised by an increased alpha cell number and elevated glucagon secretion. The underlying cause of alpha cell expansion remains unclear. However, similar increases in alpha cell mass have been reported in mouse models of mitochondrial dysfunction with reduced complex I expression [62]. Whether this reflects enhanced neogenesis or beta to alpha cell transdifferentiation is unknown, as we detected no significant changes in proliferation or cell death markers. Notably, beta to alpha cell transdifferentiation has been described in models of diabetes and insulin resistance [63], suggesting it may contribute in this context. Increased alpha cell numbers were also reported in the only other study investigating pancreatic islets in *Taz*-KD mice, although that study described reduced glucagon content [23].

The impact of lipid composition on alpha cell function remains unclear. In individuals with BTHS, cardiac metabolic shifts from fatty acid oxidation to glycolysis elevate circulating NEFAs [10]. It is plausible that increased NEFAs contribute to the altered glucagon phenotype observed in *Taz*-KD mice. Recent studies suggest that alpha cells depend on fatty acid oxidation for ATP production under low-glucose conditions [64]; thus, elevated NEFAs and hypoglycaemia may enhance alpha cell activity and glucagon secretion.

Additionally, increased hepatic glycogenolysis and elevated blood glucose during exercise have been reported in *Taz*-KD mice [11], potentially driven by higher glucagon levels. Beyond glycaemic control, glucagon promotes lipolysis and may contribute to the lean phenotype of *Taz*-KD mice, as well as stimulate hepatic FGF-21 secretion [65]. It is therefore tempting to hypothesise that elevated glucagon secretion significantly shapes the metabolic profile of BTHS. Moreover, chronically increased glucagon may impact cardiac function [66, 67] and pancreatic islet physiology through paracrine signalling [40, 68–70]. Increased alpha cell number might also be responsible for some of the metabolic effects observed, such as increased glucose uptake and increased respiration. Altogether, these findings highlight the need to investigate alpha cell mass and glucagon regulation in individuals with BTHS to better understand their contribution to disease progression.

Excessive mitochondrial fusion or fission has been implicated in pancreatic beta cell gain or loss of function [71, 72]. CL serves as a signalling platform and was shown to be important for proper mitochondrial function and dynamics (reviewed in [73]). Here, we show for the first time in pancreatic islets that CL remodelling is important for regulation of mitochondrial mass, as demonstrated by increased mitochondrial volume in *Taz*-KD islets. Consistent with previous studies in other BTHS cell types [8, 43], we observed that *Taz*-KD alters the expression of autophagy and mitophagy markers, which may contribute to changes in mitochondrial network dynamics. Under conditions of mitochondrial stress or damage, CL can be externalised to the outer mitochondrial membrane, serving as a signal for the recruitment of mitophagy-related proteins including LC3B2, a key component of autophagosome formation. However, the functional relevance of these changes should be interpreted with caution, as we did not assess autophagic flux, which is essential for definitive conclusions. Additionally, mitochondrial morphology is influenced by various lipid species, including PG and PC, both of which were found to be elevated in *Taz*-KD islets. Moreover, recently *Taz*-KD has been suggested to affect mitochondria–ER contact sites [74], which may further contribute to alterations in the mitochondrial lipid profile [75].

Our findings reveal increased *O*-GlcNAcylation in *Taz*-KD islets, a dynamic, reversible post-translational modification essential for cellular signalling, metabolism and mitochondrial quality control, including mitophagy [76]. Alongside phosphorylation, *O*-GlcNAcylation is a key regulatory mechanism, particularly relevant in beta cells due to their high *O*-GlcNAc transferase expression and sensitivity to nutrient flux. It has been linked to beta cell adaptation in prediabetic states [77] and modulation of autophagy, as shown in diabetic hearts where elevated *O*-GlcNAcylation promotes mitochondrial elongation [78, 79]. In beta cells, increased *O*-GlcNAcylation has been reported to preserve insulin secretion during hyperglycaemia, possibly through enhanced *Ins1*/*Ins2* gene expression via epigenetic regulation [80]. Further studies are needed to clarify its specific targets and functional role in *Taz*-KD islets.

Among the limitations of our study, we acknowledge that while the use of intact islets preserves physiological cell–cell interactions, it limits cell-type-specific resolution. Altered islet composition in *Taz*-KD mice (particularly increased alpha cell numbers) may influence metabolic readouts. Future studies employing beta cell-specific *Taz*-KD models combined with cell-type-resolved metabolic flux analyses will be essential to disentangle these contributions.

In summary, our study demonstrates that pancreatic islet function is largely preserved in a mouse model of BTHS, despite marked cellular, morphological, transcriptional and metabolic adaptations. These adaptations probably represent compensatory mechanisms in response to impaired CL remodelling. Our findings provide new insights into how defective CL remodelling affects islet biology and systemic metabolism. Notably, we show that plasma FGF-21 levels and glucagon secretion are elevated in *Taz*-KD mice, suggesting a broader metabolic impact of BTHS beyond cardiac dysfunction. As therapeutic advances improve life expectancy in individuals with BTHS, understanding the secondary metabolic consequences in non-cardiac tissues becomes increasingly relevant. Given the central role of pancreatic islets in energy homeostasis, our work highlights the importance of CL remodelling in islet function and offers new perspectives for the study of metabolic diseases such as diabetes.

## Supplementary Information

Below is the link to the electronic supplementary material.

ESM (PDF 4617 KB)

## Data Availability

The complete gene list of RNA-seq data and lipidomics profile information (including lipid class, species, double bond, hydroxylation, carbon length and fatty acid profiles) are in online deposit: https://data.mendeley.com/datasets/gm7z58b95v/1. All other data will be made available upon request.

## Abbreviations

λ_em_: Emission wavelength
λ_ex_: Excitation wavelength
Δ*Z* score: Delta *Z* score
ATF4: Activating transcription factor 4
BAT: Brown adipose tissue
BPTES: Bis-2-(5-phenylacetamido-1,2,4-thiadiazol-2-yl)ethyl sulfide
BS: Beam splitter
BTHS: Barth syndrome
CCCP: Carbonyl cyanide *m*-chlorophenyl hydrazone
CL: Cardiolipin
CT: Computed tomography
DEG: Differentially expressed gene
2DG: 2-Deoxy-d-glucose
Doxy: Doxycycline
DTT: Dithiothreitol
ECAR: Extracellular acidification rate
ECM: Extracellular matrix
eIF2α: Eukaryotic initiation factor 2 alpha subunit
ER: Endoplasmic reticulum
[^18^F]FDG: [^18^F]Fluorodeoxyglucose
FGF-21: Fibroblast growth factor 21
FPKM: Fragments per kb of transcript per million mapped reads
Fura-2AM: Fura-2 acetoxymethyl ester
GDF-15: Growth differentiation factor 15
*O*-GlcNAc: *O*-linked β-*N*-acetylglucosamine
GO: Gene ontology
G6PDH: Glucose 6-phosphate dehydrogenase
GSIS: Glucose-stimulated insulin secretion
HTRF: Homogeneous time-resolved fluorescence
IHC: Immunohistochemistry
ISR: Integrated stress response
KHB: Krebs–Henseleit buffer
LAMP1: Lysosomal-associated membrane protein 1
LAMP2: Lysosomal-associated membrane protein 2
LC3B1: Microtubule-associated protein 1 light chain 3 beta (cytosolic)
LC3B2: Microtubule-associated protein 1 light chain 3 beta (membrane-bound)
Mito-roGFP2-Orp1: Mitochondria-redox-sensitive green fluorescent protein 2–oxidant receptor peroxidase 1
NAD(P)H: NADH/NADPH
OCR: Oxygen consumption rate
OCT: Optimal cutting temperature
PC: Phosphatidylcholine
PE: Phosphatidylethanolamine
PET: Positron emission tomography
PFA: Paraformaldehyde
PG: Phosphatidylglycerol
PINK1: PTEN-induced putative kinase 1
Prk8: Parkin
Prx3: Peroxiredoxin 3
P/S: Penicillin/streptomycin
roGFP2-Orp1: Redox-sensitive green fluorescent protein 2–oxidant receptor peroxidase 1
ROS: Reactive oxygen species
shRNA: Short hairpin RNA
SRC: Spare respiratory capacity
STED: Stimulated emission depletion
Taz: Tafazzin
*Taz*-KD: *Taz* knockdown
TMRM: Tetramethylrhodamine methyl ester
wo: Week(s) old
WT: Wild-type

## References

[1] Dudek J, Maack C (2017) Barth syndrome cardiomyopathy. Cardiovasc Res 113(4):399–410. 10.1093/cvr/cvx014 28158532 10.1093/cvr/cvx014

[2] Sandlers Y, Mercier K, Pathmasiri W et al (2016) Metabolomics reveals new mechanisms for pathogenesis in barth syndrome and introduces novel roles for cardiolipin in cellular function. PLoS One 11(3):e0151802. 10.1371/journal.pone.0151802 27015085 10.1371/journal.pone.0151802 PMC4807847

[3] Duncan AL (2020) Monolysocardiolipin (MLCL) interactions with mitochondrial membrane proteins. Biochem Soc Trans 48(3):993–1004. 10.1042/BST20190932 32453413 10.1042/BST20190932 PMC7329354

[4] Corey RA, Harrison N, Stansfeld PJ, Sansom MSP, Duncan AL (2022) Cardiolipin, and not monolysocardiolipin, preferentially binds to the interface of complexes III and IV. Chem Sci 13(45):13489–13498. 10.1039/d2sc04072g 36507170 10.1039/d2sc04072g PMC9682889

[5] Thompson R, Jefferies J, Wang S et al (2022) Current and future treatment approaches for Barth syndrome. J Inherit Metab Dis 45(1):17–28. 10.1002/jimd.12453 34713454 10.1002/jimd.12453

[6] Liang Z, Schmidtke MW, Greenberg ML (2022) Current knowledge on the role of cardiolipin remodeling in the context of lipid oxidation and Barth syndrome. Front Mol Biosci 9:915301. 10.3389/fmolb.2022.915301 35693555 10.3389/fmolb.2022.915301 PMC9184736

[7] Dudek J, Maack C (2022) Mechano-energetic aspects of Barth syndrome. J Inherit Metab Dis 45(1):82–98. 10.1002/jimd.12427 34423473 10.1002/jimd.12427

[8] Zhang J, Liu X, Nie J, Shi Y (2022) Restoration of mitophagy ameliorates cardiomyopathy in Barth syndrome. Autophagy 18(9):2134–2149. 10.1080/15548627.2021.2020979 34985382 10.1080/15548627.2021.2020979 PMC9466615

[9] Bertero E, Nickel A, Kohlhaas M et al (2021) Loss of mitochondrial Ca(2+) uniporter limits inotropic reserve and provides trigger and substrate for arrhythmias in Barth syndrome cardiomyopathy. Circulation 144(21):1694–1713. 10.1161/CIRCULATIONAHA.121.053755 34648376 10.1161/CIRCULATIONAHA.121.053755

[10] Chowdhury A, Boshnakovska A, Aich A et al (2023) Metabolic switch from fatty acid oxidation to glycolysis in knock-in mouse model of Barth syndrome. EMBO Mol Med 15(9):e17399. 10.15252/emmm.202317399 37533404 10.15252/emmm.202317399 PMC10493589

[11] Schweitzer GG, Ditzenberger GL, Hughey CC et al (2023) Elevated liver glycogenolysis mediates higher blood glucose during acute exercise in Barth syndrome. PLoS One 18(8):e0290832. 10.1371/journal.pone.0290832 37651450 10.1371/journal.pone.0290832 PMC10470866

[12] Petit PX, Ardilla-Osorio H, Penalvia L, Rainey NE (2020) Tafazzin Mutation Affecting Cardiolipin Leads to Increased Mitochondrial Superoxide Anions and Mitophagy Inhibition in Barth Syndrome. Cells 9(10):2333. 10.3390/cells9102333 33096711 10.3390/cells9102333 PMC7589545

[13] Patil VA, Li Y, Ji J, Greenberg ML (2020) Loss of the mitochondrial lipid cardiolipin leads to decreased glutathione synthesis. Biochim Biophys Acta Mol Cell Biol Lipids 2:158542. 10.1016/j.bbalip.2019.158542 10.1016/j.bbalip.2019.158542 PMC6980711 31672571

[14] He Q, Harris N, Ren J, Han X (2014) Mitochondria-targeted antioxidant prevents cardiac dysfunction induced by tafazzin gene knockdown in cardiac myocytes. Oxid Med Cell Longev 2014:654198. 10.1155/2014/654198 25247053 10.1155/2014/654198 PMC4160652

[15] Kutschka I, Bertero E, Wasmus C et al (2023) Activation of the integrated stress response rewires cardiac metabolism in Barth syndrome. Basic Res Cardiol 118(1):47. 10.1007/s00395-023-01017-x 37930434 10.1007/s00395-023-01017-x PMC10628049

[16] Han X, Yang J, Cheng H, Yang K, Abendschein DR, Gross RW (2005) Shotgun lipidomics identifies cardiolipin depletion in diabetic myocardium linking altered substrate utilization with mitochondrial dysfunction. Biochemistry 44(50):16684–16694. 10.1021/bi051908a 16342958 10.1021/bi051908a

[17] Han X, Yang J, Yang K, Zhao Z, Abendschein DR, Gross RW (2007) Alterations in myocardial cardiolipin content and composition occur at the very earliest stages of diabetes: a shotgun lipidomics study. Biochemistry 46(21):6417–6428. 10.1021/bi7004015 17487985 10.1021/bi7004015 PMC2139909

[18] Sustarsic EG, Ma T, Lynes MD et al (2018) Cardiolipin synthesis in brown and beige fat mitochondria is essential for systemic energy homeostasis. Cell Metab 28(1):159–174 e111. 10.1016/j.cmet.2018.05.003 29861389 10.1016/j.cmet.2018.05.003 PMC6038052

[19] Cole LK, Mejia EM, Vandel M et al (2016) Impaired cardiolipin biosynthesis prevents hepatic steatosis and diet-induced obesity. Diabetes 65(11):3289–3300. 10.2337/db16-0114 27495222 10.2337/db16-0114 PMC5079636

[20] Raja V, Reynolds CA, Greenberg ML (2017) Barth syndrome: a life-threatening disorder caused by abnormal cardiolipin remodeling. J Rare Dis Res Treat 2(2):58–62. 10.29245/2572-9411/2017/2.1087 31032491 10.29245/2572-9411/2017/2.1087 PMC6482962

[21] Donati MA, Malvagia S, Pasquini E et al (2006) Barth syndrome presenting with acute metabolic decompensation in the neonatal period. J Inherit Metab Dis 29(5):684. 10.1007/s10545-006-0388-7 16906470 10.1007/s10545-006-0388-7

[22] Cade WT, Spencer CT, Reeds DN et al (2013) Substrate metabolism during basal and hyperinsulinemic conditions in adolescents and young-adults with Barth syndrome. J Inherit Metab Dis 36(1):91–101. 10.1007/s10545-012-9486-x 22580961 10.1007/s10545-012-9486-x PMC3608431

[23] Cole LK, Agarwal P, Doucette CA et al (2021) Tafazzin deficiency reduces basal insulin secretion and mitochondrial function in pancreatic islets from male mice. Endocrinology 162(7):bqab102. 10.1210/endocr/bqab102 34019639 10.1210/endocr/bqab102 PMC8197286

[24] Acehan D, Vaz F, Houtkooper RH et al (2011) Cardiac and skeletal muscle defects in a mouse model of human Barth syndrome. J Biol Chem 286(2):899–908. 10.1074/jbc.M110.171439 21068380 10.1074/jbc.M110.171439 PMC3020775

[25] Fujikawa Y, Roma LP, Sobotta MC et al (2016) Mouse redox histology using genetically encoded probes. Sci Signal 9(419):rs1. 10.1126/scisignal.aad3895 26980443 10.1126/scisignal.aad3895

[26] Cui YF, Ma M, Wang GY, Han DE, Vollmar B, Menger MD (2005) Prevention of core cell damage in isolated islets of Langerhans by low temperature preconditioning. World J Gastroenterol 11(4):545–550. 10.3748/wjg.v11.i4.545 15641143 10.3748/wjg.v11.i4.545 PMC4250808

[27] Roma LP, Pascal SM, Duprez J, Jonas JC (2012) Mitochondrial oxidative stress contributes differently to rat pancreatic islet cell apoptosis and insulin secretory defects after prolonged culture in a low non-stimulating glucose concentration. Diabetologia 55(8):2226–2237. 10.1007/s00125-012-2581-6 22643931 10.1007/s00125-012-2581-6

[28] Lin H, Suzuki K, Smith N et al (2024) A role and mechanism for redox sensing by SENP1 in β-cell responses to high fat feeding. Nat Commun 15(1):334. 10.1038/s41467-023-44589-x 38184650 10.1038/s41467-023-44589-x PMC10771529

[29] Vianello E, Ambrogi F, Kalousova M et al (2024) Circulating perturbation of phosphatidylcholine (PC) and phosphatidylethanolamine (PE) is associated to cardiac remodeling and NLRP3 inflammasome in cardiovascular patients with insulin resistance risk. Exp Mol Pathol 137:104895. 10.1016/j.yexmp.2024.104895 38703553 10.1016/j.yexmp.2024.104895

[30] Schuit F, Van Lommel L, Granvik M et al (2012) β-cell-specific gene repression: a mechanism to protect against inappropriate or maladjusted insulin secretion? Diabetes 61(5):969–975. 10.2337/db11-1564 22517647 10.2337/db11-1564 PMC3331770

[31] Sekine N, Cirulli V, Regazzi R et al (1994) Low lactate dehydrogenase and high mitochondrial glycerol phosphate dehydrogenase in pancreatic beta-cells. Potential role in nutrient sensing. J Biol Chem 269(7):4895–4902 8106462

[32] Goncalves RLS, Schlame M, Bartelt A, Brand MD, Hotamisligil GS (2021) Cardiolipin deficiency in Barth syndrome is not associated with increased superoxide/H(2) O(2) production in heart and skeletal muscle mitochondria. FEBS Lett 595(3):415–432. 10.1002/1873-3468.13973 33112430 10.1002/1873-3468.13973 PMC7894513

[33] Lenzen S (2017) Chemistry and biology of reactive species with special reference to the antioxidative defence status in pancreatic β-cells. Biochim Biophys Acta Gen Subj 1861(8):1929–1942. 10.1016/j.bbagen.2017.05.013 28527893 10.1016/j.bbagen.2017.05.013

[34] Pi J, Bai Y, Zhang Q et al (2007) Reactive oxygen species as a signal in glucose-stimulated insulin secretion. Diabetes 56(7):1783–1791. 10.2337/db06-1601 17400930 10.2337/db06-1601

[35] Plecita-Hlavata L, Jaburek M, Holendova B et al (2020) Glucose-stimulated insulin secretion fundamentally requires H(2)O(2) signaling by NADPH oxidase 4. Diabetes 69(7):1341–1354. 10.2337/db19-1130 32245800 10.2337/db19-1130

[36] Raffaello A, Mammucari C, Gherardi G, Rizzuto R (2016) Calcium at the center of cell signaling: interplay between endoplasmic reticulum, mitochondria, and lysosomes. Trends Biochem Sci 41(12):1035–1049. 10.1016/j.tibs.2016.09.001 27692849 10.1016/j.tibs.2016.09.001 PMC5123979

[37] Gilon P, Chae HY, Rutter GA, Ravier MA (2014) Calcium signaling in pancreatic β-cells in health and in Type 2 diabetes. Cell Calcium 56(5):340–361. 10.1016/j.ceca.2014.09.001 25239387 10.1016/j.ceca.2014.09.001

[38] Ravier MA, Daro D, Roma LP et al (2011) Mechanisms of control of the free Ca2+ concentration in the endoplasmic reticulum of mouse pancreatic β-cells: interplay with cell metabolism and [Ca2+]c and role of SERCA2b and SERCA3. Diabetes 60(10):2533–2545. 10.2337/db10-1543 21885870 10.2337/db10-1543 PMC3178295

[39] Ashcroft FM, Proks P, Smith PA, Ammala C, Bokvist K, Rorsman P (1994) Stimulus-secretion coupling in pancreatic β cells. J Cell Biochem 55(Suppl):54–65. 10.1002/jcb.240550007 7929618 10.1002/jcb.240550007

[40] Moede T, Leibiger IB, Berggren PO (2020) Alpha cell regulation of beta cell function. Diabetologia 63(10):2064–2075. 10.1007/s00125-020-05196-3 32894317 10.1007/s00125-020-05196-3 PMC7476996

[41] Serre-Beinier V, Bosco D, Zulianello L et al (2009) Cx36 makes channels coupling human pancreatic β-cells, and correlates with insulin expression. Hum Mol Genet 18(3):428–439. 10.1093/hmg/ddn370 19000992 10.1093/hmg/ddn370 PMC2638800

[42] Hsu P, Liu X, Zhang J, Wang HG, Ye JM, Shi Y (2015) Cardiolipin remodeling by TAZ/tafazzin is selectively required for the initiation of mitophagy. Autophagy 11(4):643–652. 10.1080/15548627.2015.1023984 25919711 10.1080/15548627.2015.1023984 PMC4502692

[43] Russo S, De Rasmo D, Rossi R, Signorile A, Lobasso S (2024) SS-31 treatment ameliorates cardiac mitochondrial morphology and defective mitophagy in a murine model of Barth syndrome. Sci Rep 14(1):13655. 10.1038/s41598-024-64368-y 38871974 10.1038/s41598-024-64368-y PMC11176169

[44] Liu O, Chinni BK, Manlhiot C, Vernon HJ (2023) FGF21 and GDF15 are elevated in Barth Syndrome and are correlated to important clinical measures. Mol Genet Metab 140(3):107676. 10.1016/j.ymgme.2023.107676 37549445 10.1016/j.ymgme.2023.107676

[45] Clarke SL, Bowron A, Gonzalez IL et al (2013) Barth syndrome. Orphanet J Rare Dis 8:23. 10.1186/1750-1172-8-23 23398819 10.1186/1750-1172-8-23 PMC3583704

[46] Li J, Du H, Zhang M et al (2019) Amorphous solid dispersion of Berberine mitigates apoptosis via iPLA(2)β/Cardiolipin/Opa1 pathway in db/db mice and in Palmitate-treated MIN6 β-cells. Int J Biol Sci 15(7):1533–1545. 10.7150/ijbs.32020 31337982 10.7150/ijbs.32020 PMC6643135

[47] Song H, Wohltmann M, Tan M, Ladenson JH, Turk J (2014) Group VIA phospholipase A2 mitigates palmitate-induced β-cell mitochondrial injury and apoptosis. J Biol Chem 289(20):14194–14210. 10.1074/jbc.M114.561910 24648512 10.1074/jbc.M114.561910 PMC4022886

[48] Nemoto S, Kubota T, Ohno H (2022) Metabolic differences and differentially expressed genes between C57BL/6J and C57BL/6N mice substrains. PLoS One 17(12):e0271651. 10.1371/journal.pone.0271651 36548271 10.1371/journal.pone.0271651 PMC9778930

[49] Close AF, Chae H, Jonas JC (2021) The lack of functional nicotinamide nucleotide transhydrogenase only moderately contributes to the impairment of glucose tolerance and glucose-stimulated insulin secretion in C57BL/6J vs C57BL/6N mice. Diabetologia 64(11):2550–2561. 10.1007/s00125-021-05548-7 34448880 10.1007/s00125-021-05548-7

[50] Santos LRB, Muller C, de Souza AH et al (2017) NNT reverse mode of operation mediates glucose control of mitochondrial NADPH and glutathione redox state in mouse pancreatic β-cells. Mol Metab 6(6):535–547. 10.1016/j.molmet.2017.04.004 28580284 10.1016/j.molmet.2017.04.004 PMC5444111

[51] Sakamoto O, Kitoh T, Ohura T, Ohya N, Iinuma K (2002) Novel missense mutation (R94S) in the TAZ (G4.5) gene in a Japanese patient with Barth syndrome. J Hum Genet 47(5):229–231. 10.1007/s100380200030 12032589 10.1007/s100380200030

[52] Geng L, Lam KSL, Xu A (2020) The therapeutic potential of FGF21 in metabolic diseases: from bench to clinic. Nat Rev Endocrinol 16(11):654–667. 10.1038/s41574-020-0386-0 32764725 10.1038/s41574-020-0386-0

[53] Zhang K, Gan J, Wang B et al (2025) FGF21 protects against HFpEF by improving cardiac mitochondrial bioenergetics in mice. Nat Commun 16(1):1661. 10.1038/s41467-025-56885-9 39955281 10.1038/s41467-025-56885-9 PMC11829982

[54] Wente W, Efanov AM, Brenner M et al (2006) Fibroblast growth factor-21 improves pancreatic β-cell function and survival by activation of extracellular signal-regulated kinase 1/2 and Akt signaling pathways. Diabetes 55(9):2470–2478. 10.2337/db05-1435 16936195 10.2337/db05-1435

[55] Planavila A, Redondo-Angulo I, Ribas F et al (2015) Fibroblast growth factor 21 protects the heart from oxidative stress. Cardiovasc Res 106(1):19–31. 10.1093/cvr/cvu263 25538153 10.1093/cvr/cvu263

[56] Gomez-Samano MA, Grajales-Gomez M, Zuarth-Vazquez JM et al (2017) Fibroblast growth factor 21 and its novel association with oxidative stress. Redox Biol 11:335–341. 10.1016/j.redox.2016.12.024 28039838 10.1016/j.redox.2016.12.024 PMC5200873

[57] Lehtonen JM, Forsstrom S, Bottani E et al (2016) FGF21 is a biomarker for mitochondrial translation and mtDNA maintenance disorders. Neurology 87(22):2290–2299. 10.1212/WNL.0000000000003374 27794108 10.1212/WNL.0000000000003374 PMC5270510

[58] Sturm G, Karan KR, Monzel AS et al (2023) OxPhos defects cause hypermetabolism and reduce lifespan in cells and in patients with mitochondrial diseases. Commun Biol 6(1):22. 10.1038/s42003-022-04303-x 36635485 10.1038/s42003-022-04303-x PMC9837150

[59] Li B, Liu L (2022) Fibroblast growth factor 21, a stress regulator, inhibits Drp1 activation to alleviate skeletal muscle ischemia/reperfusion injury. Lab Invest 102(9):979–988. 10.1038/s41374-022-00787-7 35488034 10.1038/s41374-022-00787-7

[60] Knupp J, Arvan P, Chang A (2019) Increased mitochondrial respiration promotes survival from endoplasmic reticulum stress. Cell Death Differ 26(3):487–501. 10.1038/s41418-018-0133-4 29795335 10.1038/s41418-018-0133-4 PMC6370866

[61] Yao CH, Wang R, Wang Y, Kung CP, Weber JD, Patti GJ (2019) Mitochondrial fusion supports increased oxidative phosphorylation during cell proliferation. Elife 8:e41351. 10.7554/eLife.41351 30694178 10.7554/eLife.41351 PMC6351101

[62] Yu X, Arden C, Berlinguer-Palmini R et al (2022) Mitochondrial complex I subunit deficiency promotes pancreatic α-cell proliferation. Mol Metab 60:101489. 10.1016/j.molmet.2022.101489 35390502 10.1016/j.molmet.2022.101489 PMC9046450

[63] Tanday N, Flatt PR, Irwin N, Moffett RC (2020) Liraglutide and sitagliptin counter beta- to alpha-cell transdifferentiation in diabetes. J Endocrinol 245(1):53–64. 10.1530/JOE-19-0451 31977315 10.1530/JOE-19-0451

[64] Briant LJB, Dodd MS, Chibalina MV et al (2018) CPT1a-dependent long-chain fatty acid oxidation contributes to maintaining glucagon secretion from pancreatic islets. Cell Rep 23(11):3300–3311. 10.1016/j.celrep.2018.05.035 29898400 10.1016/j.celrep.2018.05.035 PMC6581793

[65] Richter MM, Kemp IM, Heeboll S et al (2024) Glucagon augments the secretion of FGF21 and GDF15 in MASLD by indirect mechanisms. Metabolism 156:155915. 10.1016/j.metabol.2024.155915 38631460 10.1016/j.metabol.2024.155915

[66] Hernandez-Cascales J (2018) Does glucagon have a positive inotropic effect in the human heart? Cardiovasc Diabetol 17(1):148. 10.1186/s12933-018-0791-z 30482191 10.1186/s12933-018-0791-z PMC6258156

[67] Gao C, Xiong Z, Liu Y et al (2024) Glucagon receptor antagonist for heart failure with preserved ejection fraction. Circ Res 135(5):614–628. 10.1161/CIRCRESAHA.124.324706 39011638 10.1161/CIRCRESAHA.124.324706 PMC11325917

[68] Rodriguez-Diaz R, Molano RD, Weitz JR et al (2018) Paracrine interactions within the pancreatic islet determine the glycemic set point. Cell Metab 27(3):549-558 e544. 10.1016/j.cmet.2018.01.015 29514065 10.1016/j.cmet.2018.01.015 PMC5872154

[69] Wei T, Cui X, Jiang Y et al (2023) Glucagon acting at the GLP-1 receptor contributes to β-cell regeneration induced by glucagon receptor antagonism in diabetic mice. Diabetes 72(5):599–610. 10.2337/db22-0784 36826938 10.2337/db22-0784 PMC10130488

[70] Tsuboi T, da Silva Xavier G, Holz GG, Jouaville LS, Thomas AP, Rutter GA (2003) Glucagon-like peptide-1 mobilizes intracellular Ca2+ and stimulates mitochondrial ATP synthesis in pancreatic MIN6 beta-cells. Biochem J 369(Pt 2):287–299. 10.1042/BJ20021288 12410638 10.1042/BJ20021288 PMC1223096

[71] Rutter GA, Sidarala V, Kaufman BA, Soleimanpour SA (2023) Mitochondrial metabolism and dynamics in pancreatic beta cell glucose sensing. Biochem J 480(11):773–789. 10.1042/BCJ20230167 37284792 10.1042/BCJ20230167

[72] Georgiadou E, Muralidharan C, Martinez M et al (2022) Mitofusins Mfn1 and Mfn2 are required to preserve glucose- but not incretin-stimulated β-cell connectivity and insulin secretion. Diabetes 71(7):1472–1489. 10.2337/db21-0800 35472764 10.2337/db21-0800 PMC9233298

[73] Dudek J (2017) Role of cardiolipin in mitochondrial signaling pathways. Front Cell Dev Biol 5:90. 10.3389/fcell.2017.00090 29034233 10.3389/fcell.2017.00090 PMC5626828

[74] Wohlfarter Y, Hagenbuchner J, Horzum U et al (2024) Mitochondrial cardiolipin metabolism controlled by tafazzin enables ferroptosis. BioRxiv 2024.10.25.620299 (Preprint). 26 Oct 2024. Available from: 10.1101/2024.10.25.620299

[75] Janer A, Morris JL, Krols M et al (2024) ESYT1 tethers the ER to mitochondria and is required for mitochondrial lipid and calcium homeostasis. Life Sci Alliance 7(1):e202302335. 10.26508/lsa.202302335 37931956 10.26508/lsa.202302335 PMC10627786

[76] Akan I, Olivier-Van Stichelen S, Bond MR, Hanover JA (2018) Nutrient-driven O-GlcNAc in proteostasis and neurodegeneration. J Neurochem 144(1):7–34. 10.1111/jnc.14242 29049853 10.1111/jnc.14242 PMC5735008

[77] Lockridge A, Jo S, Gustafson E et al (2020) Islet O-GlcNAcylation is required for lipid potentiation of insulin secretion through SERCA2. Cell Rep 31(5):107609. 10.1016/j.celrep.2020.107609 32375037 10.1016/j.celrep.2020.107609 PMC8114475

[78] Marsh SA, Powell PC, Dell’italia LJ, Chatham JC (2013) Cardiac O-GlcNAcylation blunts autophagic signaling in the diabetic heart. Life Sci 92(11):648–656. 10.1016/j.lfs.2012.06.011 22728715 10.1016/j.lfs.2012.06.011 PMC3477499

[79] Tan EP, McGreal SR, Graw S et al (2017) Sustained O-GlcNAcylation reprograms mitochondrial function to regulate energy metabolism. J Biol Chem 292(36):14940–14962. 10.1074/jbc.M117.797944 28739801 10.1074/jbc.M117.797944 PMC5592672

[80] Durning SP, Flanagan-Steet H, Prasad N, Wells L (2016) O-Linked β-N-acetylglucosamine (O-GlcNAc) acts as a glucose sensor to epigenetically regulate the insulin gene in pancreatic beta cells. J Biol Chem 291(5):2107–2118. 10.1074/jbc.M115.693580 26598517 10.1074/jbc.M115.693580 PMC4732198

