# Supplementary material for: Adaptive mechanisms in pancreatic islets counteract mitochondrial dysfunction in Barth syndrome
Source: Diabetologia. 2025 Oct 24;69(1):173–97. doi: 10.1007/s00125-025-06575-4 (PMC12685984; doi:10.1007/s00125-025-06575-4)
Supplement: Supplementary file 1 — ESM (PDF 4617 KB) [file 125_2025_6575_MOESM1_ESM.pdf]

Supplementary methods information

All substances, if not indicated differently were purchased by Sigma/Merck, VWR, Thermo Fisher, Roth and Biomol. Filtersets and beamsplitter for inverted epifluorescence microscopy were manufactured by Zeiss.

Animal models and genotyping

The genotype of the animals was identified by polymerase chain reaction (PCR). Tissue samples were incubated in DNA extraction buffer at 65 °C for 15 min, then vortexed and incubated at 98 °C for 2 min. The extracted DNA was amplified using the according primers listed below. All mouse strains used in this article have the NNT protein and are on a C57BL/6N background.

PCR protocols were following:

ShTaz : 95 °C 5 min – (95 °C 30 sec – 60 °C 30 sec – 72 °C 1 min) x40 – 72 °C 10 min

Orp : 95 °C 3 min – (95 °C 30 sec – 64 °C 30 sec – 72 °C 1 min) x20 – (95 °C 30 sec – 54 °C 30 sec – 72 °C 1 min) x10 – 72 °C 5 min

Primers for genotyping :

Rosa JD 72 : 5' CCA TGG AAT TCG AAC GCT GAC GTC 3'

Rosa JD 73 : 5' TAT GGG CTA TGA ACT AAT GAC CC 3'

Rosa JD 74: 5' GAG ACT CTG GCT ACT CAT CC 3'

Rosa JD 75: 5' CCT TCA GCA AGA GCT GGG GAC 3'

MS 282: 5' AAA GTC GCT CTG AGT TGT TAT 3'

MS 284: 5' GGA GCG GGA GAA ATG GAT ATG 3'

MS 305: 5' GGG CTA TGA ACT AAT GAC CCC G 3'

E8 for Bl/6N: 5' TAT TGG CTA CAC AGA CCT TCC 3'

E8_rev for Bl/6N:5' TGA CGT GAC TCA TTG TAC CA 3'

E6-12 for Bl/6J: 5' GTA GGG CCA ACT GTT TCT GC 3'

E6-12_rev for Bl/6J: 5' TCC CCT CCC TTC CAT TTA GT 3'

Pancreatic islet isolation and culture

Isolation of mouse pancreatic islets was performed as previously described [25]. Briefly, mice were anaesthetized with isoflurane and sacrificed via cervical dislocation. Afterwards, the body was opened ventrally, and the ampulla connecting the pancreas and small intestine was clamped. Next, the pancreas was perfused via the pancreatic duct using an ice-cold collagenase solution (Collagenase P, 11213865001, Merck/Sigma Aldrich) with a concentration of 0.63 mg/ml. The perfused pancreas was digested for 20 min in a water bath at 37 °C and washed 3x in Krebs-Henseleit-buffer (KHB), containing 24 mM $NaHCO_3$, 120 mM NaCl, 4.8 mM KCl, 1.2 mM $MgCl_2$, 2.5 mM $CaCl_2$, 5 mM HEPES, 0.2% Bovine serum albumin (BSA), 1% Penicillin/Streptomycin (P/S) and 10 mM glucose. Afterwards, pancreatic islets were hand-picked with a 10 µl pipet under a stereo microscope and separated from the exocrine tissue. The collected pancreatic islets were cultured in RPMI 1640 (ref: 11875093, Gibco^{TM}) supplemented with 10% (v/v) Fetal Bovine Serum (FBS) and 1% (v/v) P/S under 5% CO_2 and 37 °C, until further use. The pancreatic islets were cultured for 1 - 3 days and groups of similar size WT and *Taz*-KD islets were formed before each experiment.

Sample preparation for lipidomics and enzymatic assays

Homogenization of pancreatic islets was necessary for enzymatic assays and lipidomics sample preparation. It was achieved by initial dispersion of isolated pancreatic islets with trypsin and subsequent sonication (sonic dismembrator model 705, ref: 86853K-09-15, Thermo Fisher). After the final centrifugation step of the pancreatic islet dispersion, the pellet was reconstituted in Dulbecco's phosphate-buffered saline (DPBS, ref: D8662, Merck/Sigma Aldrich) and the solution was sonicated at 50% for 2 min (20 s pulses and 20 s rest time) on ice.

Lipidomics

Lipid extraction for mass spectrometry lipidomics

Mass spectrometry (MS)-based lipid analysis was performed by Lipotype Lipidomics GmbH (Dresden, Germany) as described [26]. Lipids were extracted using a chloroform/methanol procedure [27]. Samples were spiked with internal lipid standard mixture containing: cardiolipin 14:0/14:0/14:0/14:0 (CL), ceramide 18:1;2/17:0 (Cer), diacylglycerol 17:0/17:0 (DAG), hexosylceramide 18:1;2/12:0 (HexCer), lyso-phosphatidate 17:0 (LPA), lyso-phosphatidylcholine 12:0 (LPC), lyso-phosphatidylethanolamine 17:1 (LPE), lyso-phosphatidylglycerol 17:1 (LPG), lyso-phosphatidylinositol 17:1 (LPI), lyso-phosphatidylserine 17:1 (LPS), phosphatidate 17:0/17:0 (PA), phosphatidylcholine 17:0/17:0 (PC), phosphatidylethanolamine 17:0/17:0 (PE), phosphatidylglycerol 17:0/17:0 (PG), phosphatidylinositol 16:0/16:0 (PI), phosphatidylserine 17:0/17:0 (PS), cholesterol ester 16:0 D7 (CE), sphingomyelin 18:1;2/12:0;0 (SM), triacylglycerol 17:0/17:0/17:0 (TAG). After extraction, the organic phase was transferred to an infusion plate and dried in a speed vacuum concentrator. The dry extract was re-suspended in 7.5 mM ammonium formate in chloroform/methanol/propanol (1:2:4; V:V:V). All liquid handling steps were performed using Hamilton Robotics STARlet robotic platform with the Anti Droplet Control feature for organic solvents pipetting.

MS data acquisition

Samples were analyzed by direct infusion on a QExactive mass spectrometer (Thermo Scientific) equipped with a TriVersa NanoMate ion source (Advion Biosciences). Samples were analyzed in both positive and negative ion modes with a resolution of $R_{m/z=200}$=280000 for MS and $R_{m/z=200}$=17500 for Tandem mass spectrometry (MSMS) experiments, in a single acquisition. MSMS was triggered by an inclusion list encompassing corresponding MS mass ranges scanned in 1 Da increments [28]. Both MS and MSMS data were combined to monitor CE, DAG and TAG ions as ammonium adducts; LPC, LPC O-, PC and PC O- as formiate adducts; and CL, LPS, PA, PE, PE O-, PG, PI and PS as deprotonated anions. MS only was used to monitor LPA, LPE, LPE O-, LPG and LPI as deprotonated anions, and Cer, HexCer and SM as formiate adducts.

Lipidomics data analysis and post-processing

Data were analyzed with a lipid identification software based on LipidXplorer [26, 29]. Data post-processing and normalization were performed using an in-house developed data management system. Only lipid identifications with a signal-to-noise ratio >5, and a signal intensity 5-fold higher than in corresponding blank samples were considered for further data analysis.

Immunohistochemistry

Cryoslices

The number of alpha-, beta-, or delta cells of the *in vivo Taz*-KD model were quantified by immunohistochemistry (IHC). After isolation of the whole pancreas, the tissue was washed in PBS and fixed in 4% Perfluoroalkoxy alkanes (PFA) overnight at RT. On the next day, the tissue was washed for 4 h in PBS, before it was transferred to a 30% sucrose solution and kept for 3 h. Subsequently, the whole pancreas was rapidly frozen in Tissue-Tek® O.C.T.™ (SA62550-01, Science services) using liquid nitrogen-cooled isopentane. Cutting was performed at a Leica cryostat setting a thickness of 5 µm and a temperature of -15 °C. Pancreas slices were dried for 30 min, before being washed with PBS and treated with 3% goat serum for 1 h in a wet chamber. Subsequently, 50 µl of primary anti-insulin together with anti-glucagon or anti-somatostatin antibodies (Table 1, 1:200 diluted) were added and incubated overnight in wet chamber at 4 °C. On the following day, slides were again washed with PBS and 50 µl of Alexa 594 and Alexa 488 secondary antibodies (Table 1, 1:400 diluted) were added for 1 h 15 min at RT. After a final washing step slides were dried and mounted with Dako mounting media. Imaging was performed with the Axio Observer 7 microscope (Zeiss, Germany) using a 20x air objective.

Paraffin slices

Paraffin embedding was either performed on the whole pancreas of shTaz animals of the *in vivo* model or on isolated pancreatic islets of the *in vitro* shTaz model. Freshly isolated pancreatic islets were clotted before PFA fixation. First, the pancreatic islets were incubated in 3 ml RPMI 1640 medium on top of 1 ml agarose overnight at 37 °C and 5% CO_2. Subsequently, the pancreatic islets were clotted in a mixture of human platelet-poor plasma, Hepatoquick and 10% $CaCl_2$. The clotted pancreatic islets and the whole pancreas were placed for 24 h in PFA and then embedded with an ethanol, xylol and paraffin protocol in a tissue processor (SLEE medical GmbH,

Germany). Paraffin blocks were cut with a microtome and stained against different cell types (namely α-, β-, or delta cells), Ki67 and cleaved caspase-3 (Table 1). After antibody staining, the slides were treated with DAPI (ref: 10116287, Thermo Fisher) to visualize the nuclei. Imaging was performed with the Axio Observer 7 system (Zeiss, Germany) using a 20x air objective. The analysis was performed in ImageJ and positive stained cells were counted using the cell counter plugin in ImageJ.

Glucose uptake

Using the Glucose Uptake-Glo™ assay kit, glucose uptake into the pancreatic islets was assessed. Groups of 5, 10 and 20 islets were washed in glucose-free SILAC Flex-medium and imaged using a stereo microscope with an Axiocam 105 color (for normalisation). Afterwards, they were incubated for 1 h (37 °C and 5% CO2) in glucose-free SILAC RPMI Flex medium supplemented with 20 mM of 2-deoxyglucose (2DG) to mimic glucose uptake. Once inside the cells, 2DG is by hexokinases (in beta cells mostly glucokinase) phosphorylated to 2-deoxy-D-glucose-6-phosphate (2DG6P), which cannot be further metabolised. Therefore, the 2DG6P concentration is proportional to the level of glucose uptake into the cell. Pancreatic islets samples without 2DG loading were used as a negative control. After the incubation period, the standard protocol provided by the company was followed and subsequently, luminescence was recorded using a Clariostar plate reader (BMG).

Western Blot

Groups of 300 islets were collected in ice-cold PBS and centrifuged at 2000 rcf for 5 min (cooled down to 4 °C). The resulting pellet was snap-frozen in liquid nitrogen and stored at -20 °C until further use. Cell lysis was performed for 30 min on ice using a cell lysis buffer (10 mM Tris-HCl, 10 mM NaCl, 0.1 mM EDTA, 0.5% Triton-X-100, 0.2% NaN_3, 200 µM PMSF, 1:100 protease/phosphatase inhibitor cocktail). Subsequently, the samples were centrifuged for 30 min at 14,000 rcf and 4 °C. The supernatant was transferred into a new reaction tube and protein concentration was assessed. The proteins were separated according to their molecular mass using sodium dodecyl sulfate-polyacrylamide gel electrophoresis (SDS-PAGE). Therefore, the samples were mixed with 2x Laemmli buffer (ref: S3401, Merck) in a 1:1 ratio and denatured for 5 min at 95 °C. Then, the denatured protein samples were added to a 10 or 12.5% SDS-

polyacrylamide gel and separated at 100 V and 30 - 40 mA. The protein marker peqGOLD IV (ref: 27-2110, VWR) was used to quantify protein size. Before the separated proteins could be visualized with antibodies, they first had to be transferred to a methanol-activated polyvinylidene difluoride (PVDF) membrane using the semi-dry approach. Here, the SDS gel and the PVDF membrane were packed between Whatman papers in a semi-dry chamber. After applying 25 V and 1.3 A for 7 min, the membrane was taken out of the chamber and unspecific binding sites were blocked using Tris-buffered saline with 0.1% Tween® 20 detergent (TBST) and 5% BSA for 1 h at RT. Primary antibodies were diluted 1:500 in TBST with 1% BSA and incubated with the membrane overnight at 4 °C. On the next day, membranes were washed 3 times for 10 min with TBST before incubation with HRP-conjugated secondary antibody for 1 h at RT. After another washing step with TBST (3 times for 10 min), the enhanced chemiluminescence (ECL) solution (ref: 1705060, Bio-Rad was added to visualise the proteins on the membrane. Western blot images were acquired with a gel documentation system (Gel Doc XR+, Bio-Rad, Germany), analyzed using the Image Lab software and normalized to the housekeeping protein (β-actin, Table 1).

Presentation and statistical analysis of WB data were performed in a paired manner. One *Taz*-KD sample was always compared to another identically prepared WT sample. Therefore, the WT samples are all displayed at 100% and do not show an error among them. However, the variation among the samples is presented in separated figures in the supplements. The reason being that depending on the functional experiments some samples were frozen after 1 day and others after 2 or 3 days in culture.

Hexokinase I-IV (Glucokinase assay)

The fluorometric glucokinase activity assay kit (ab273303) was used to study glucokinase (GCK) activity. In this assay, GCK from homogenized pancreatic islet samples converts glucose into glucose-6-phosphate, which is then further metabolized, thereby reducing a probe and producing a fluorescent product. Briefly, groups of 150 freshly isolated pancreatic islets were hand-picked, size-matched and washed in PBS to avoid non-islet cell contamination. Afterwards, the islets were dispersed and homogenised. GCK assay buffer was supplemented with 2.5 mM DTT immediately before use. Then, if not stated otherwise, the protocol provided by the manufacturer was followed. Fluorescent intensity (λ_{ex} = 540/20 nm, BS = 560 nm, λ_{em} = 590/20 nm) was measured using a Clariostar plate reader (BMG). For each sample, a corresponding sample well containing all

components but not the GCK sample substrate was measured (background control). The obtained fluorescence values were background corrected and compared to a NADPH standard curve. Finally, the results were normalized using a BCA protein assay.

Measurement of the mitochondrial oxygen consumption rate and extracellular acidification rate

Oxygen consumption (OCR) and extracellular acidification rate (ECAR) of whole pancreatic islets were assessed using a Seahorse XFe96 Analyzer (Agilent). One day before the experiments, sensor cartridges were prepared by adding calibrant XF (100840-000, Agilent) and incubating overnight at 37 °C (no additional CO_2). On the day of the experiment, a spheroid XFe96 microplate (102978-100, Agilent) was coated with poly-L-lysine, and each well was filled with 175 µl of pre-warmed Seahorse XF RPMI medium (103576-100, Agilent) supplemented with 0.1% FBS, 2.8 mM glucose (103577-100, Agilent), and 2 mM glutamine (10359-100, Agilent). Groups of 15 islets were seeded with 5 µl volume into the corresponding detent at the bottom of the wells [30] and the plate was equilibrated for 1 h in a non-CO_2 incubator. Afterwards, the protocol of the Seahorse XF Cell Mito Stress Test Kit (103015-100, Agilent) combined with an initial glucose stimulation (2.8, 10, or 20 mM) was conducted. The concentrations for the inhibitors were optimised in initial islet experiments and set to 4.5 µM of oligomycin, 1 µM of FCCP, 5 µM of antimycin A and rotenone. The assay was performed with at least six replicates per condition. Samples that did not match the following criteria were excluded: Absolute oxygen concentration between 130 - 160 mmHg and stable throughout the assay. The spheroid plate was inspected after the measurement to see if the islets were still inside the small detent of each well. Analysis was done with Wave software (version 2.6.3) and Prism (version 9.4).

Nutrient dependencies and capacities were measured using the Seahorse XF Mito Fuel Flex Test Kit (103260-100, Agilent). In detail, three inhibitors (4 µM of etomoxir, 2 µM of UK5099 and 3 µM of BPTES) were used to test each genotype's nutrient dependencies and capacities. First, only one pathway was blocked by the injection of one inhibitor and subsequently, the other two pathways were blocked together with the next injection. Different combinations of inhibitors could determine the nutrient dependencies and capacities. The Seahorse assay medium for this nutrient dependency test was based on the Seahorse XF RPMI medium supplemented with 10 mM glucose, 2 mM glutamine, 1 mM pyruvate (103578-100, Agilent), and 0.1% FBS.

Calcium measurements

Cytosolic calcium measurements

Intracellular cytosolic calcium levels of dispersed and whole pancreatic islets were measured using the ratiometric fluorescent dye Fura-2 AM (λ_{ex1} = 340/30 nm, λ_{ex2} = 387/15 nm, BS = 409 nm λ_{em} = 510/90 nm). Groups of 10 similar size WT and *Taz*-KD islets were loaded for 2 h with 5 µM Fura-2 AM under standard culture conditions. After washing and starvation with 2 mM glucose in KHB for 15 min, WT and *Taz*-KD islets were placed in the measurement chamber of the Axio Observer 7. Baseline was recorded for 10 min and then, glucose concentration was raised to 20 mM glucose. KCl (30 mM) was used as a positive control at the end of the experiment. In another set of experiments, tolbutamide was used in titration protocol (1 – 100 µM) in the presence of 2 mM glucose.

Mitochondrial calcium measurements

The Mito-Pericam calcium sensor was used to test mitochondrial calcium concentration in dispersed pancreatic islets [31, 32]. Adenoviral transduction was performed by adding 0.5 µl of Mito-Pericam adenovirus on top of a coverslip with 25 dispersed pancreatic islets in 2 ml RPMI medium (10% FBS, 1% P/S). Two to three days after transduction, cells were measured (λ_{ex} = 405/20 nm, BS = 505 nm, λ_{em} = 550/100 nm) using an inverted epifluorescence microscope Axio Observer 7 (Zeiss) and a 63x oil objective (Zeiss). The baseline was recorded for 5 min in KHB with 2 mM glucose, before a 20 mM glucose solution (final concentration) was applied. After 10 - 20 min measurement in high glucose conditions, 30 mM of KCl was applied to have a depolarizing control.

Calcium measurement in ER-lumen

The ER-targeted biosensor D4ER was used to measure calcium levels in the ER lumen. A rat insulin promotor linked to the D4ER gene allowed specific transfection only in pancreatic beta cells [33]. Groups of 25 dispersed islets were seeded on to coverslips in 2 ml RPMI medium (10% FBS, 1% P/S) and transduced by 0.5 µl of adenovirus encoding D4ER. 2 - 3 days after transduction, cells were measured (λ_{ex}= 445/25 nm, BS = 505 nm, λ_{em1} = 475 nm, λ_{em2} = 540/20 nm) using an inverted epifluorescence microscope Axio Observer 7 (Zeiss) and a 63x oil objective

(Zeiss). The baseline was recorded for 5 min in KHB with 2 mM glucose, before a 20 mM glucose solution (final concentration) was applied. At the end of the experiment, 3 µM thapsigargin (final concentration) was added to empty the ER calcium storage.

Redox histology

Mito-roGFP2-Orp1 crossbreed with shTaz mice (MiOxTaz mice) were sacrificed via 2:1 Ketamin/Rompun (1 g/kg Ketavet®/ 100 mg/kg Rompun®) injection. Redox histology was performed as previously described with some modifications [24, 34]. First, cardiac perfusion with 25 ml of 50 mM N-Ethylmaleimide (NEM) diluted in PBS was used to block free thiols and prevent artificial sensor oxidation. Afterwards the pancreas was inflated with 5 ml of the same solution. The whole pancreas was removed and fixation was performed overnight at RT with 4% PFA. To improve cryoslice quality, pancreas samples were additionally treated with 30% sucrose diluted in PBS for 3 h before embedding in Tissue Tek. All samples were cut at 5 µm using a cryostat at -15 °C. Pancreas slides were dried for 30 min before being washed with PBS and treated with 3% goat serum for 1 h in a wet chamber. Subsequently, 50 µl of primary anti-insulin antibody (Table 1, 1:200 diluted) was added and incubated overnight in a wet chamber at 4 °C. On the following day, slides were again washed with PBS and 50 µl of Alexa 594 secondary antibody (Table 1, 1:400 diluted) was added for 1 h 15 min at RT. After a final washing step, slides were dried and mounted with Dako mounting media. Imaging was performed with the previously described Axio Observer 7 system using a 20x air objective. The roGFP2-Orp1 fluorescence was measured at 500 to 550 nm with either 405/20 nm or 470/40 nm excitation. Pancreatic islets inside the pancreas tissue were localised using a mCherry filter set. Analysis was performed on ImageJ using a self-written code to automate the procedure and minimize risk of bias in setting thresholds.

After calculating the ratio for each image, both genotypes (WT and *Taz*-KD) were compared by calculating the percentage change:

$$change\ in\ \frac{405}{488}\ nm\ ratio = \frac{(ratio\ value - WT_{mean})}{WT_{mean}}\ x\ 100$$

ratio value: either WT or *Taz*-KD ratio value; WT_{mean}: mean value for all the WT ratio values.

Confocal and STED microscopy

Directly after islet isolation, groups of 50 pancreatic islets were dispersed and seeded on a coverslip (170 µm thickness, # 1.5, Marienfeld, Gemany). On the next day, the dispersed islet cells were washed with 10 mM glucose KHB and stained with 30 nM MitoTracker™ Deep Red in RPMI 1640 (only 0.1% FCS) for 10 min at RT. After three washing steps, the imaging was carried out in 10 mM glucose KHB at RT. The samples were imaged on an inverted STED microscope (Expert Line, Abberior Instruments, Göttingen, Germany) using the according control software Imspector V16.3. A 100x silicon immersion objective with a numerical aperture of 1.4 (UPLSAPO100XS, Olympus, Hamburg, Germany) and a pinhole size of 90.0 µm (1.08 airy units) was used. In order to have an overview of the pancreatic islet cells with the labeled mitochondria, single confocal 80 x 80 µm scanning with 200 nm (in XY axis) was performed. at excitation of 640 nm. This was followed by another confocal scanning of a randomly selected cell, this time with finer XY pixel size (40 nm) and a Z-stack to generate a 3D image (voxel size: 40 x 40 x 300 nm^3). The detection filter and total pixel dwell time were set to 650 – 720 nm and 5 µs, respectively. Finally, parts of the cellular volume were recorded in STED mode. The STED experiments were performed using "rescue settings" which reduce the photobleaching of the Mito tracker. The 775 nm depletion laser pulsed at 40% of the maximal power of 1250 mW (corresponding to 75 – 85 mW in the focus, repetition rate of 40 MHz) with no gating and total dwell time of 17.5 µs. The STED images were recorded with a voxel size of 20 x 20 x 300 nm^3. In addition to confocal and STED image stacks, the residual excitation by the STED beam (so called "re-excitation") was recorded and later subtracted from the STED recordings after deconvolution.

Data were plane-wise linearly deconvoluted using a Wiener filter with theoretical point spread functions and manually adjusted regularisation parameter. For deconvolution and subtraction of the re-excitation custom-written routines in MATLAB were used. Next, all images were preprocessed in ImageJ for feature extraction using smoothing and shot-noise reduction (bilateral filter), background subtraction (rolling-ball algorithm) and sharpening (unsharp mask). Subsequently, threshold-based image segmentation and 3D-rendering was performed in Imaris (version 9.6). The resulting disconnected surfaces were grouped based on their surface area into three classes: Class A = 0.3 - 3 μm^2, Class B = 3 - 10 μm^2, and Class C > 10 μm^2. The morphological parameters, including surface area, volume, number of single mitochondria, sphericity, and bounding boxes were automatically calculated by Imaris software based on each disconnected object. The sphericity describes how spherical an object is and can be calculated via $\frac{\pi^{\frac{1}{3}} \times (6 \times volume)^{\frac{2}{3}}}{surface\ area}$. The bounding box of a single mitochondrion describes the minimal rectangular box which fully encloses the object. Furthermore, a nearest neighbor analysis was

performed with a custom-written MATLAB protocol. The program gives the number of neighboring objects for each object in a radius of 2.5 µm.

RNA Sequencing

RNA samples were diluted in RNase-free water and 5 µl of RNA sample were sent for sequencing, which was performed by Novogene (Novogene GmbH, Cambridge, UK). The quantity and quality of the RNA samples were assessed using the following methods. Preliminary quality control was performed on 1% agarose gel electrophoresis to test RNA degradation and potential contamination. Sample purity and preliminary quantitation were measured using Bioanalyzer 2100 (Agilent Technologies, USA) and it was also used to check the RNA integrity and final quantitation.

For library preparation, we used the Novogene NGS RNA Library Prep Set (PT042). The mRNA present in the total RNA sample was isolated with magnetic beads of oligos d(T)25. This method is known as polyA-tailed mRNA enrichment. Subsequently, mRNA was randomly fragmented and cDNA synthesis proceeded using random hexamers and the reverse transcriptase enzyme. Once the synthesis of the first chain is finished, the second chain is synthesized with the addition of an Illumina buffer (non-directional library preparation). With this and together with the presence of dNTPs, RNase H and polymerase I from *E. Coli*, the second chain will be obtained by Nick translation. The resulting products go through purification, end-repair, A-tailing and adapter ligation. Fragments of the appropriate size are enriched by PCR, where indexed P5 and P7 primers are introduced, and final products are purified.

The library was checked with Qubit 2.0 and real-time PCR for quantification and bioanalyzer Agilent 2100 for size distribution detection. Quantified libraries were pooled and sequenced on the Illumina Novaseq X platform, according to effective library concentration and data amount using the paired-end 150 strategy (PE150).

Gene ontology (GO) enrichment analysis was performed to identify molecular processes associated to gene alterations. Standard Z-Scores from Fragments Per Kilobase Million (FPKM) data were used to compare gene expression levels inside different sample groups (*in vivo* or *in vitro*). Furthermore, a delta Z-Score (ΔZ) was calculated to compare the difference over several different sample groups. For this, Z-scores from the mean FPKM values of WT *in vivo*, WT *in*

vitro, *Taz*-KD *in vivo* and *Taz*-KD *in vitro* were calculated. Then, the absolute value of ΔZ was calculated using the following equation:

$$\Delta Z = |(Z_{Taz-KD\ in\ vitro} - Z_{Taz-KD\ in\ vivo}) - (Z_{WT\ in\ vitro} - Z_{WT\ in\ vivo})|$$

ESM Table 1: QPCR TaqMan primers.

Primer	company	Assay ID
Tafazzin	Thermo Fisher	Mm00504978_m1
GAPDH	Thermo Fisher	Mm99999915_g1

Antibody list

ESM Table 2: Antibodies used for WB or IHC.

Antibody	Company	Reference	Experiment
anti-Atg7	Cell Signaling	2631T	WB
anti-β-actin	proteintech	HRP-66009	WB
anti-cleaved Caspase-3	Cell signaling	9664	IHC
anti-Catalase	Cell Signaling	14097	WB
anti-Glucagon	abcam	ab10988	IHC
anti-GLUT2	Merck/Sigma Aldrich	07-1402-I	WB
anti-GPX4	abcam	ab125066	WB
anti-Insulin	abcam	ab181547	IHC
anti-Ki67	Cell signaling	12202	IHC

anti-LAMP1	DSHB	1D4B	WB
anti-LAMP2	DSHB	ABL-93	WB
anti-LC3B	Cell Signaling	2775	WB
anti-Mouse IgG HRP	Agilent Technologies	P044701-2	WB
anti-NOX4	biotechne	NB110-58849	WB
anti-Nrf2	proteintech	16396	WB
anti-O-GlcNAc (RL2)	Thermo Fisher	11518842	WB, IHC
anti-PDX1	abcam	47308	IHC
anti-PINK1	Thermo Fisher	11575333	WB
anti-Prk8	Cell Signaling	4211S	WB
anti-Prx3	abcam	ab73349	WB
anti-Mouse IgG Alexa Fluor™ 488	Thermo Fisher	A-21141	IHC
anti-Rabbit IgG Alexa Fluor™ 555	Thermo Fisher	A-21429	IHC
anti-Rabbit IgG Alexa Fluor™ 594	Thermo Fisher	A-11012	IHC
anti-Rabbit IgG HRP	R&D Systems	HAF008	WB
anti-Somatostatin	abcam	ab30788	IHC
Anti-Glut1	proteintech	21829-1-AP	WB
Anti-ATF4	proteintech	10835-1-AP	WB
Anti- eIF2-alpha total	Cell signaling	5324	WB

Anti-eIF2-alpha phospho	Cell signaling	9721	WB
Anti-GDF-15	proteintech	27455-1-AP	WB

ESM Figure 01

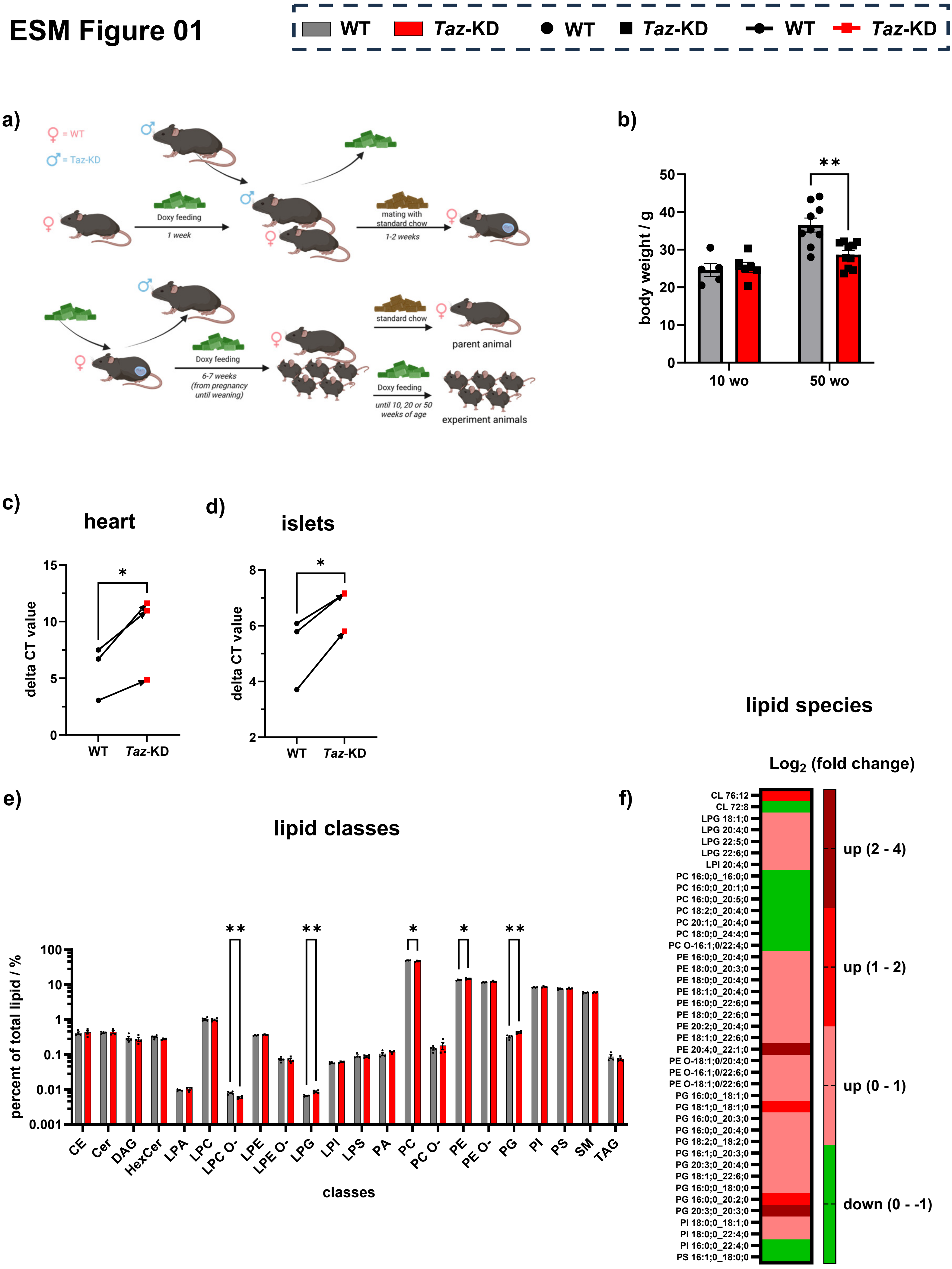

ESM Figure 1:

(**A**) ShTaz doxycycline breeding scheme. (**B**) Body weight of *Taz*-KD and WT at 10 and 50 wo, N (10 wo, WT) = 5, N (10 wo, *Taz*-KD) = 6, N (50 wo, WT) = 9, N (50 wo, *Taz*-KD) = 10. Paired analysis of *Taz* gene expression in heart (**C**) and pancreatic islet (**D**) tissue, N = 3. (**E**) Complete lipid class profile (logarithmic scaling) of pancreatic islets from 20 wo WT and *Taz*-KD, N = 4. (**F**) Significantly ($p < 0.05$) altered lipid species in *Taz*-KD pancreatic islets. Data represent mean ± SEM (indicated by error bars); N numbers indicate number of animals; statistical significance was determined by unpaired Student *t* test: *$p < 0.05$, **$p < 0.01$. Abbreviations: weeks of age (wo), *Tafazzin*-Knockdown (*Taz*-KD), Wildtype (WT), glucose tolerance test (GTT).

ESM Figure 02

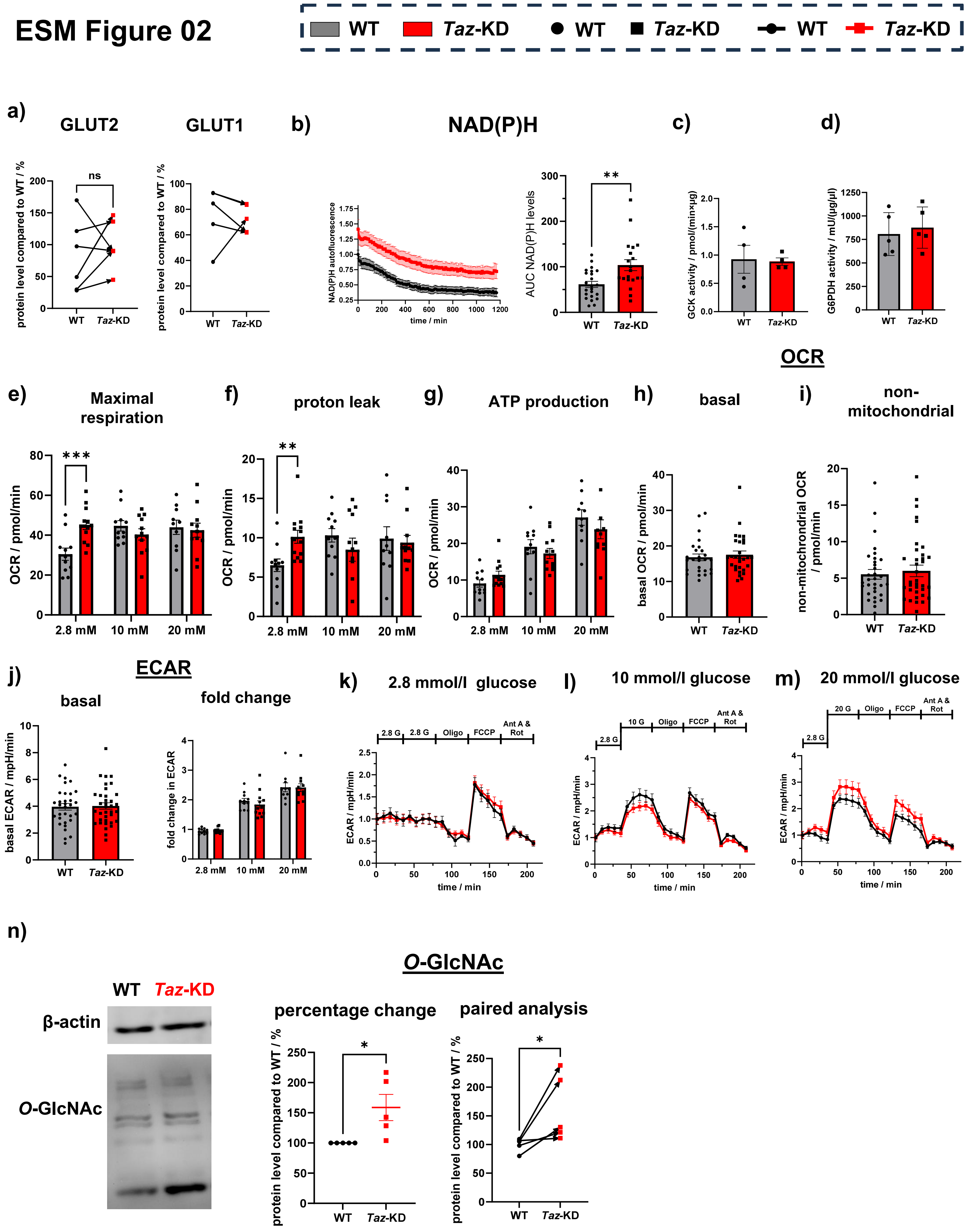

ESM Figure 02

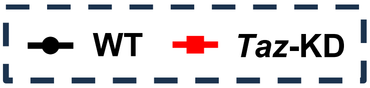

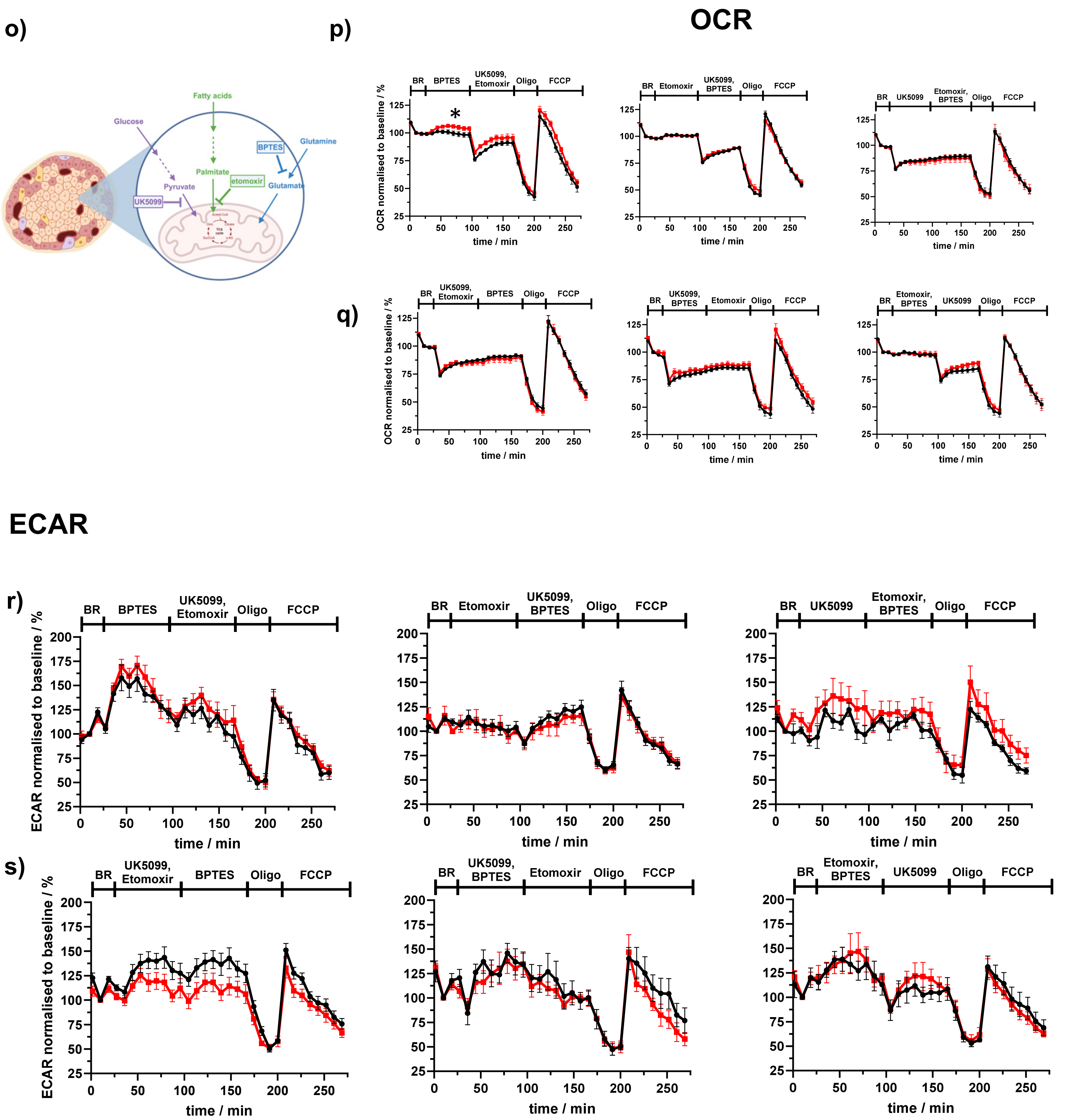

ESM Figure 02

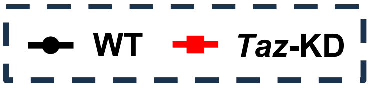

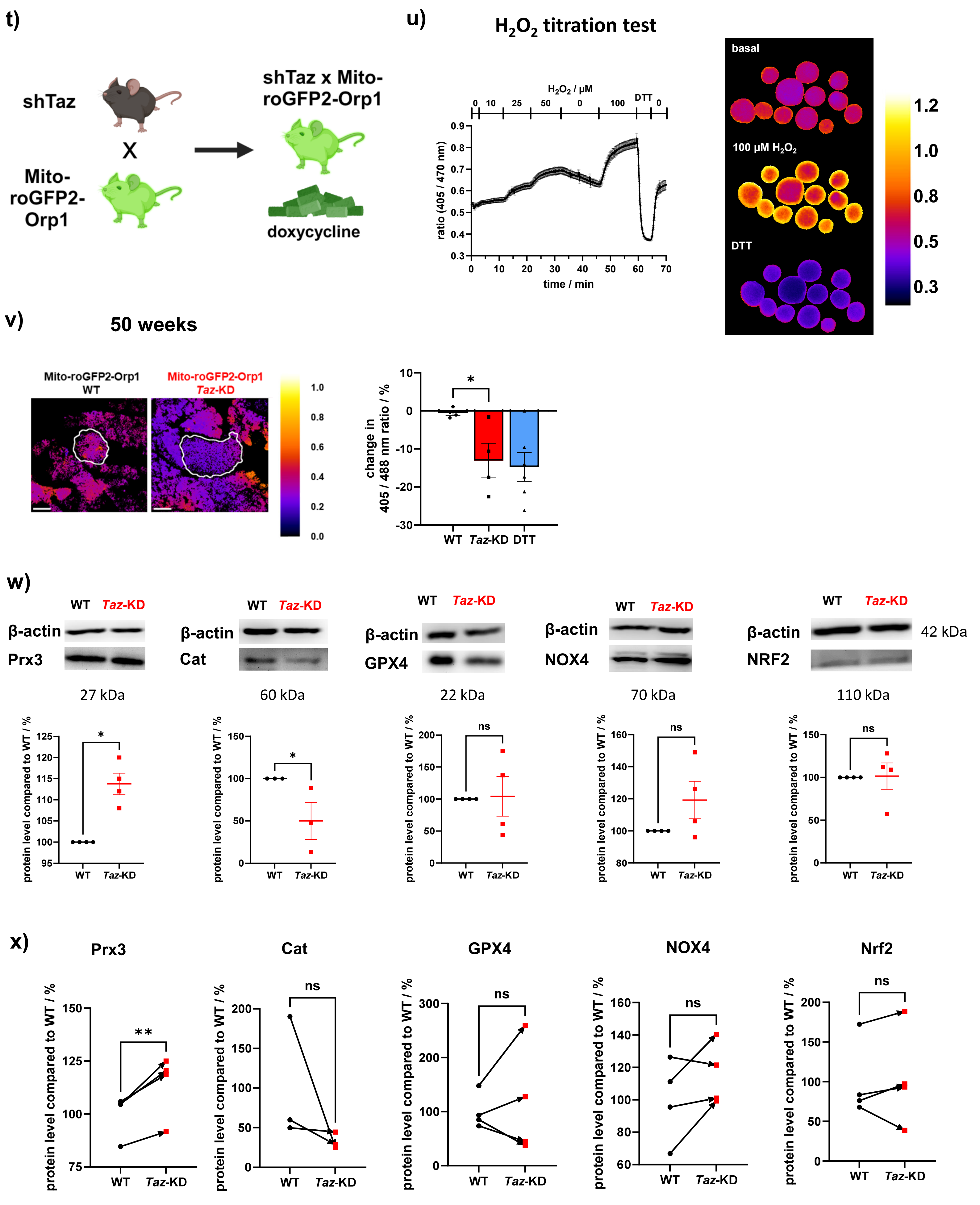

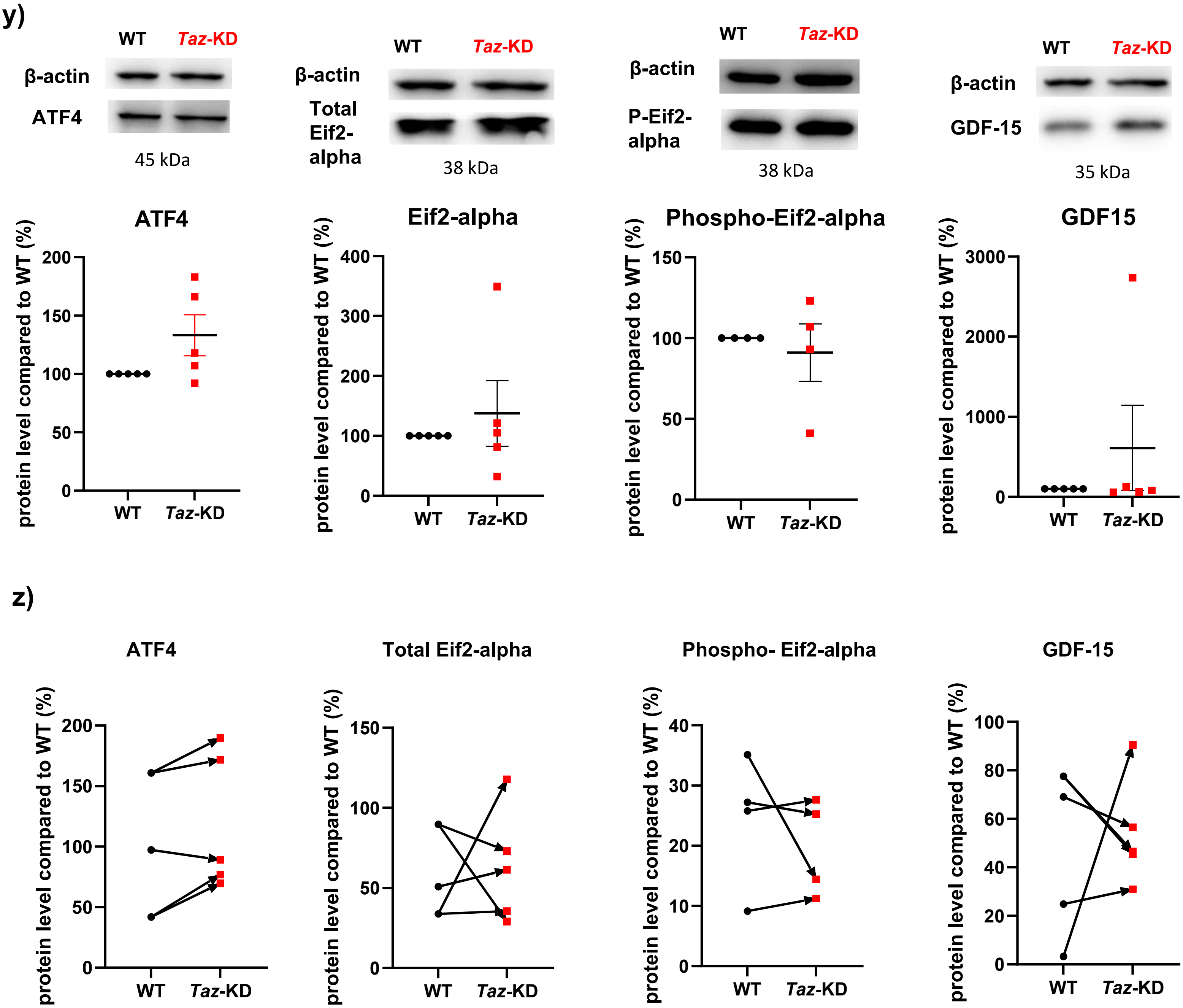
y)
WT
Taz-KD
β-actin
ATF4
45 kDa
β-actin
Total Eif2-alpha
38 kDa
β-actin
P-Eif2-alpha
38 kDa
β-actin
GDF-15
35 kDa
ATF4
Eif2-alpha
Phospho-Eif2-alpha
GDF15
protein level compared to WT (%)
z)
ATF4
Total Eif2-alpha
Phospho- Eif2-alpha
GDF-15
protein level compared to WT (%)

ESM Figure 2:

(**A**) Paired analysis of GLUT2 and GLUT1 protein levels in 20 wo WT and *Taz*-KD pancreatic islets, N =4-6. (**B**) NAD(P)H autofluorescence measurement (left) and AUC analysis (right) of 20 wo mito-roGFP2-Orp1/WT and mito-roGFP2-Orp1/*Taz*-KD pancreatic islets in parallel to H_2O_2 recordings, n (WT) = 23, n (*Taz*-KD) = 20, from 8 animals. Quantification of GCK (**C**) and G6PDH (**D**) enzyme activity. N (GCK) = 4, N (G6PDH) = 5. (**E**) Calculated maximal respiration, (**F**) proton leak and (**G**) ATP production from OCR of WT and *Taz*-KD 20 wo pancreatic islets. n (WT) = 11, n (*Taz*-KD) = 13, n number of experiments include N (WT) = 5 and N (*Taz*-KD) = 4. (**H**) Quantification of basal and (**I**) non-mitochondrial OCR levels of WT and *Taz*-KD 20 wo pancreatic islets. n (WT) = 32, n (*Taz*-KD) = 36 (**J**) Quantification of basal ECAR (left), and ECAR fold change (right) in response to glucose (2.8, 10, and 20 mmol/l) of WT and *Taz*-KD 20 wo pancreatic islets. n (basal ECAR, WT) = 32, n (basal ECAR, *Taz*-KD) = 36, n (ECAR fold change, WT) = 11, n (ECAR fold change, *Taz*-KD) = 12. ECAR kinetic curves of 20 wo WT and *Taz*-KD pancreatic islets in response to 2.8 mmol/l (**K**), 10 mmol/l (**L**) and 20 mmol/l (**M**) glucose stimulation followed by the addition of inhibitors of the respiratory chain complexes (Oligo, Ant A and Rot) and uncoupler (FCCP). n (WT) = 11, n (*Taz*-KD) = 13. (**N**) Representative western blot and quantification of *O*-GlcNAc levels normalized to β-actin in pancreatic islets of 20 wo *Taz*-KD mice, N = 5. (**O**) Schematic figure of XF Mito Fuel Flex Test Kit protocol. The three inhibitors UK5099, etomoxir, and BPTES are used to inhibit glucose, fatty acid, and glutamine metabolism inside the mitochondria. By sequential addition of one or two of those inhibitors, nutrient dependencies and capacities of the corresponding pathways can be quantified. (**P**) OCR and **(R)** ECAR kinetic curves of 20 wo WT and *Taz*-KD pancreatic islets using sequential addition of BPTES (left), etomoxir (middle) or UK5099 (right). Starting with the addition of one of the inhibitors, followed by the addition of the remaining two to observe the nutrient dependency of the inhibited pathway. OCR (**Q**) and ECAR (**S**) kinetic curves of 20 wo WT and *Taz*-KD pancreatic islets using sequential addition of UK5099 and etomoxir (left), UK5099 and BTES (middle) or etomoxir and BPTES (right). Starting with the addition of two of the inhibitors, followed by the addition of the remaining one to observe the nutrient capacity of the non-inhibited pathway. n (WT) = 10, n (*Taz*-KD) = 6, n number of experiments include N = 4 (number of animals).

(**T**) Generation of a new mouse (shTaz x Mito-roGFP2-Orp1) model that expresses an shRNA against *Taz* and the mitochondrial H_2O_2 sensor roGFP2-Orp1. The mice are lifelong feed with doxy. Figure created with Biorender.com. (**U**) Imaging of real-time H_2O_2 titration (0 – 100 µM) kinetics of isolated pancreatic islets expressing the mito-roGFP2-Orp1 sensor. Ratio (excitation: 405/470 nm, emission: 500 – 530 nm) images (right, Lookup table: "Fire") created with ImageJ and reflect oxidation state at 0 µM (top), 100 µM H_2O_2 (middle) and 10 mmol/l DTT (bottom). Calibration bar: redox state from 0.3 (reduced) to 1.2 (oxidized), N = 3. (**V**) (Representative ratiometric image (ImageJ Lookup table: "Fire") of mito-roGFP2-Orp1/WT (left) and mito-roGFP2-Orp1/*Taz*-KD (right) pancreatic islets 50 wo. Scale bar: 100 µm. Right: Normalized percentage change in ratio of the redox state of the mito-roGFP2-Orp1 sensor in pancreatic islets of 50 wo mito-roGFP2-Orp1/WT and mito-roGFP2-Orp1/*Taz*-KD mice. N (WT, 50 wo) = 4, N (*Taz*-KD, 50 wo) = 4, N (DTT, 50 wo) = 6. (**W**) Representative western blot and quantification of Prx3 (left), Cat (middle) and GPX4 (right) normalized to β-actin in pancreatic islets of 20 wo *Taz*-KD mice, N (Prx3) = 4, N (Cat) = 3, N (GPX4) = 4, N (NOX4) = 4 and N (Nrf2) = 4, normalized to β-actin in pancreatic islets of 20 wo *Taz*-KD mice, (**X**) Paired western blot analysis of Prx3 (left), Cat (2nd left), GPX4 (3rd left), NOX4 (4th left) and NRF2 (right) normalized to β-actin in pancreatic islets of 20 wo *Taz*-KD mice, N (Prx3) = 4, N (Cat) = 3, N (GPX4) = 4, N (NOX4) = 4, N (Nrf2) = 4. (**y**) Representative western blot and quantification of ATF4 (left), total eIF2-alpha(2nd left), phosphor-eIF2-alpha and GDF-15 (right) normalized to β-actin in pancreatic islets of 20 wo *Taz*-KD mice, N (Prx3) = 4, (**z**) Paired western blot analysis of ATF4 (left), total eIF2-alpha(2nd left), phosphor-eIF2-alpha and GDF-15 (right) normalized to β-actin in pancreatic islets of 20 wo *Taz*-KD mice, N= 4-5. Data represent mean ± SEM (indicated by error bars); N and n numbers indicate number of animals and experiments, respectively; statistical significance was determined by unpaired Student *t* test: *p < 0.05, **p < 0.01. Abbreviations: Mitochondria-redox-sensitive-GFP2-Orp1 (Mito-roGFP2-Orp1), doxycycline (doxy), weeks of age (wo), *Tafazzin*-Knockdown (*Taz*-KD), Wildtype (WT), 2-deoxy-D-glucose (2DG), 2-deoxy-D-glucose-6-phosphate (2DG6P), glucokinase (GCK), glucose-6-phosphate dehydrogenase (G6PDH), oxygen consumption rate (OCR), extracellular acidification rate (ECAR), oligomycin (Oligo), antimycin A (Ant A), rotenone (Rot), Dithiothreitol (DTT), glucose (Glu, G), catalase (Cat), peroxiredoxin 3 (Prx3), glutathionperoxidase 4 (GPX4), area under the curve (AUC).

ESM Figure 03

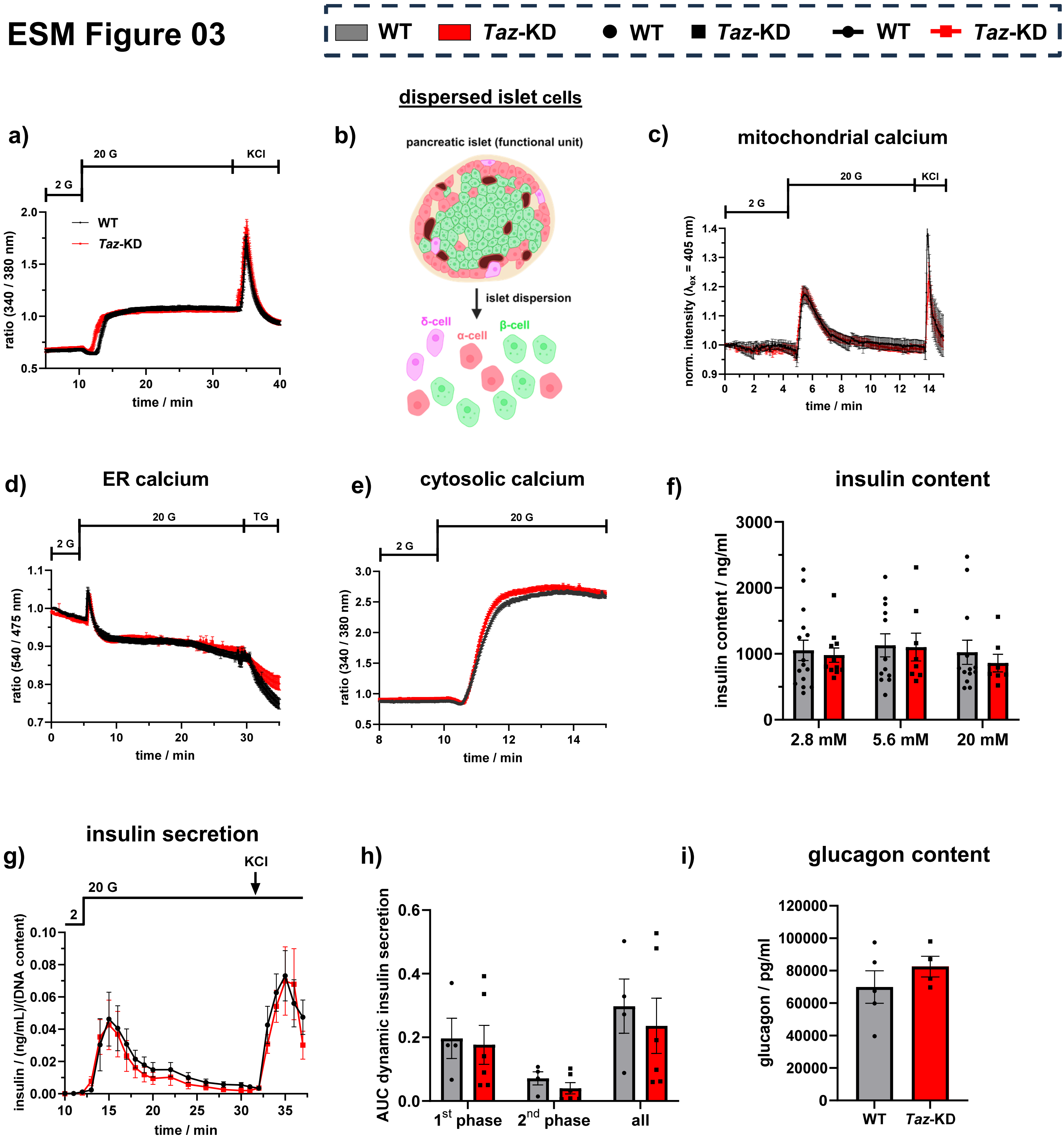

ESM Figure 03

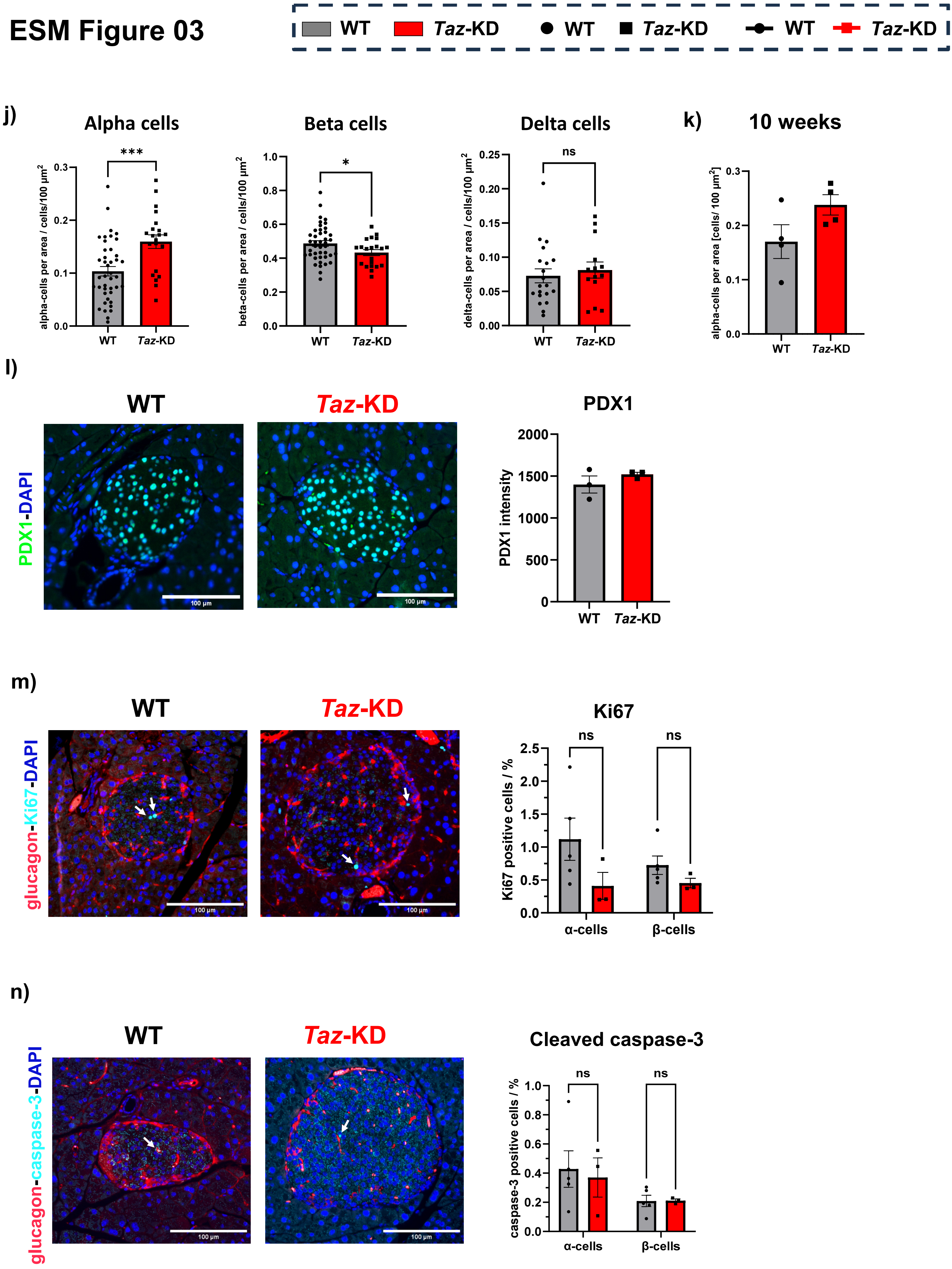

ESM Figure 3:

(**A**) Cytosolic calcium measurement of 20 wo WT and *Taz*-KD pancreatic islets using Fura-2 AM. The experiment represents the full-time course of the calcium experiment shown in Fig. 3 B. (**B**) Schematic illustration of pancreatic islet dispersion from whole pancreatic islets that act as a functional unit to single islet cells with limited connection between the cells. Created with Biorender.com. Mitochondrial (**C**), ER (**D**) and cytosolic (**E**) calcium levels of dispersed pancreatic islet cells from WT and *Taz*-KD 20 wo mice, measured with Mito-Pericam, D4ER and Fura-2 AM respectively. N (mitochondrial calcium, WT) = 4, N (mitochondrial calcium, *Taz*-KD) = 5, N (ER calcium, WT) = 4, N (ER calcium, *Taz*-KD) = 5, N (cytosolic calcium) = 3. (**F**) Quantification of insulin content of 20 wo WT and *Taz*-KD pancreatic islets at 2.8, 5.6 and 20 mmol/l glucose concentrations, N (WT) = 16, N (*Taz*-KD) = 17. (**G**) Dynamic GSIS (left) and (**H**) AUC quantification (right) of 20 wo WT and *Taz*-KD pancreatic islets normalized to DNA content, following the insulin levels at 2 mmol/l glucose, 20 mmol/l glucose and 30 mmol/l KCl conditions. AUC was separated in 1st (10 – 20 min) and 2nd (20 – 31 min) phase of insulin secretion, N (WT) = 4, N (*Taz*-KD) = 6. (**I**) Glucagon content of 20 wo WT and *Taz*-KD pancreatic islets, N (WT) = 5, N (*Taz*-KD) = 4. (**J**) Quantitative analysis of alpha (left), beta (middle) and delta (right) cell number of WT and *Taz*-KD pancreatic islets at 20 wo normalized to pancreatic islet area, n (alpha-cells, WT) = 42, n (alpha-cells, *Taz*-KD) = 21, n (beta-cells, WT) = 42, n (beta-cells, *Taz*-KD) = 21, n (detla-cells, WT) = 20, n (detla-cell, *Taz*-KD) = 14. (**K**) Quantitative analysis of alpha-cell number of WT and *Taz*-KD pancreatic islets at 10 wo normalized to pancreatic islet area, N = 4. Representative images and intensity quantification of IHC against PDX1 (**L**), Ki67 (**M**) and Cleaved Caspase3 (**N**) in *Taz*-KD and WT pancreatic islets at 20 wo. Scale bar: 100 µm. N (PDX1) = 3, N (Ki67 and cleaved caspase-3, WT) = 5, N (Ki67 and cleaved caspase-3, *Taz*-KD) = 3. Data represent mean ± SEM (indicated by error bars); n and N numbers indicate number of experiments and animals; statistical significance was determined by unpaired Student *t* test: $*p < 0.05$, $**p < 0.01$, $***p < 0.001$. Abbreviations: weeks of age (wo), *Tafazzin*-Knockdown (*Taz*-KD), Wildtype (WT), oxygen consumption rate (OCR), extracellular acidification rate (ECAR), oligomycin (Oligo), basal rate (BR), immol/lunohistochemistry (IHC), Pancreatic and duodenal homeobox 1 (PDX1), Glucose-stimulated-insulin-secretion (GSIS), area under the curve (AUC).

ESM Figure 04

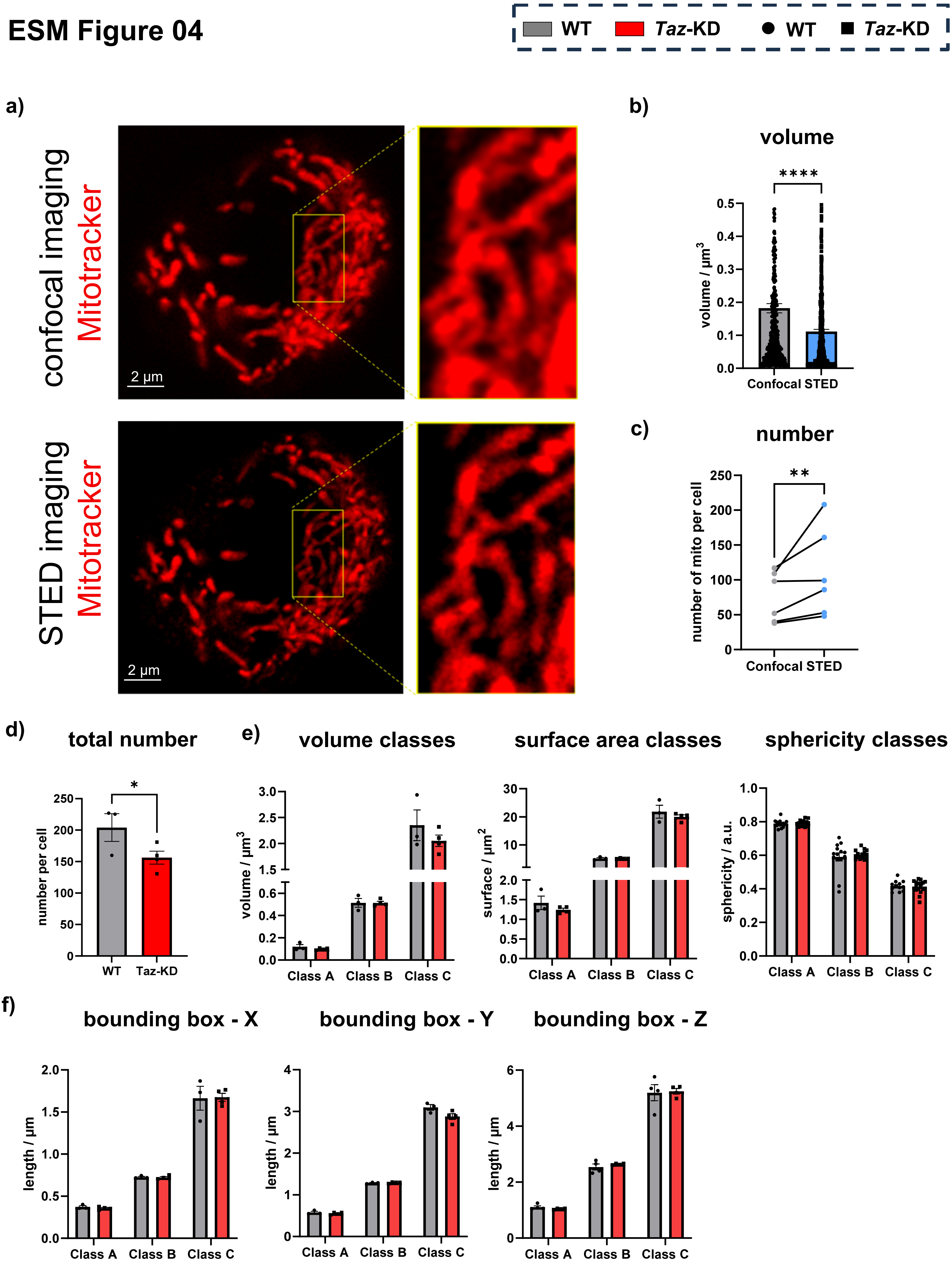

ESM Figure 04

● WT ■ *Taz*-KD

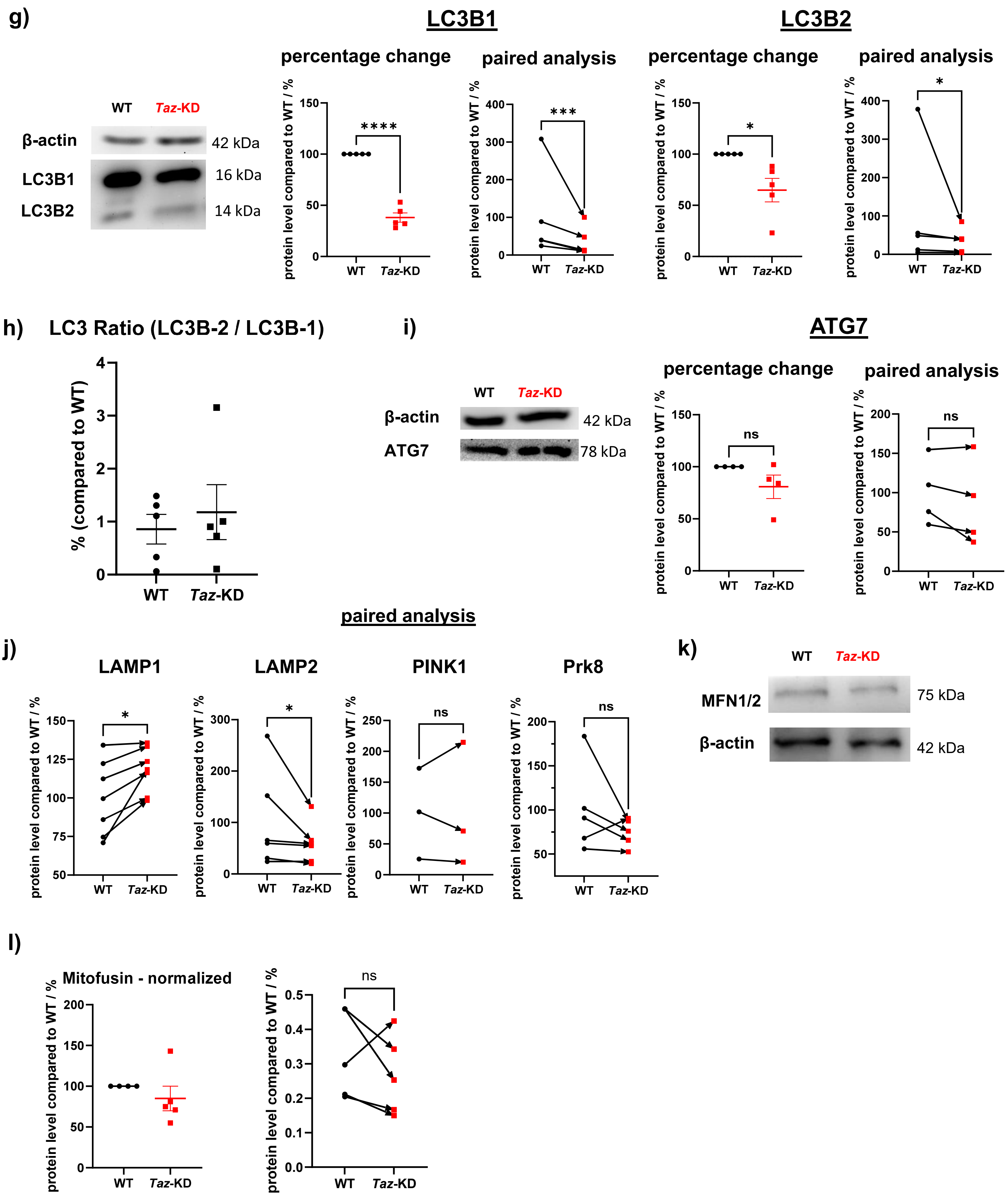

ESM Figure 4:

(**A**) Representative images of confocal (top left) and STED (bottom left) microscopy of mitochondrial network of the same pancreatic islet cell. Scale bar: 2 µm. (**B**) Comparison of single mitochondrial volume and number (**C**) of confocal (black) and STED (blue) imaging. n = 6. (**D**) Total mitochondrial number per pancreatic islet cells from 20 wo WT and *Taz*-KD mice. N (WT) = 3, N (*Taz*-KD) = 4. Single mitochondrion (**E**) and bounding box (**F**) analysis separated by three surface area classes (A = 0.3 – 3 $µm^2$, B = 3 – 10 $µm^2$, C > 10 $µm^2$) of 20 wo WT and *Taz*-KD dispersed pancreatic islet cells. The single mitochondria analysis includes the parameters volume (left), surface area (middle), sphericity (right) and the bounding box analysis includes the length in the three dimensions X (left), Y (middle), Z (right), N (WT) = 3, N (*Taz*-KD) = 4. Representative western blot, calculated percentage change and paired western blot analysis of LC3B-1 (left) and LC3B-2 (right) (**G**), and calculated ratio (**H**) and Atg7 (**I**) protein levels from 20 wo WT and *Taz*-KD pancreatic islets compared to β-actin protein level, N (LC3B) = 5, N (Atg7) = 4. (**J**) Paired western blot analysis of LAMP1 (left), LAMP2 (2nd left), PINK1 (3rd left) and Prk8 (right) normalized to β-actin in pancreatic islets of 20 wo *Taz*-KD mice, N (LC3B-2) = 5, N (LAMP1) = 7, N (LAMP2) = 6, N (PINK1) = 3, N (Prk8) = 5. (**K**) Representative western blot showing protein levels of Mitofusin from 20 wo WT and *Taz*-KD pancreatic islets compared to beta-actin protein level. (**L**) Calculated percentage change and paired western blot analysis of Mitofusin 1/2 in pancreatic islets of 20 wo *Taz*-KD mice, N = 5. Data represent mean ± SEM (indicated by error bars); N numbers indicate number of animals; statistical significance was determined by unpaired or paired (western blot) Student *t* test: *p < 0.05, ***p < 0.001, ****p < 0.0001. Abbreviations: weeks of age (wo), *Tafazzin*-Knockdown (*Taz*-KD), Wildtype (WT), lysosomal-associated membrane protein 1 (LAMP1), lysosomal-associated membrane protein 2 (LAMP2), parkin (Prk8), Mfn (Mitofusin 1 and 2).

ESM Figure 05

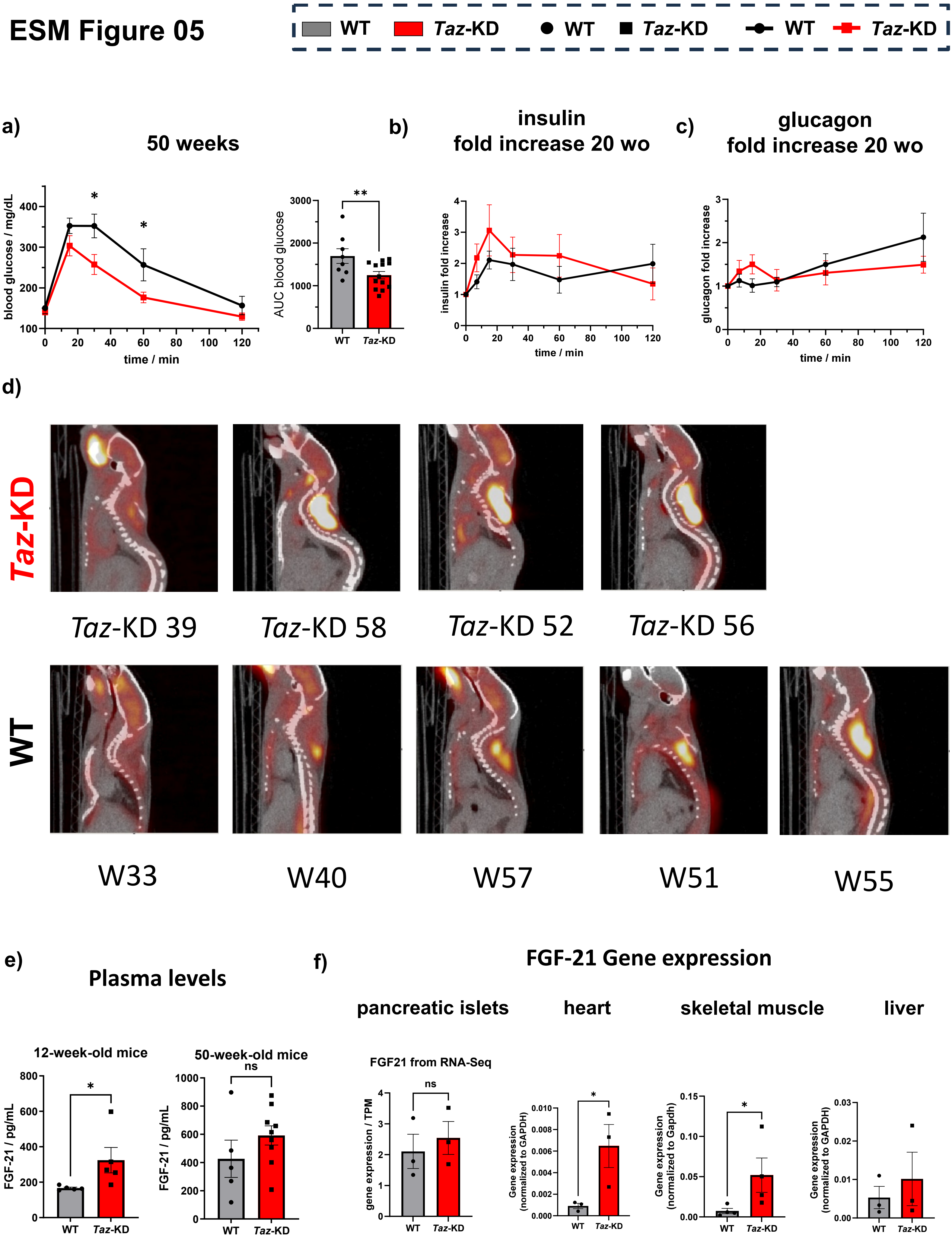

ESM Figure 5:

(**A**) Blood glucose levels (left) and quantified AUC (right) of *Taz*-KD and WT mice at 50 wo during i.p. GTT, N (50 wo, WT) = 8, N (50 wo, *Taz*-KD) = 13. Quantified fold increase of plasma insulin (**C**) and glucagon (**D**) from GTT of 20 wo WT and *Taz*-KD mice, N (plasma insulin and glucagon, WT) = 7, N (plasma insulin and glucagon, *Taz*-KD) = 8. (**D**) PET-CT images of BAT 18F-FDG uptake in WT and *Taz*-KD mice of 20 wo. N (20 wo, WT) = 5, N (20 wo, *Taz*-KD) = 4. (**E**) Plasma levels of FGF-21 in in WT and *Taz*-KD mice of 12 and 50 wo. N (12 wo, WT) = 5, N (12 wo, *Taz*-KD) = 5; N (12 wo, WT) = 5, N (12 wo, *Taz*-KD) = 9. (**F**) FGF-21 expression levels from islets (data from RNAseq), heart, skeletal muscle and liver from WT and *Taz*-KD mice of 20 wo. N for islets, heart and liver :(20 wo, WT) = 3, N (20 wo, *Taz*-KD) = 3; for skeletal muscle N (20 wo, WT) = 4, N (20 wo, *Taz*-KD) = 4. Data represent mean ± SEM (indicated by error bars); N numbers indicate number of animals; statistical significance was determined by unpaired Student *t* test: *p < 0.05, **p < 0.01. Abbreviations: weeks of age (wo), *Tafazzin*-Knockdown (*Taz*-KD), Wildtype (WT), glucose tolerance test (GTT), area under the curve (AUC), brown adipose tissue (BAT).

ESM Figure 06

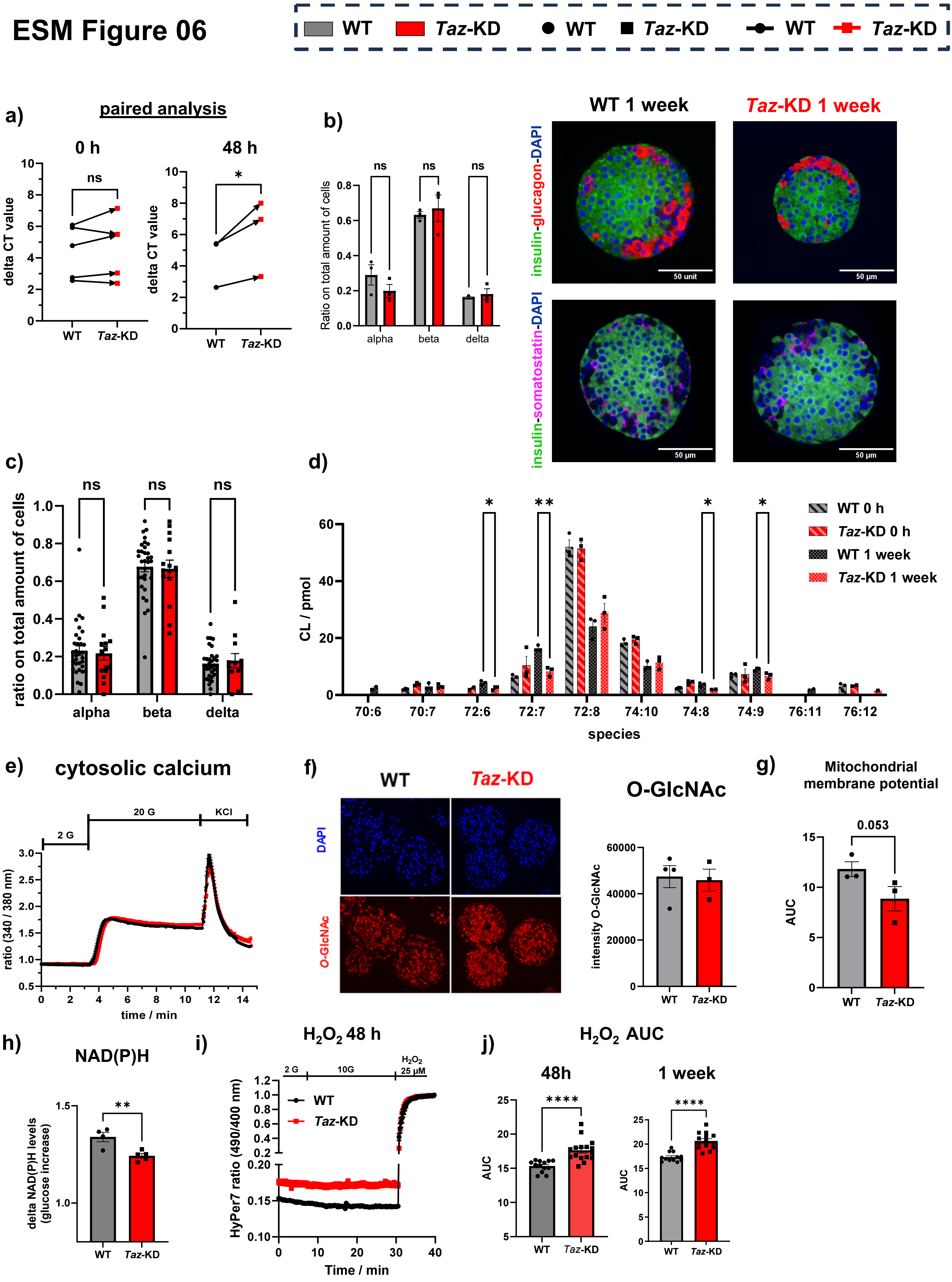

ESM Figure 06

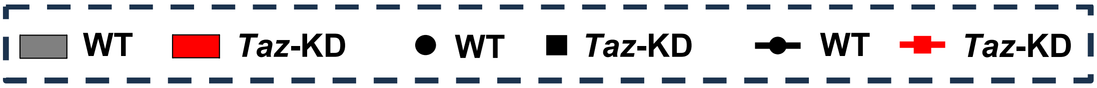

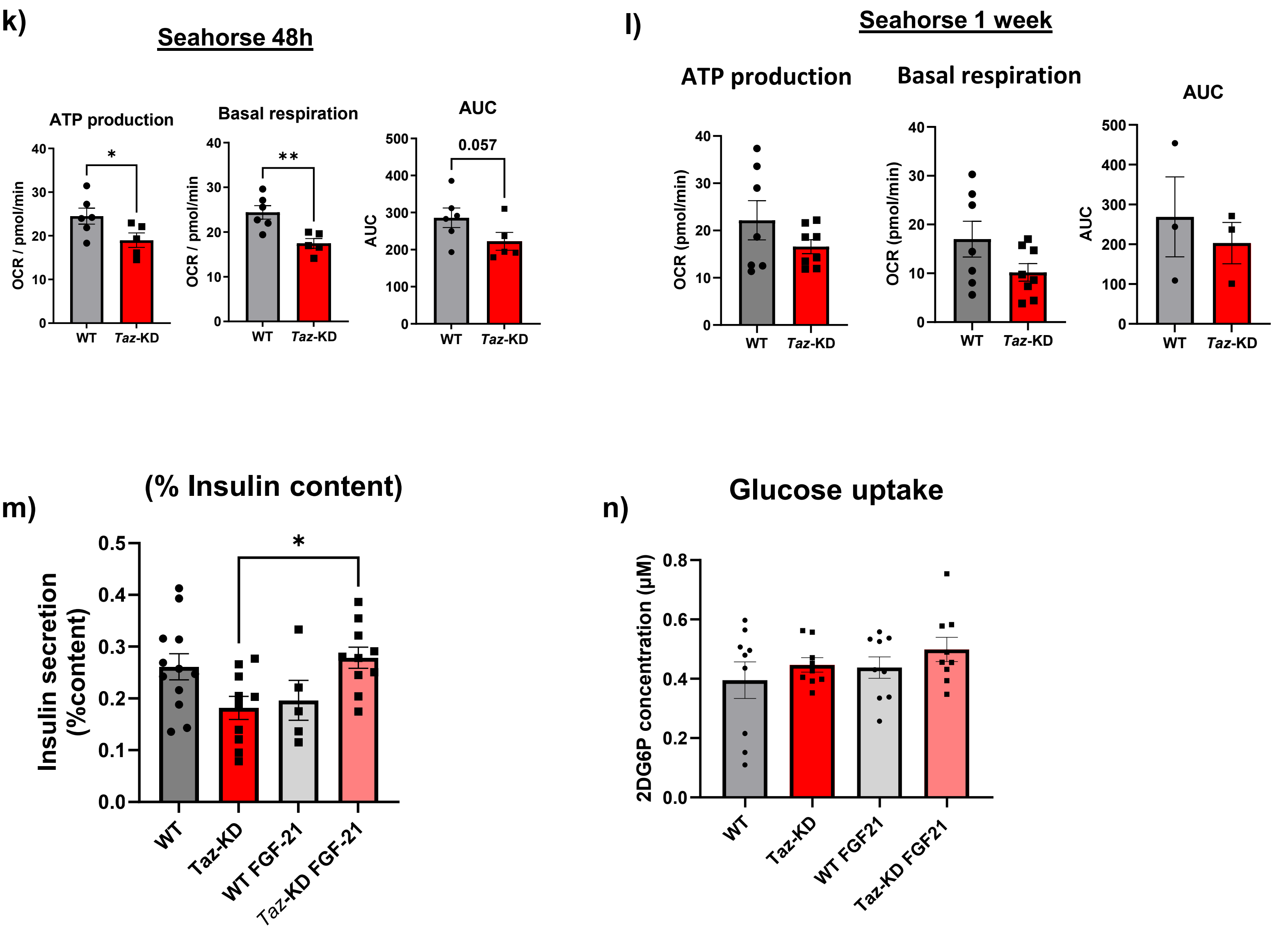

ESM Figure 6:

(**A**) Paired qPCR analysis of *Taz* gene expression in WT and *Taz*-KD pancreatic islets directly after isolation (0 h) and 48 h after doxy culture. N (0 h) = 5, N (48 h) = 3. (**B**) Quantification and representative IHC images of isolated and paraffin embedded WT (left) and *Taz*-KD (right) pancreatic islets after 1 week doxy culture. Pancreatic islets were stained against insulin (green) - glucagon (red) (top panel) or insulin (green) - somatostatin (magenta) (bottom panel) together with DAPI (blue). Scale bar: 100 µm. (**C**) Single values of the quantitative ImageJ analysis of alpha-, beta- and delta-cell number using IHC on paraffin embedded pancreatic islets and counting DAPI spots of WT and *Taz*-KD pancreatic islets after 1 week of doxy incubation, n (WT) = 32, n (*Taz*-KD) = 15. (**D**) CL species profile of *Taz*-KD and WT pancreatic islets directly after isolation (0 h) and after 1 week of doxy incubation, N = 3, some replicates are below the limit of detection. (**E**) Cytosolic calcium levels of WT and *Taz*-KD pancreatic islets after 1 week doxy incubation in 2 mmol/l and 20 mmol/l glucose concentration. 30 mmol/l of KCl was added as a positive control, N = 6. AUC for (F) Representative images of O-GlcNAC (red) and DAPI (blue) staining's in WT and *Taz*-KD islets after 1 week doxy treatment in vitro (left), O-GlcNAC Intensity Quantification (right) N WT= 4 and N *Taz*-KD = 3. (**G**) mitochondrial membrane potential N= 3 and (**H**) NAD(P)H levels N WT= 4 and N *Taz*-KD = 5. (**I**) H_2O_2 levels normalized to maximal response (25 µmol/l H_2O_2) after 48 hours *Taz* knockdown *in vitro* N= 4. (**J**) AUC of H_2O_2 levels, excluding maximal response after 48 hours or 1 week doxy treatment n= 12-16 islets from N= 3 (1 week) -4 animals (48 hours). Quantification of ATP, basal respiration and AUC of the OCR measurements from 48 hours (**K**) and 1 week (**L**) *Taz*-KD N= 4 (48 hours) and N= 3 (1 week). (**M**) Insulin secretion normalized by insulin content (% of content) in 20 mmol/l glucose in WT and *Taz*-KD pancreatic islets after 1-week doxy incubation with or without 50 nmol/l FGF-21. n= 5 -12 wells with 5 islets/each. N= 4 animals for all conditions. (**N**) Glucose uptake in WT and *Taz*-KD pancreatic islets after 1-week doxy incubation with or without 50 nmol/l FGF-21, indicated by the levels of 2DG6P N= 3 animals for all conditions, in triplicate n= 9. Data represent mean ± SEM (indicated by error bars); N numbers indicate number of animals, n= experiments; statistical significance was determined by unpaired Student *t* test: *p < 0.05, **p < 0.01. Abbreviations: adjusted p-values (padj), gene ontology (GO), weeks of age (wo), *Tafazzin*-Knockdown (*Taz*-KD), Wildtype (WT), doxycycline (doxy), immol/lunohistochemistry (IHC), Cardiolipin (CL).

Supplements to Figure 07

a)

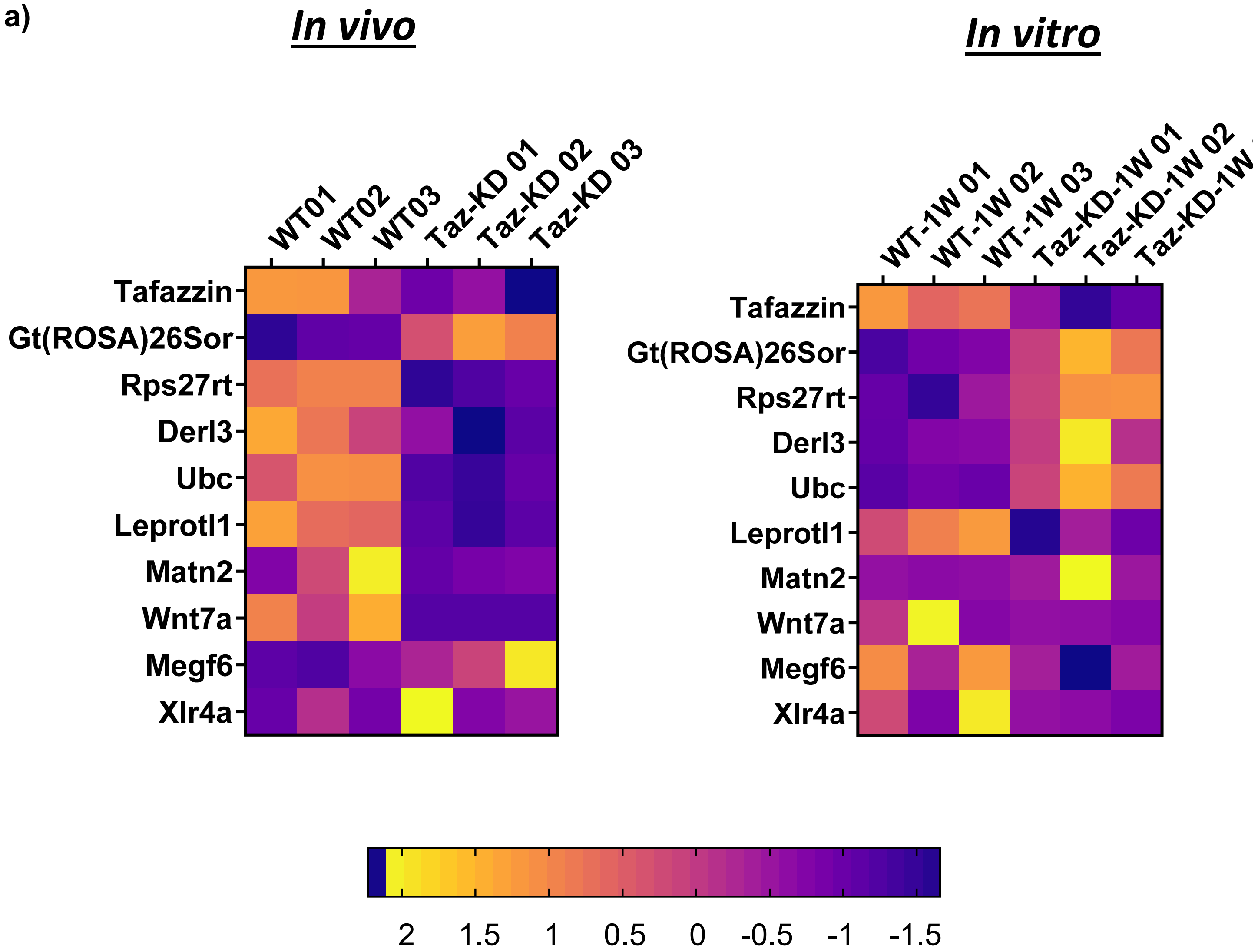

ESM Figure 7:

Bulk mRNA sequencing of 20 wo WT and *Taz*-KD pancreatic islets revealed 10 DEG which are significantly changed compared to their respective WT control group in *in vivo* (left) and *in vitro* (right) *Taz* knockdown, N = 3. Abbreviations: weeks of age (wo), *Tafazzin*-Knockdown (*Taz*-KD), Wildtype (WT), differentially expressed genes (DEG).
